# Relative efficacy of five SGLT2 inhibitors: a network meta-analysis of 20 cardiovascular and respiratory outcomes

**DOI:** 10.3389/fphar.2024.1419729

**Published:** 2024-06-12

**Authors:** LiGang Huang, Rong Hu, HaiTao Zou

**Affiliations:** ^1^ Department of Cardiovascular Medicine, Dianjiang People’s Hospital of Chongqing, Chongqing, China; ^2^ Department of Respiratory and Critical Care Medicine, Dianjiang People’s Hospital of Chongqing, Chongqing, China

**Keywords:** SGLT2 inhibitors (gliflozins), heart failure, myocardial infarction, pneumonia, asthma, complete atrioventricular block, acute respiratory failure, hypertensive crisis

## Background

More and more clinical guidelines have recommended use of sodium-glucose cotransporter-2 (SGLT2) inhibitors, also known as gliflozins, to prevent major adverse cardiovascular and renal events in patients with type 2 diabetes, heart failure, and/or chronic kidney disease. The mechanisms by which SGLT2 inhibitors exert cardiovascular and renal benefits include but are not limited to: the anti-inflammatory effects by the potential pathways (e.g., mitochondrial, oxidative stress, and inflammasome pathways) (Mashayekhi et al., 2024), the modulation of cellular energy metabolism, and housekeeping mechanisms (Preda et al., 2024). Since there have not been head-to-head cardiovascular outcome randomized controlled trials (RCTs) comparing a gliflozin with one other gliflozin, the relative efficacy of various gliflozins on cardiovascular outcomes has not been established. Several published network meta-analyses (Kanie et al., 2021; Lin et al., 2021; Giugliano et al., 2022; Zhang et al., 2022; Brønden et al., 2023) assessing the cardiovascular outcomes of SGLT2 inhibitors have compared SGLT2 inhibitors with non-gliflozin drugs, such as glucagon-like peptide-1 receptor agonists, dipeptidyl peptidase-4 inhibitors, and finerenone; but have not performed the comparisons among various gliflozins. Moreover, these studies (Kanie et al., 2021; Lin et al., 2021; Giugliano et al., 2022; Zhang et al., 2022; Brønden et al., 2023) of network meta-analysis have focused on evaluating those composite cardiovascular outcomes, such as major adverse cardiovascular events (defined as a composite of cardiovascular death, myocardial infarction, and stroke), but have evaluated a limited number of individual cardiovascular outcomes. Some important individual outcomes, such as complete atrioventricular block and hypertensive crisis, have not been evaluated in any of these studies (Kanie et al., 2021; Lin et al., 2021; Giugliano et al., 2022; Zhang et al., 2022; Brønden et al., 2023). Therefore, we included those large-scale placebo-controlled RCTs of SGLT2 inhibitors to carry out a network meta-analysis, aiming to assess the relative efficacy of various gliflozins on various individual cardiovascular outcomes.

In recent years, more and more real-world studies have revealed the obvious benefits of SGLT2 inhibitors against many respiratory diseases, such as chronic obstructive pulmonary disease (COPD) (Pradhan et al., 2022), pneumonia (Wu et al., 2022), obstructive airway disease (Au et al., 2023), pulmonary edema (Jeong et al., 2023), and respiratory failure (Jeong et al., 2023). Similar with these real-world evidences, several meta-analyses (Yin et al., 2021; Li et al., 2022; Wang et al., 2022; Wang and Zhou, 2024; Xiao et al., 2024) based on RCTs have also revealed the respiratory system benefits of SGLT2 inhibitors. A meta-analysis (Yin et al., 2021) of 9 RCTs showed that use of gliflozins was significantly associated with the lower risks of multiple respiratory diseases, such as COPD, and asthma. One other meta-analysis (Xiao et al., 2024) of 14 RCTs and another meta-analysis (Wang and Zhou, 2024) of 32 RCTs produced the substantially consistent findings. Moreover, two other studies (Li et al., 2022; Wang et al., 2022) of meta-analysis based on RCTs revealed that use of gliflozins was significantly associated with less pneumonia and asthma, respectively. These aforementioned findings seem to suggest that it is relatively certain for gliflozins to exert the effects against relevant respiratory diseases. However, it has been unclear whether there are obvious differences among various gliflozins in the effects against respiratory diseases. Therefore, in this network meta-analysis we would also assess the relative efficacy of different gliflozins on various respiratory outcomes.

## Methods

This network meta-analysis was done according to the Preferred Reporting Items for Systematic Reviews and Meta-Analyses (PRISMA) extension statement for network meta-analyses (Hutton et al., 2015). A systematic search was performed using PubMed and ClinicalTrials.gov for relevant articles published before 21 August 2023. Our search strategy used in this network meta-analysis was as follows: ((Canagliflozin [TIAB] OR Dapagliflozin [TIAB] OR Empagliflozin [TIAB] OR Ertugliflozin [TIAB] OR Sotagliflozin [TIAB] OR Canagliflozin [MH] OR Dapagliflozin [Supplementary Concept] OR Empagliflozin [Supplementary Concept] OR Ertugliflozin [Supplementary Concept] OR LX4211 [TIAB] OR LX-4211 [TIAB]) AND (randomized controlled trial [PT] OR randomized controlled trial [TI] OR clinical trial [PT] OR clinical trial [TI] OR random* [TIAB] OR trial* [TIAB] OR placebo [TIAB] OR drug therapy [MH] OR drug therapy [SH]) AND humans [MH]) NOT (review* [TI] OR review* [PT] OR meta-analysis [TI] OR meta-analysis [PT]). Our search strategy had no limit on literature language.

In this network meta-analysis, we included those studies that were the RCTs having enrolled ≥ four hundred subjects, having compared any of the five SGLT2 inhibitors (i.e., canagliflozin, dapagliflozin, empagliflozin, ertugliflozin, and sotagliflozin) with placebo, and having reported any of the serious adverse events (SAEs) of interest. The SAEs of interest in this network meta-analysis were 20 kinds of cardiovascular and respiratory diseases, consisting of 10 cardiovascular diseases (namely, Myocardial infarction, Cardiac failure, Cardiac failure chronic, Cardiac failure congestive, Atrioventricular block complete, Cardiac failure acute, Coronary artery disease, Hypertensive crisis, Hypertensive emergency, and Hypertension) and 10 respiratory diseases (namely, Acute respiratory failure, Pulmonary oedema, COPD, Pulmonary hypertension, Dyspnoea, Asthma, Respiratory tract infection, Lower respiratory tract infection [LRTI], Pneumonia, and Pneumonia bacterial). We excluded those RCTs published in Letter to the Editor or Research Letter, but did not exclude those published in Short Original Article, Brief Report, or Rapid Communications. Two authors independently extracted the outcome data from ClinicalTrials.gov and extracted the other information (e.g., first author, publication year, and participant characteristics) from the full texts of included studies. Furthermore, two authors independently evaluated the quality of included RCTs according to the Cochrane risk of bias assessment tool (Higgins et al., 2011). Any disagreements between them would be addressed by discussion between them and a third author.

Using the two-category data (i.e., the numbers of events and subjects in each study arm), we did network meta-analyses on all of the 20 outcomes of interest. In order to derive the conservative results, we used a random-effects model to perform network meta-analysis. The effect sizes for pairwise comparisons among various SGLT2 inhibitors were given in the forest plots showing risk ratios (RRs) and their 95% confidence intervals (CIs). We drew network plots to present the network of comparisons, and drew funnel plots to assess the potential publication bias. We drew surface under the cumulative ranking curve (SUCRA) plots to present the relative rankings of various gliflozins for each outcome. More importantly, we also drew radar plots, in order that a radar plot could present lots of SUCRA values for many outcomes, with a greater SUCRA value meaning a greater probability for reducing adverse events. *p* < 0.05 represented for statistical significance. All of the statistical analyses and the statistical plots were done using the Stata/MP 16.0 software.

## Results

At first, we identified 2704 records. After study selection (Supplementary Figure S1), we finally included 28 articles reporting a total of 29 RCTs. The ClinicalTrials.gov identification numbers of these included trials were NCT03594110 [EMPA-KIDNEY] (Herrington et al., 2023), NCT03242252 [SOTA-CKD3] (Cherney et al., 2023), NCT03521934 [SOLOIST-WHF] (Bhatt et al., 2021a), NCT04157751 [EMPULSE] (Voors et al., 2022), NCT03315143 [SCORED] (Bhatt et al., 2021b), NCT04252287 [CHIEF-HF] (Spertus et al., 2022), NCT03619213 [DELIVER] (Solomon et al., 2022), NCT01986855 [VERTIS RENAL] (Grunberger et al., 2018), NCT03057951 [EMPEROR-Preserved] (Anker et al., 2021), NCT00528879 (Bailey et al., 2010), NCT03036124 [DAPA-HF] (McMurray et al., 2019), NCT00673231 (Wilding et al., 2012), NCT03036150 [DAPA-CKD] (Heerspink et al., 2020), NCT02384941 [inTandem1] (Buse et al., 2018), NCT03057977 [EMPEROR-Reduced] (Packer et al., 2020), NCT01210001 [EMPA-REG EXTEND] (Kovacs et al., 2015), NCT01730534 [DECLARE–TIMI 58] (Wiviott et al., 2019), NCT01011868 (Tuttle et al., 2022), NCT02065791 [CREDENCE] (Perkovic et al., 2019), NCT01031680 (Cefalu et al., 2015), NCT04350593 [DARE-19] (Kosiborod et al., 2021), NCT01131676 [EMPA-REG OUTCOME] (Zinman et al., 2015), NCT01042977 (Leiter et al., 2016), NCT01032629 [CANVAS] (Neal et al., 2017), NCT01989754 [CANVAS-R] (Neal et al., 2017), NCT01106651 (Januzzi et al., 2017), NCT01164501 [EMPA-REG RENAL] (Barnett et al., 2014), NCT01986881 [VERTIS CV] (Cannon et al., 2020), and NCT01106625 [CANTATA-MSU] (Polidori et al., 2014), respectively. The detailed characteristics of these included trials are shown in Supplementary Table S1. These included RCTs were with low risk of bias in general, and enrolled a total of 100740 participants, including 54735 taking SGLT2 inhibitors and 46005 taking placebo. A total of five gliflozins were involved in this network meta-analysis, and they were dapagliflozin (N = 18791), empagliflozin (N = 14186), canagliflozin (N = 9004), ertugliflozin (N = 5806), and sotagliflozin (N = 6948), respectively.

**FIGURE 1:**
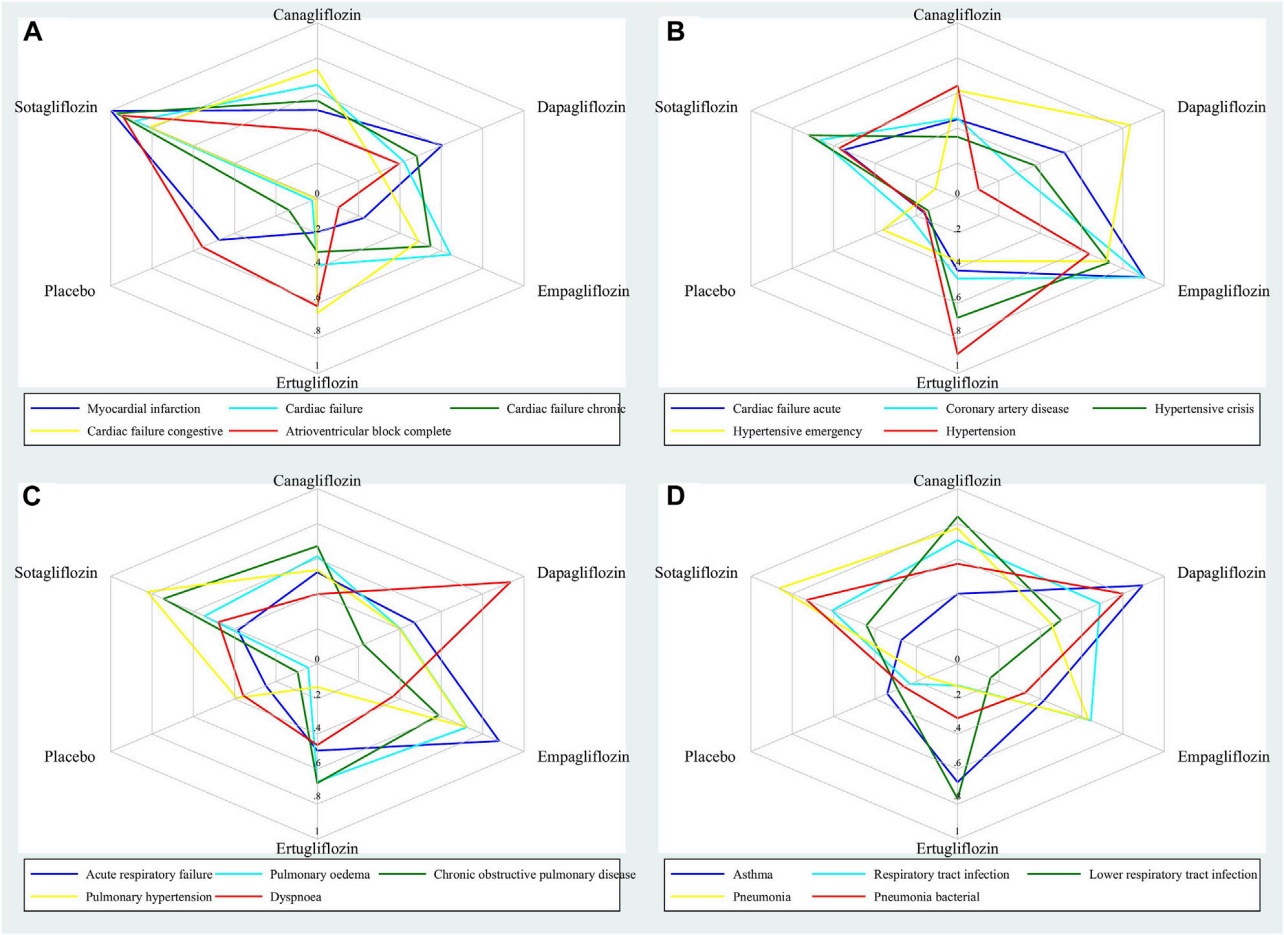
Radar plots showing the SUCRA values of five gliflozins for 20 cardiovascular and respiratory outcomes.

Figure 1 shows the SUCRA values of five gliflozins for the 20 cardiovascular and respiratory outcomes assessed in this network meta-analysis. Sotagliflozin had the greatest SUCRA values for reducing Myocardial infarction, Cardiac failure, Cardiac failure chronic, Cardiac failure congestive, and Atrioventricular block complete (Figure 1A). Empagliflozin had the greatest SUCRA values for reducing Cardiac failure acute, Coronary artery disease, and Hypertensive crisis; dapagliflozin had the greatest SUCRA value for reducing Hypertensive emergency; and ertugliflozin had the greatest SUCRA value for reducing Hypertension (Figure 1B). Empagliflozin had the greatest SUCRA values for reducing Acute respiratory failure, and Pulmonary oedema; sotagliflozin had the greatest SUCRA values for reducing COPD, and Pulmonary hypertension; and dapagliflozin had the greatest SUCRA value for reducing Dyspnoea (Figure 1C). Dapagliflozin had the greatest SUCRA values for reducing Asthma, and Pneumonia bacterial; canagliflozin had the greatest SUCRA values for reducing Respiratory tract infection, and LRTI; and sotagliflozin had the greatest SUCRA value for reducing Pneumonia (Figure 1D). The detailed SUCRA plots of five gliflozins for all outcomes are provided in Supplementary Figures S2–S21.

The results of network meta-analyses on 20 cardiovascular and respiratory outcomes are presented in the forest plots (Supplementary Figures S22–S41). Sotagliflozin significantly reduced Myocardial infarction compared with placebo (RR 0.49, 95% CI 0.32-0.74), ertugliflozin (RR 0.41, 95% CI 0.23-0.70), empagliflozin (RR 0.43, 95% CI 0.27-0.70), dapagliflozin (RR 0.52, 95% CI 0.32-0.84), and canagliflozin (RR 0.50, 95% CI 0.29-0.85) (Supplementary Figure S22). Sotagliflozin (RR 0.62, 95% CI 0.51-0.76), empagliflozin, dapagliflozin, and canagliflozin significantly reduced Cardiac failure compared with placebo (Supplementary Figure S23). Sotagliflozin significantly reduced Cardiac failure chronic compared with placebo (RR 0.33, 95% CI 0.17-0.64), ertugliflozin (RR 0.35, 95% CI 0.14-0.90), empagliflozin (RR 0.45, 95% CI 0.20-0.97), and dapagliflozin (RR 0.42, 95% CI 0.20-0.90) (Supplementary Figure S24). Sotagliflozin (RR 0.57, 95% CI 0.37-0.86), empagliflozin, dapagliflozin, and canagliflozin significantly reduced Cardiac failure congestive compared with placebo (Supplementary Figure S25). Sotagliflozin significantly reduced Atrioventricular block complete compared with placebo (RR 0.36, 95% CI 0.13-0.99), empagliflozin (RR 0.17, 95% CI 0.05-0.61), and dapagliflozin (RR 0.30, 95% CI 0.10-0.94) (Supplementary Figure S26). Empagliflozin and dapagliflozin significantly reduced Cardiac failure acute compared with placebo (Supplementary Figure S27). Empagliflozin significantly reduced Coronary artery disease compared with placebo (Supplementary Figure S28). Empagliflozin significantly reduced Hypertensive crisis compared with placebo (Supplementary Figure S29). Dapagliflozin significantly reduced Hypertensive emergency compared with placebo and sotagliflozin (Supplementary Figure S30). Ertugliflozin significantly reduced Hypertension compared with dapagliflozin (RR 0.30, 95% CI 0.11-0.83) and placebo (Supplementary Figure S31). Empagliflozin significantly reduced Acute respiratory failure compared with placebo (Supplementary Figure S32). Empagliflozin, dapagliflozin, and canagliflozin significantly reduced Pulmonary oedema compared with placebo (Supplementary Figure S33). Empagliflozin significantly reduced COPD compared with placebo (Supplementary Figure S34). Empagliflozin significantly reduced Pulmonary hypertension compared with placebo (Supplementary Figure S35). Dapagliflozin significantly reduced Dyspnoea compared with placebo and empagliflozin (Supplementary Figure S36). Dapagliflozin significantly reduced Asthma compared with placebo (Supplementary Figure S37). Canagliflozin significantly reduced Respiratory tract infection compared with placebo (Supplementary Figure S38). Canagliflozin significantly reduced LRTI compared with placebo and empagliflozin (Supplementary Figure S39). Sotagliflozin (RR 0.69, 95% CI 0.50-0.97), empagliflozin, and canagliflozin significantly reduced Pneumonia compared with placebo (Supplementary Figure S40). Sotagliflozin (RR 0.43, 95% CI 0.25-0.75) and dapagliflozin significantly reduced Pneumonia bacterial compared with placebo (Supplementary Figure S41).

The network plots of 20 outcomes are given in Supplementary Figures S42–S61. These plots suggested that all of the included studies were placebo-controlled trials and there was no direct evidence among various gliflozins. Therefore, there was no need to perform inconsistency test. Furthermore, the funnel plots of 20 outcomes (Supplementary Figures S62–S81) showed that many of the included studies were located at the top of the funnel plots, suggesting that many of the included studies had a relatively large sample size. Meanwhile, all of funnel plots were substantially symmetrical. This suggested, it was unlikely that there was any potential publication bias in this network meta-analysis.

## Discussion

This is the first network meta-analysis which aimed to assess the relative efficacy of various SGLT2 inhibitors on various cardiovascular and respiratory outcomes. This network meta-analysis produced several key findings as follows.

First, sotagliflozin *versus* placebo significantly reduced Myocardial infarction, Cardiac failure, Cardiac failure chronic, Cardiac failure congestive, Atrioventricular block complete, and Pneumonia; and sotagliflozin had the greatest SUCRA values for reducing these six outcomes. These suggest that sotagliflozin may be the best gliflozin for preventing these cardiopulmonary outcomes. The findings regarding heart failure and myocardial infarction in our network meta-analysis are consistent with those in previous network meta-analyses (Qiu et al., 2022; Tornyos et al., 2022; Kongmalai et al., 2023; Li et al., 2023): Li et al. (Li et al., 2023) identified that sotagliflozin had the best efficacy in reducing heart failure events; Kongmalai et al. (Kongmalai et al., 2023) identified that sotagliflozin had the highest probability of reducing the composite outcome of heart failure hospitalization and cardiovascular death; Tornyos et al. (Tornyos et al., 2022) identified that sotagliflozin seemed to be more effective regarding composite heart failure outcome and myocardial infarction than ertugliflozin; and Qiu et al. (Qiu et al., 2022) identified that the maximum SUCRA value accompanied sotagliflozin in reducing myocardial infarction.

Second, empagliflozin *versus* placebo significantly reduced Cardiac failure acute, Coronary artery disease, Hypertensive crisis, Acute respiratory failure, Pulmonary oedema, COPD, and Pulmonary hypertension; and empagliflozin had the greatest SUCRA values for reducing the former five outcomes. These suggest that empagliflozin may be the best gliflozin for preventing these cardiopulmonary outcomes. The findings regarding acute heart failure in our network meta-analysis are similar with the results of the EMPULSE trial (Kosiborod et al., 2022; Voors et al., 2022): initiation of empagliflozin in patients hospitalized for acute heart failure was safe in general and led to significant clinical benefit (Voors et al., 2022), and that benefit was not affected by the degree of symptomatic impairment at baseline (Kosiborod et al., 2022). In spite of this, the efficacy of empagliflozin in acute heart failure on long-term cardiovascular outcomes (e.g., cardiovascular death, worsening heart failure, and rehospitalization for heart failure) needs further investigation. Similar with the findings regarding pulmonary hypertension and COPD in our study, an animal study (Chowdhury et al., 2020) showed that empagliflozin attenuated maladaptive pulmonary remodeling, reduced right ventricle systolic pressure, and lowered the risk of death in experimental rats with pulmonary arterial hypertension. Furthermore, gliflozins could exert the beneficial effects against COPD and CO_2_ retention by reducing serum glucose level and then reducing the generation of endogenous CO_2_ (Brikman and Dori, 2020).

Third, dapagliflozin *versus* placebo significantly reduced Hypertensive emergency, Asthma, Dyspnoea, and Pneumonia bacterial; and dapagliflozin had the greatest SUCRA values for reducing these four outcomes. These suggest that dapagliflozin may be the best gliflozin for preventing these cardiopulmonary outcomes. What supports the anti-asthma activity of dapagliflozin is, Tabaa and others (Tabaa et al., 2022) carried out a rigorous animal experiment, and came to the following conclusion: since dapagliflozin had anti-oxidant, anti-inflammatory, and bronchodilator properties, this gliflozin might present a novel promising possibility for the treatment of asthma.

Last, canagliflozin *versus* placebo significantly reduced Respiratory tract infection and LRTI; and canagliflozin had the greatest SUCRA values for reducing these two outcomes. These suggest that canagliflozin may be the best gliflozin for preventing these respiratory outcomes. Meanwhile, ertugliflozin significantly reduced Hypertension compared with dapagliflozin and placebo; and ertugliflozin had the greatest SUCRA value for reducing this outcome. These suggest that ertugliflozin may be the best gliflozin for preventing Hypertension. Similarly, a *post hoc* pooled analysis of three phase 3 RCTs of ertugliflozin identified that ertugliflozin *versus* placebo resulted in statistically significant reductions in systolic blood pressure and diastolic blood pressure (Liu et al., 2019). Moreover, one of the underlying mechanisms for the effect of canagliflozin against LRTI (e.g., pneumonia) is that: canagliflozin ameliorates NLRP3 (i.e., NOD-, LRR-, and pyrin domain-containing protein 3) inflammasome-mediated inflammation through inhibiting NF-κB signaling and upregulating Bif-1 (i.e., Bax-interacting factor 1) (Niu et al., 2022).

This network meta-analysis has two main strengths. First, this network meta-analysis possessed a large sample size: the included RCTs involved a total of 100740 subjects, and the sample size of each drug intervention of our interest ranged from 5806 to 18791. This suggested that this study had a relatively sufficient statistical power to assess each intervention of interest. Second, there was no any potential publication bias observed for all of the 20 cardiovascular and respiratory outcomes assessed in this study. In contrast, this network meta-analysis has two main weaknesses. First, this network meta-analysis only assessed the five gliflozins: canagliflozin, dapagliflozin, empagliflozin, ertugliflozin, and sotagliflozin; whereas this study did not assess the other gliflozins, such as, bexagliflozin, tofogliflozin, and ipragliflozin. This is because the former five gliflozins have been evaluated in large-scale cardiovascular outcome RCTs, whereas the other gliflozins have not until now. Therefore, there is a need for an update for this network meta-analysis when possible. Second, due to the absence of large-scale head-to-head RCTs performing the comparisons among various gliflozins, we only included placebo-controlled trials in this network meta-analysis. It is meaningful to conduct relevant head-to-head trials to confirm our findings.

In conclusion, our network meta-analysis, for the first time, reveals that different gliflozins have different impacts on various cardiovascular and respiratory outcomes. Among the five gliflozins assessed, sotagliflozin may be the best gliflozin for preventing heart failure, myocardial infarction, complete atrioventricular block, and pneumonia; empagliflozin may be the best gliflozin for preventing acute heart failure, acute respiratory failure, and hypertensive crisis; and dapagliflozin may be the best gliflozin for preventing asthma, and hypertensive emergency. However, further research, including head-to-head trials, is needed to confirm and expand upon these findings.

## Abbreviations

CI, confidence interval; COPD, chronic obstructive pulmonary disease; LRTI, lower respiratory tract infection; SGLT2, sodium-glucose cotransporter-2; RR, risk ratio; SUCRA, surface under the cumulative ranking curve; RCTs, randomized controlled trials; PRISMA, Preferred Reporting Items for Systematic Reviews and Meta-Analyses; SAEs, serious adverse events.
