# Supplementary material for: Relative efficacy of five SGLT2 inhibitors: a network meta-analysis of 20 cardiovascular and respiratory outcomes
Source: Front Pharmacol. 2024 Jun 12;15:1419729. doi: 10.3389/fphar.2024.1419729 (PMC11199404; doi:10.3389/fphar.2024.1419729)
Supplement: Supplementary file 2 [file Image1.PDF]

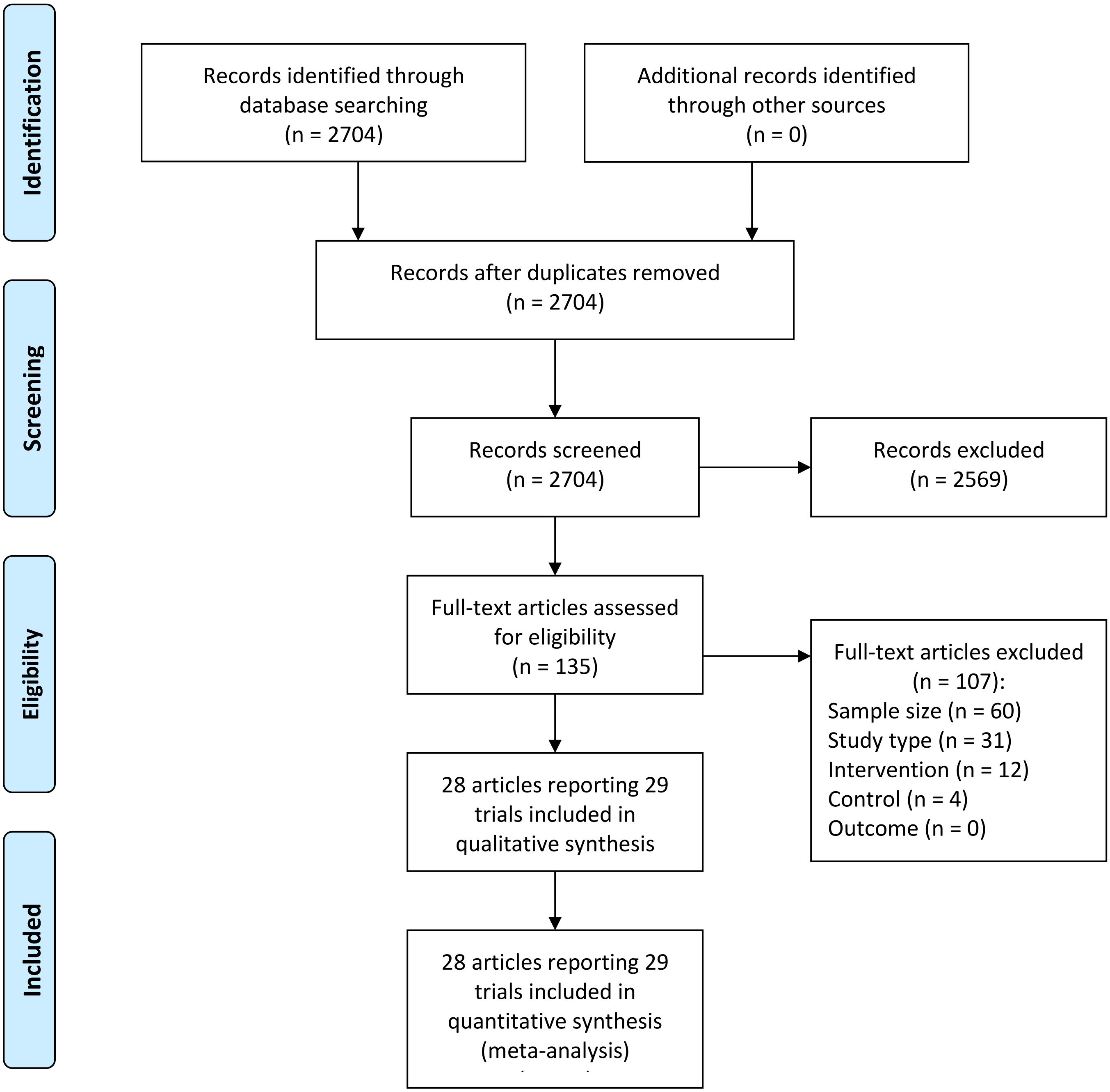

Figure S1 PRISMA flow diagram

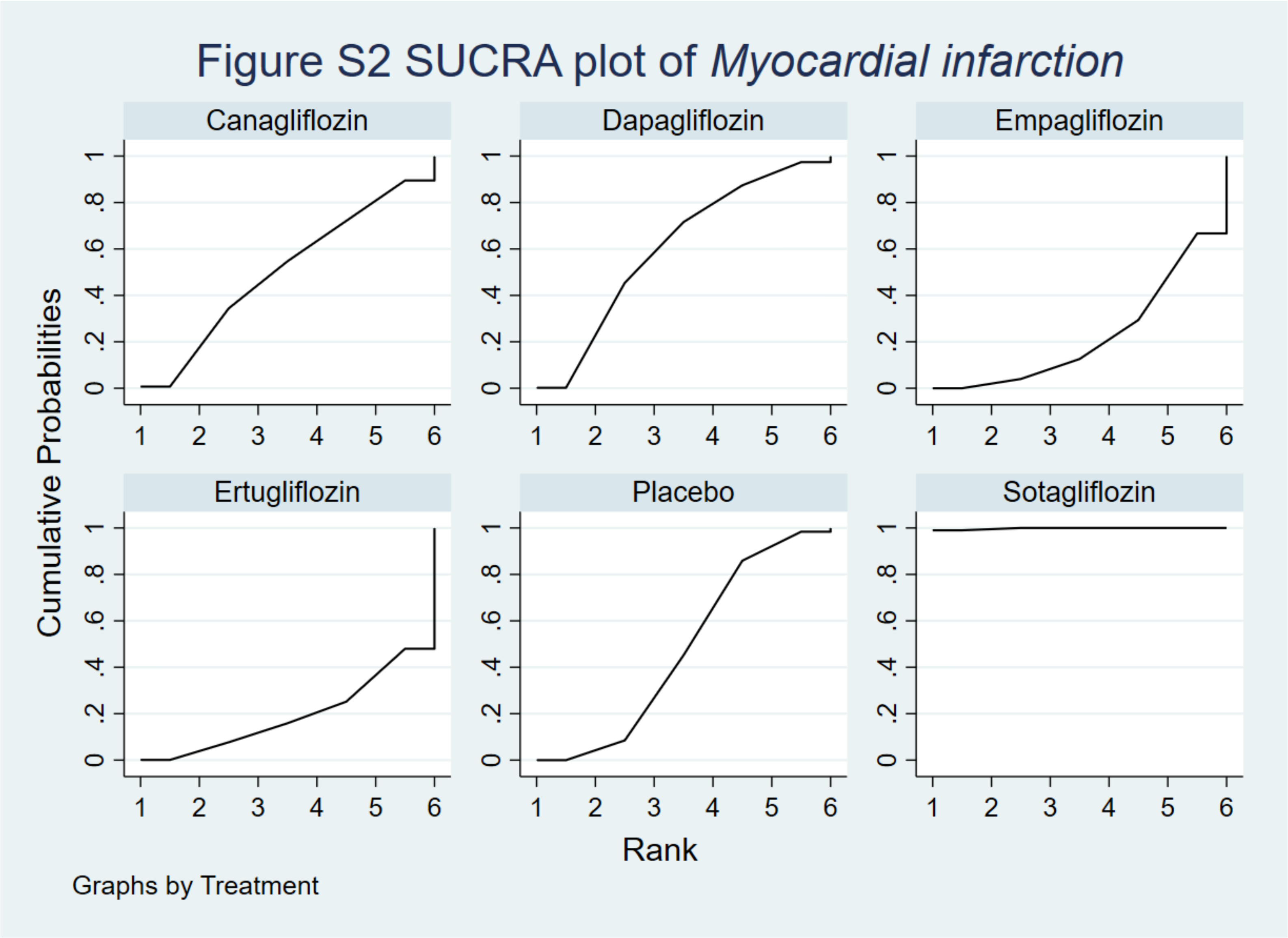
Figure S2 SUCRA plot of *Myocardial infarction*
Canagliflozin
Dapagliflozin
Empagliflozin
Ertugliflozin
Placebo
Sotagliflozin
Cumulative Probabilities
0
.2
.4
.6
.8
1
1
2
3
4
5
6
Rank
Graphs by Treatment

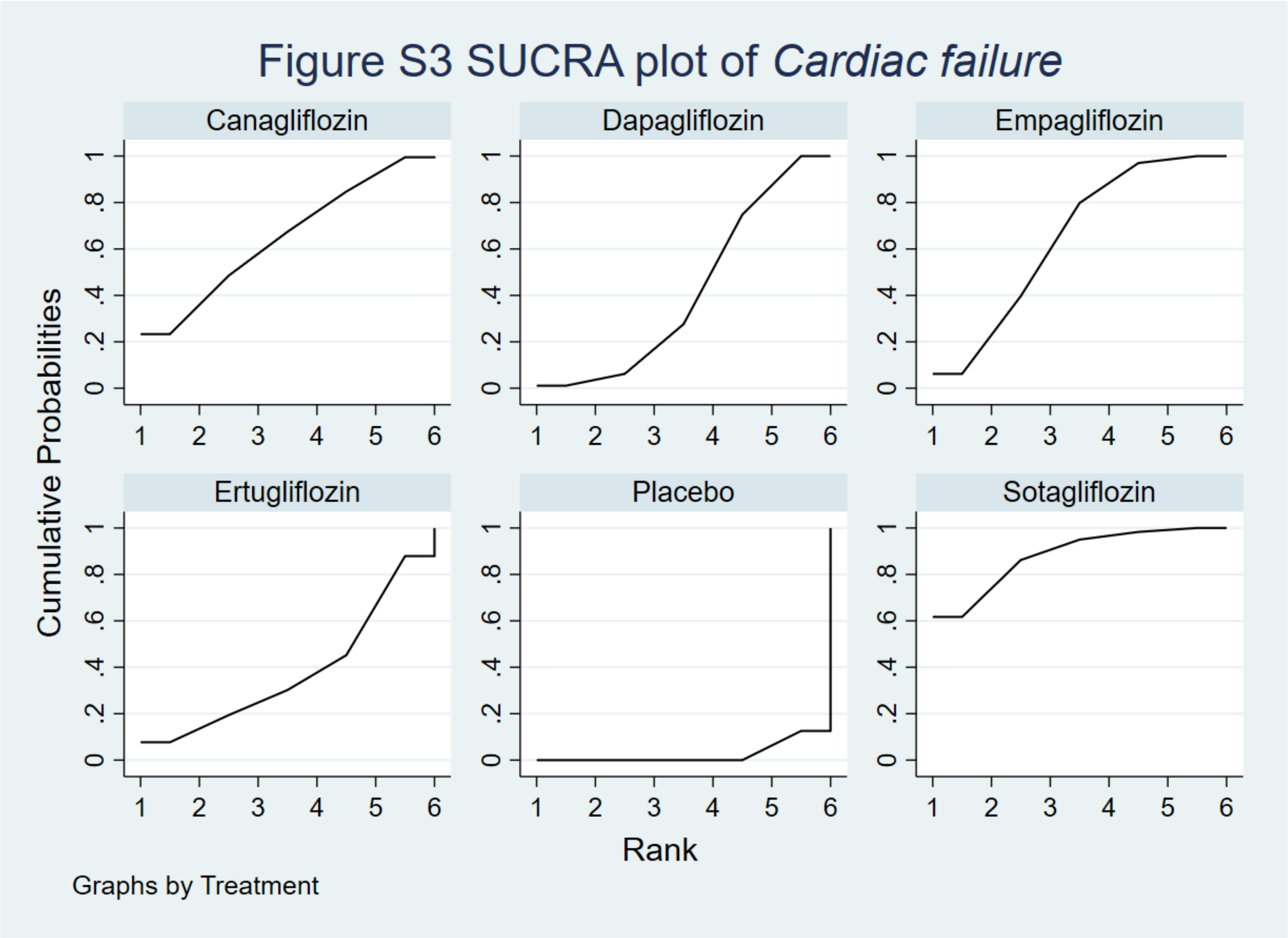
Figure S3 SUCRA plot of *Cardiac failure*
Canagliflozin
Dapagliflozin
Empagliflozin
Ertugliflozin
Placebo
Sotagliflozin
Cumulative Probabilities
0
.2
.4
.6
.8
1
1 2 3 4 5 6
Rank
Graphs by Treatment

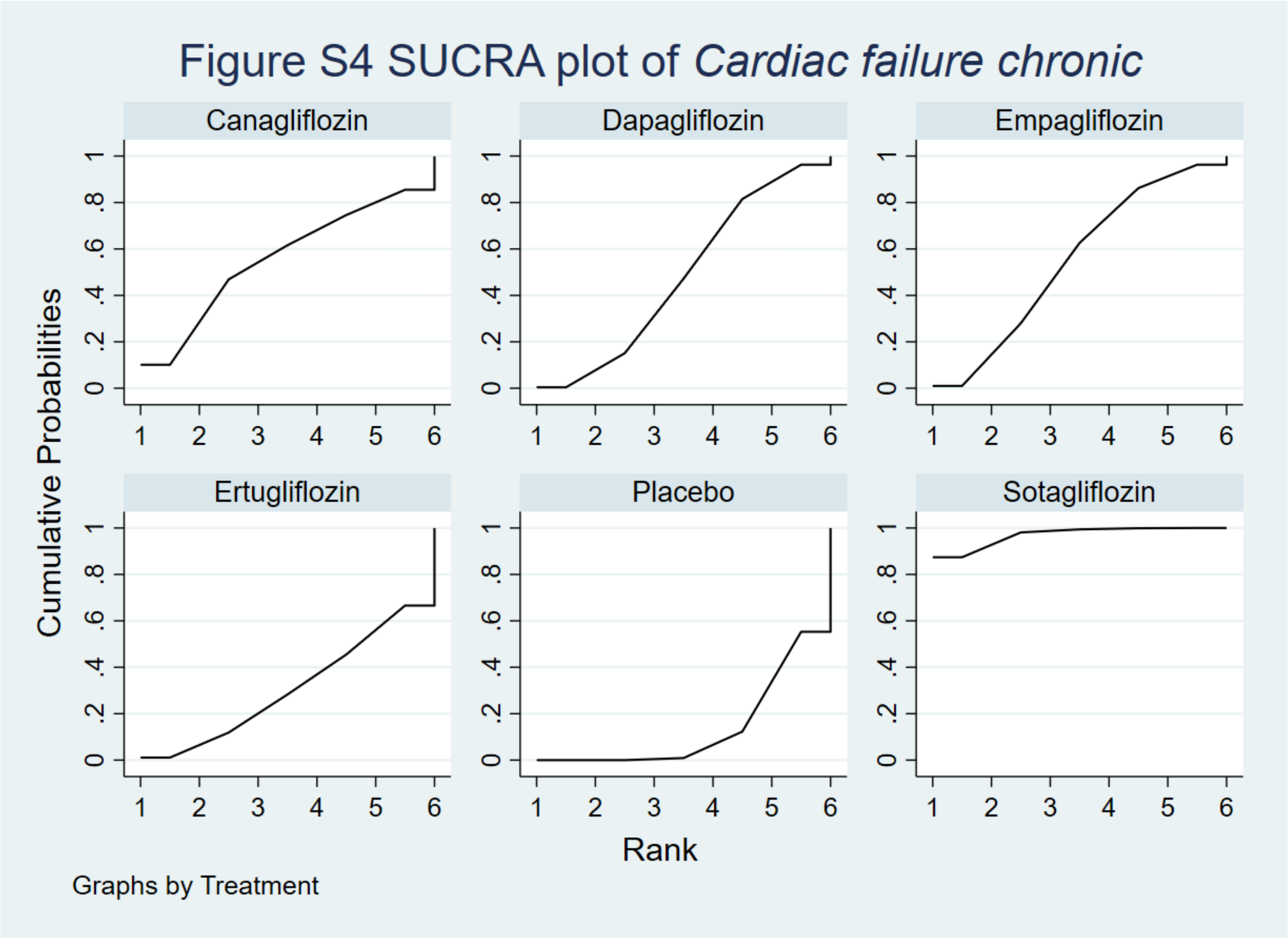

Figure S4 SUCRA plot of *Cardiac failure chronic*
Canagliflozin
Dapagliflozin
Empagliflozin
Ertugliflozin
Placebo
Sotagliflozin
Cumulative Probabilities
0
.2
.4
.6
.8
1
1 2 3 4 5 6
Rank
Graphs by Treatment

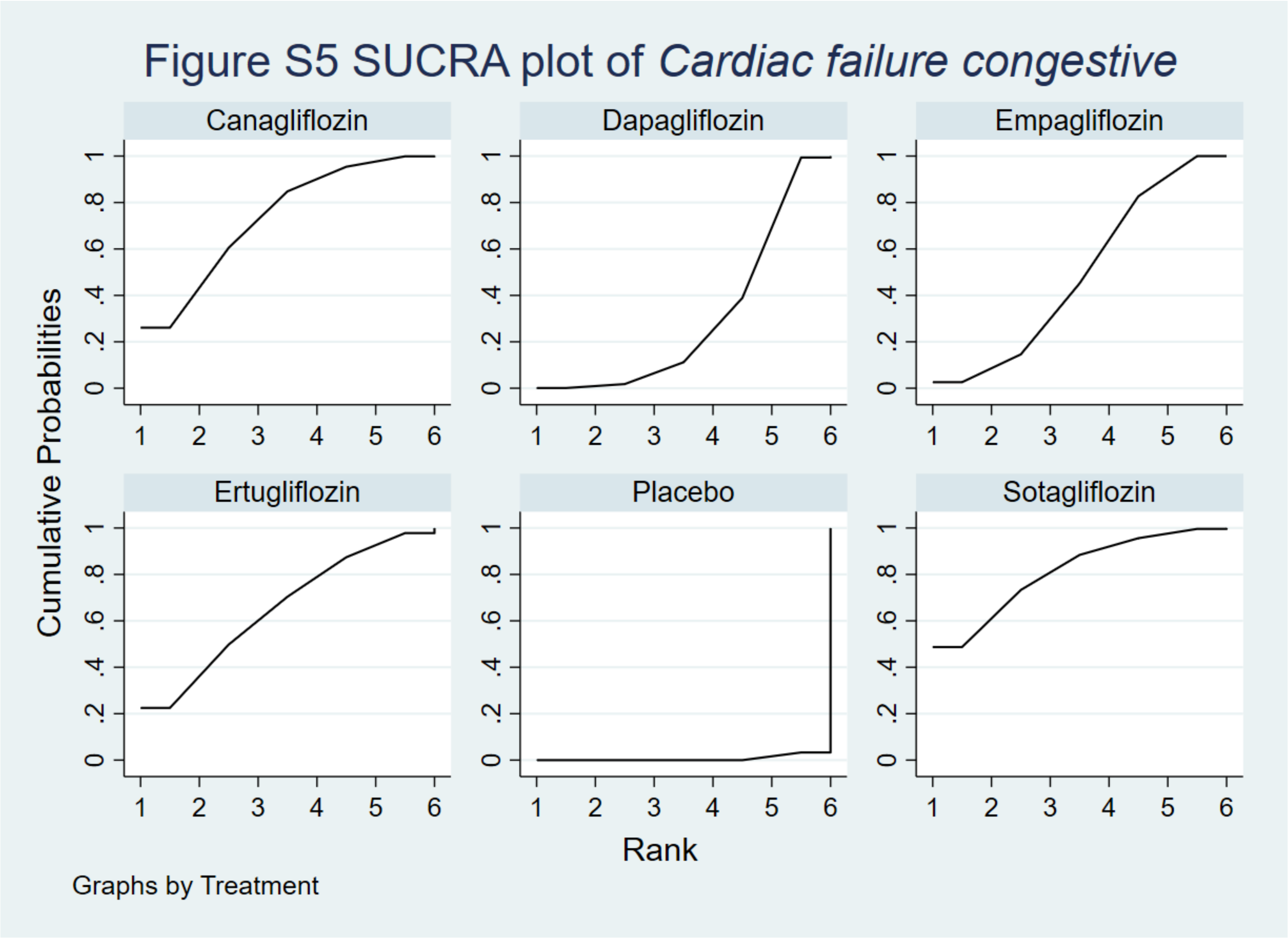
Figure S5 SUCRA plot of *Cardiac failure congestive*
Canagliflozin
Dapagliflozin
Empagliflozin
Ertugliflozin
Placebo
Sotagliflozin
Cumulative Probabilities
0
.2
.4
.6
.8
1
1
2
3
4
5
6
Rank
Graphs by Treatment

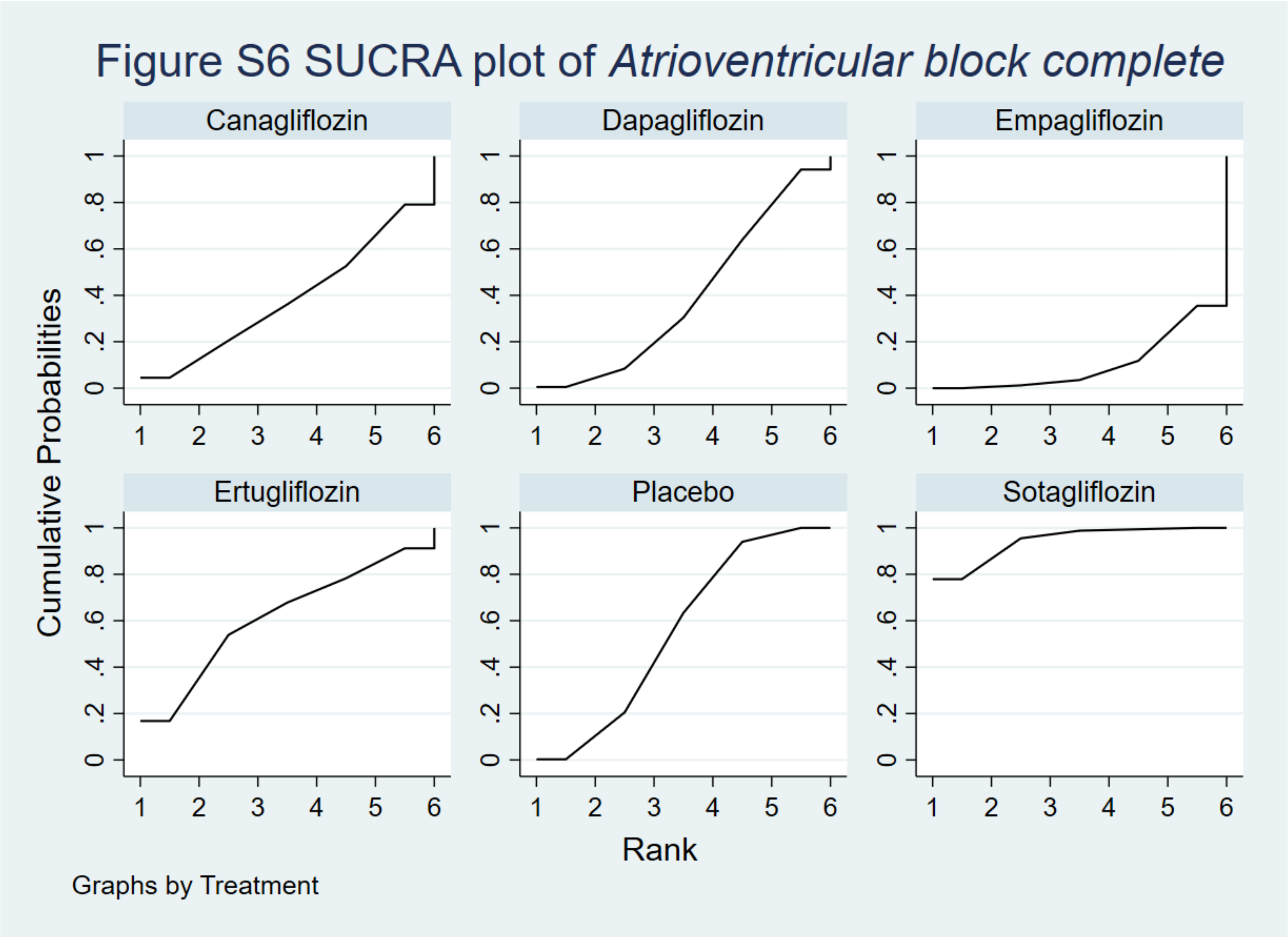
Figure S6 SUCRA plot of *Atrioventricular block complete*
Canagliflozin
Dapagliflozin
Empagliflozin
Ertugliflozin
Placebo
Sotagliflozin
Cumulative Probabilities
Rank
Graphs by Treatment

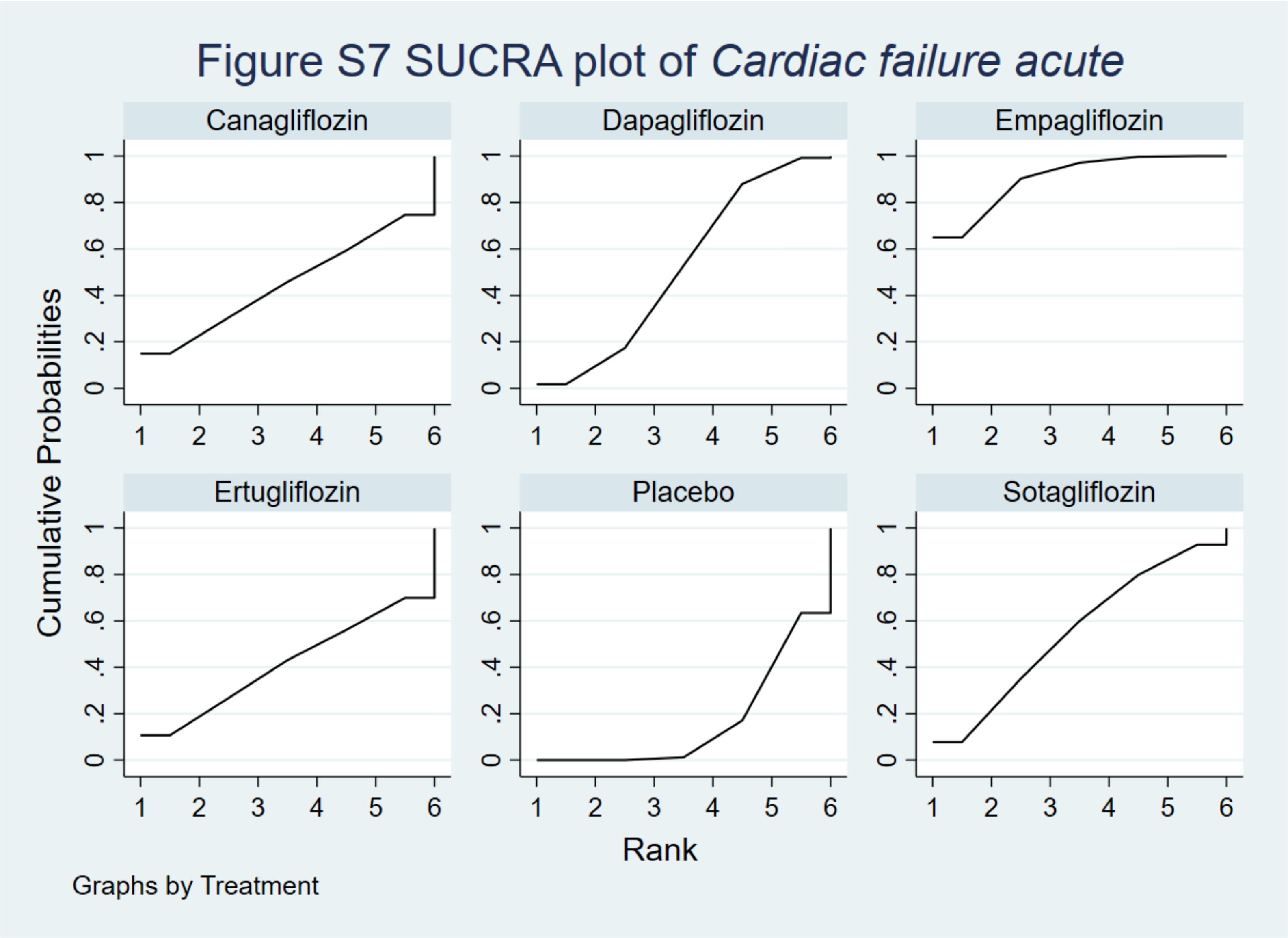
Figure S7 SUCRA plot of *Cardiac failure acute*
Canagliflozin
Dapagliflozin
Empagliflozin
Ertugliflozin
Placebo
Sotagliflozin
Cumulative Probabilities
0
.2
.4
.6
.8
1
1
2
3
4
5
6
Rank
Graphs by Treatment

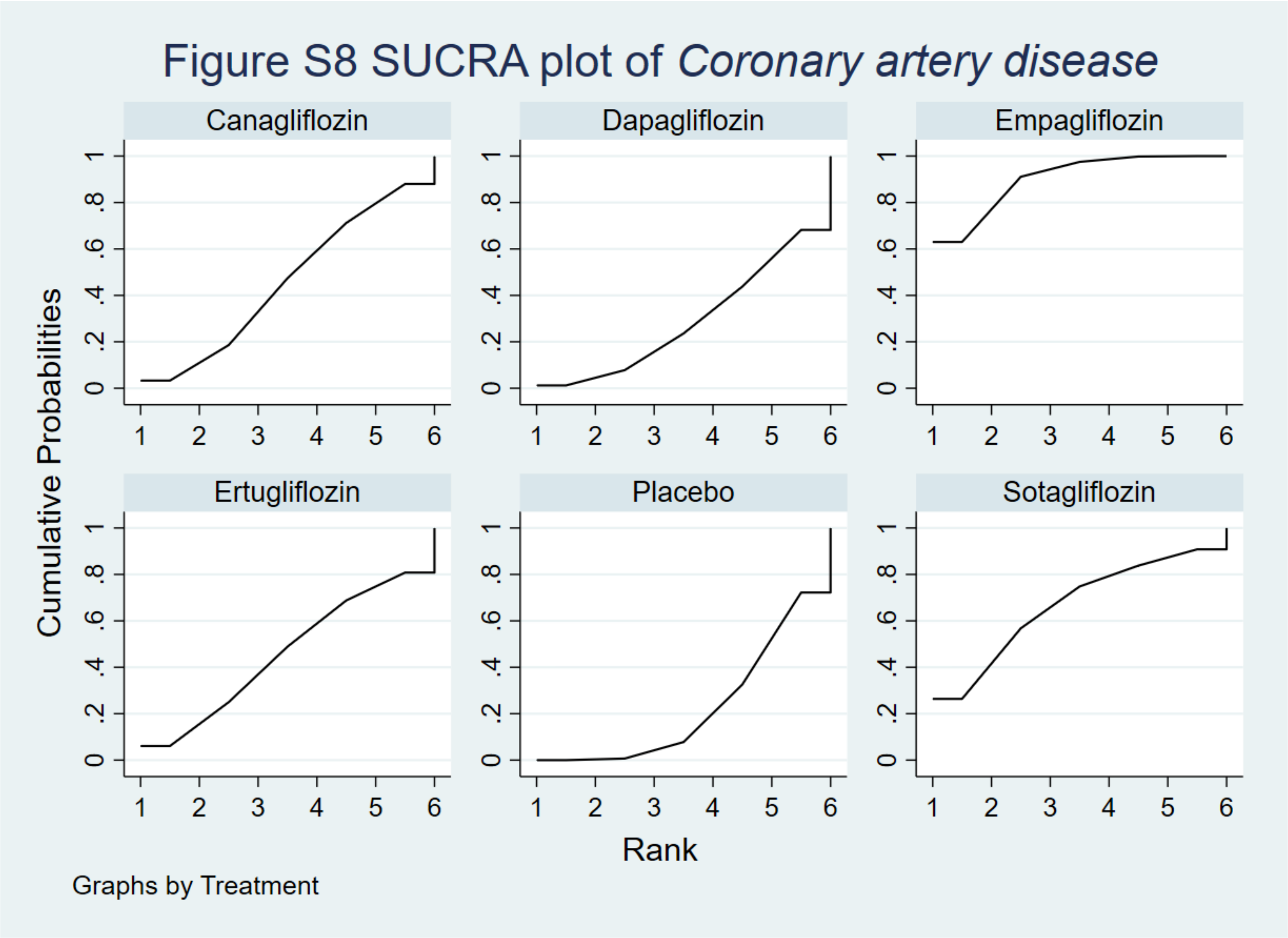
Figure S8 SUCRA plot of *Coronary artery disease*
Canagliflozin
Dapagliflozin
Empagliflozin
Ertugliflozin
Placebo
Sotagliflozin
Cumulative Probabilities
0
.2
.4
.6
.8
1
1
2
3
4
5
6
Rank
Graphs by Treatment

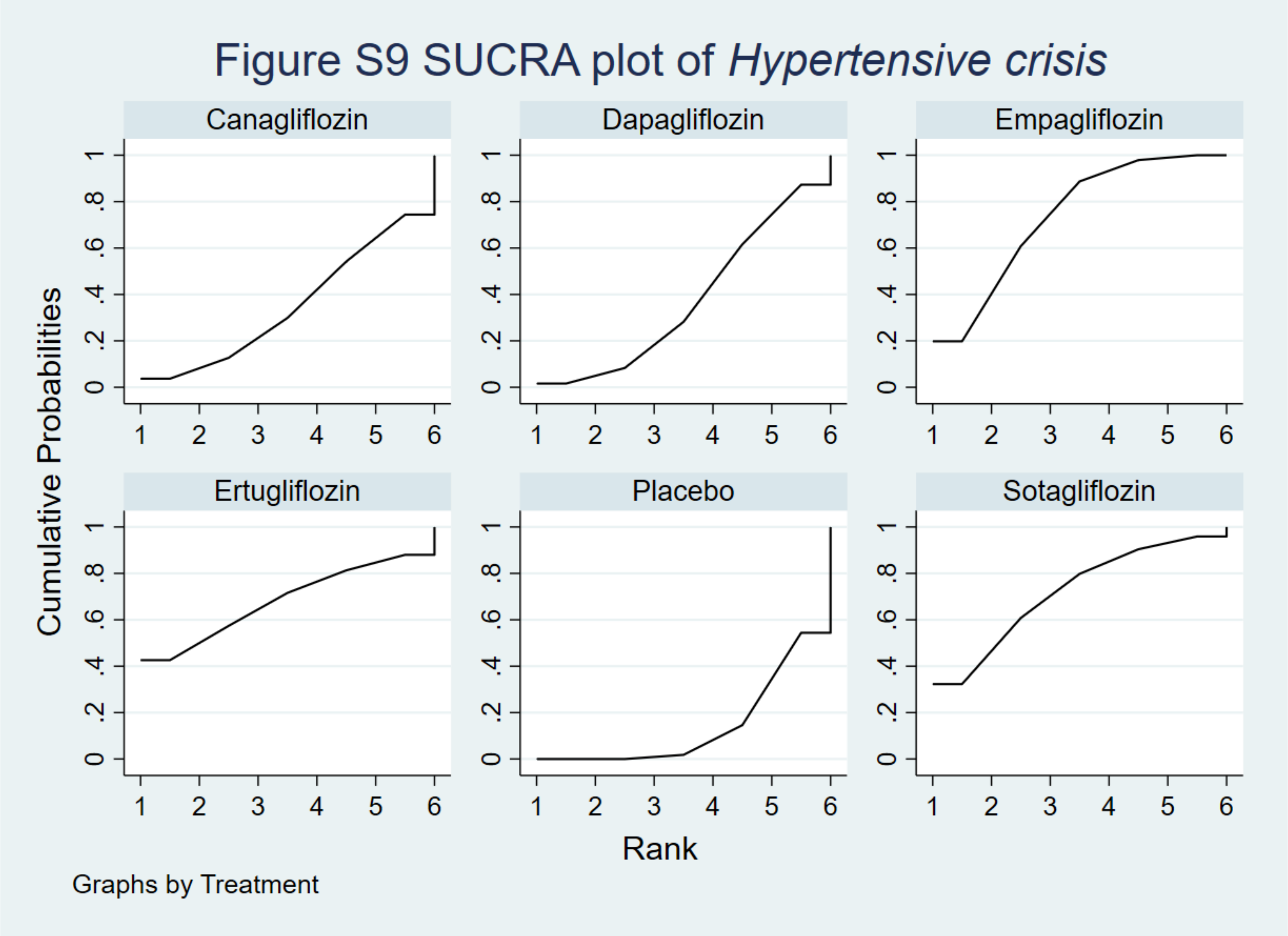
Figure S9 SUCRA plot of *Hypertensive crisis*
Canagliflozin
Dapagliflozin
Empagliflozin
Ertugliflozin
Placebo
Sotagliflozin
Cumulative Probabilities
Rank
Graphs by Treatment

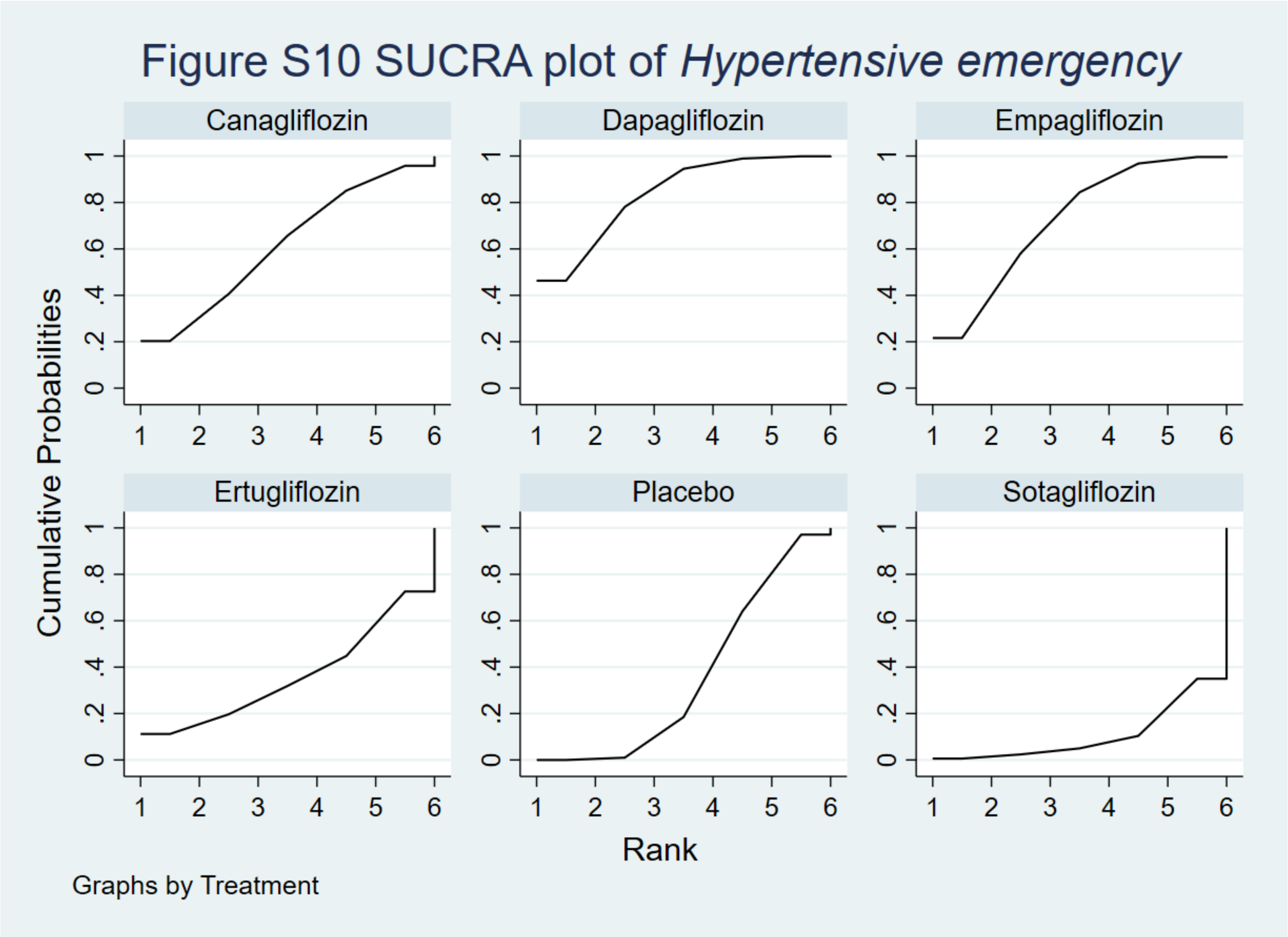
Figure S10 SUCRA plot of *Hypertensive emergency*
Canagliflozin
Dapagliflozin
Empagliflozin
Ertugliflozin
Placebo
Sotagliflozin
Cumulative Probabilities
0
.2
.4
.6
.8
1
1
2
3
4
5
6
Rank
Graphs by Treatment

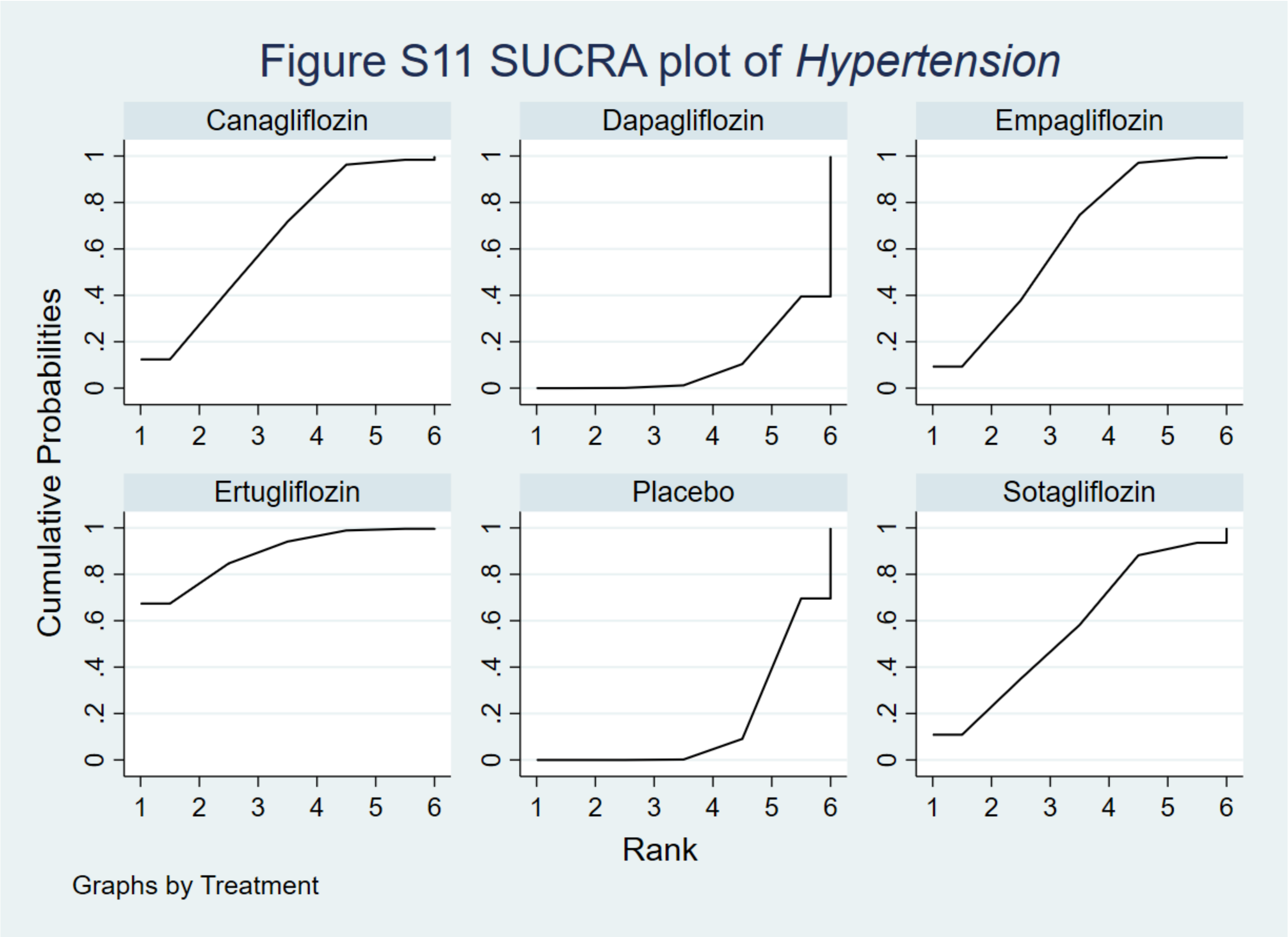
Figure S11 SUCRA plot of *Hypertension*
Canagliflozin
Dapagliflozin
Empagliflozin
Ertugliflozin
Placebo
Sotagliflozin
Cumulative Probabilities
0
.2
.4
.6
.8
1
1
2
3
4
5
6
Rank
Graphs by Treatment

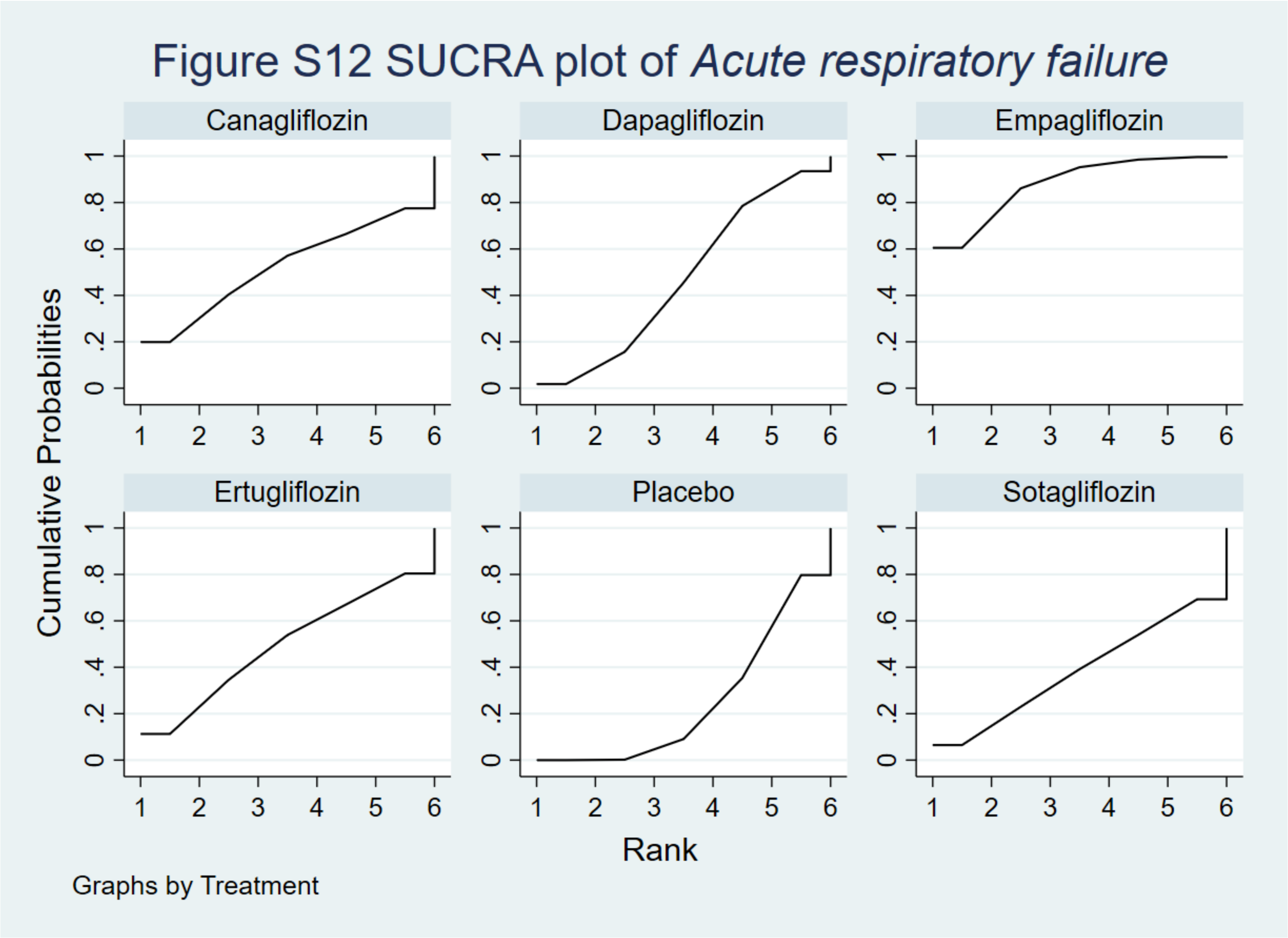

Figure S12 SUCRA plot of *Acute respiratory failure*

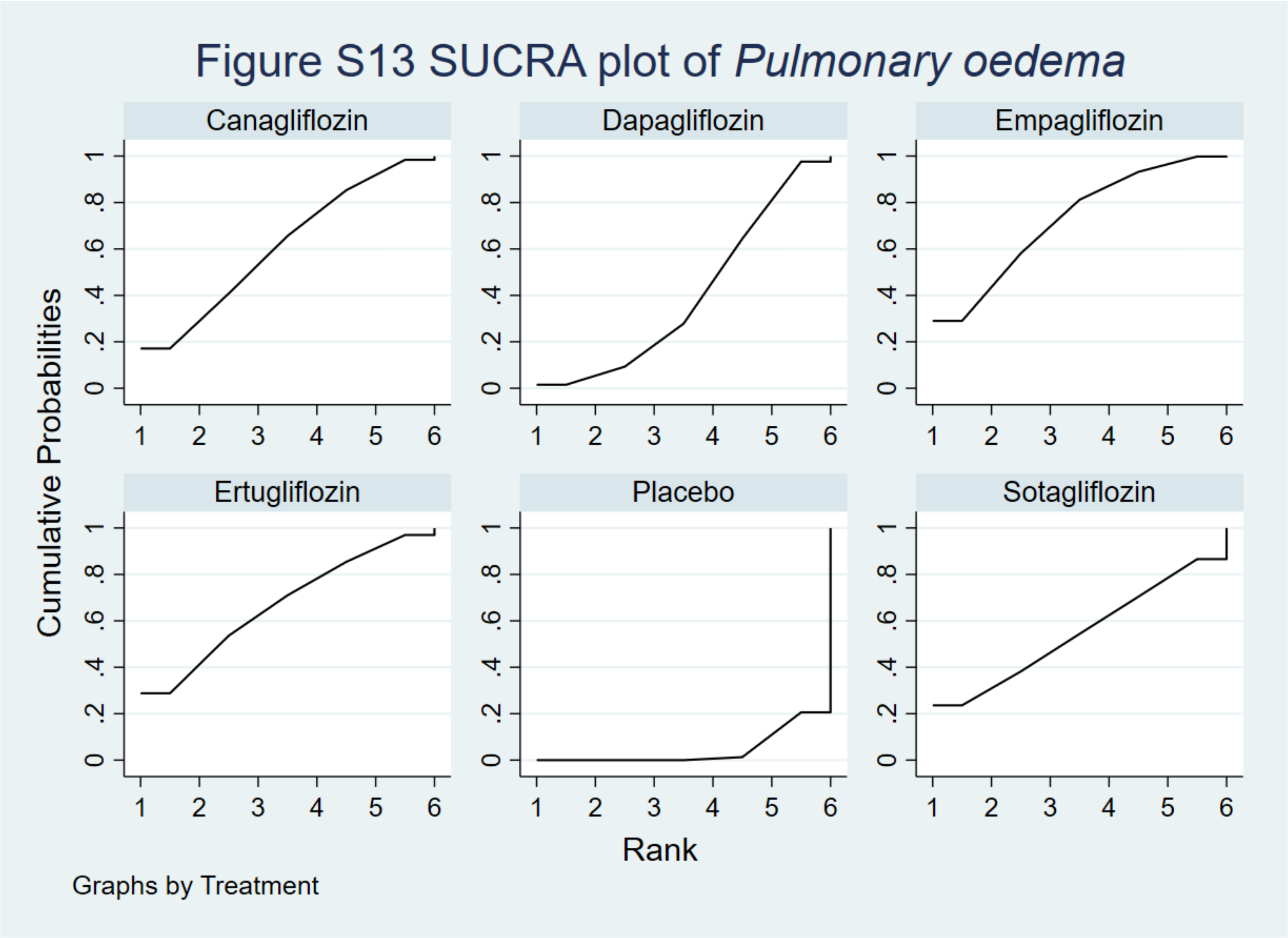
Figure S13 SUCRA plot of *Pulmonary oedema*
Canagliflozin
Dapagliflozin
Empagliflozin
Ertugliflozin
Placebo
Sotagliflozin
Cumulative Probabilities
0
.2
.4
.6
.8
1
1
2
3
4
5
6
Rank
Graphs by Treatment

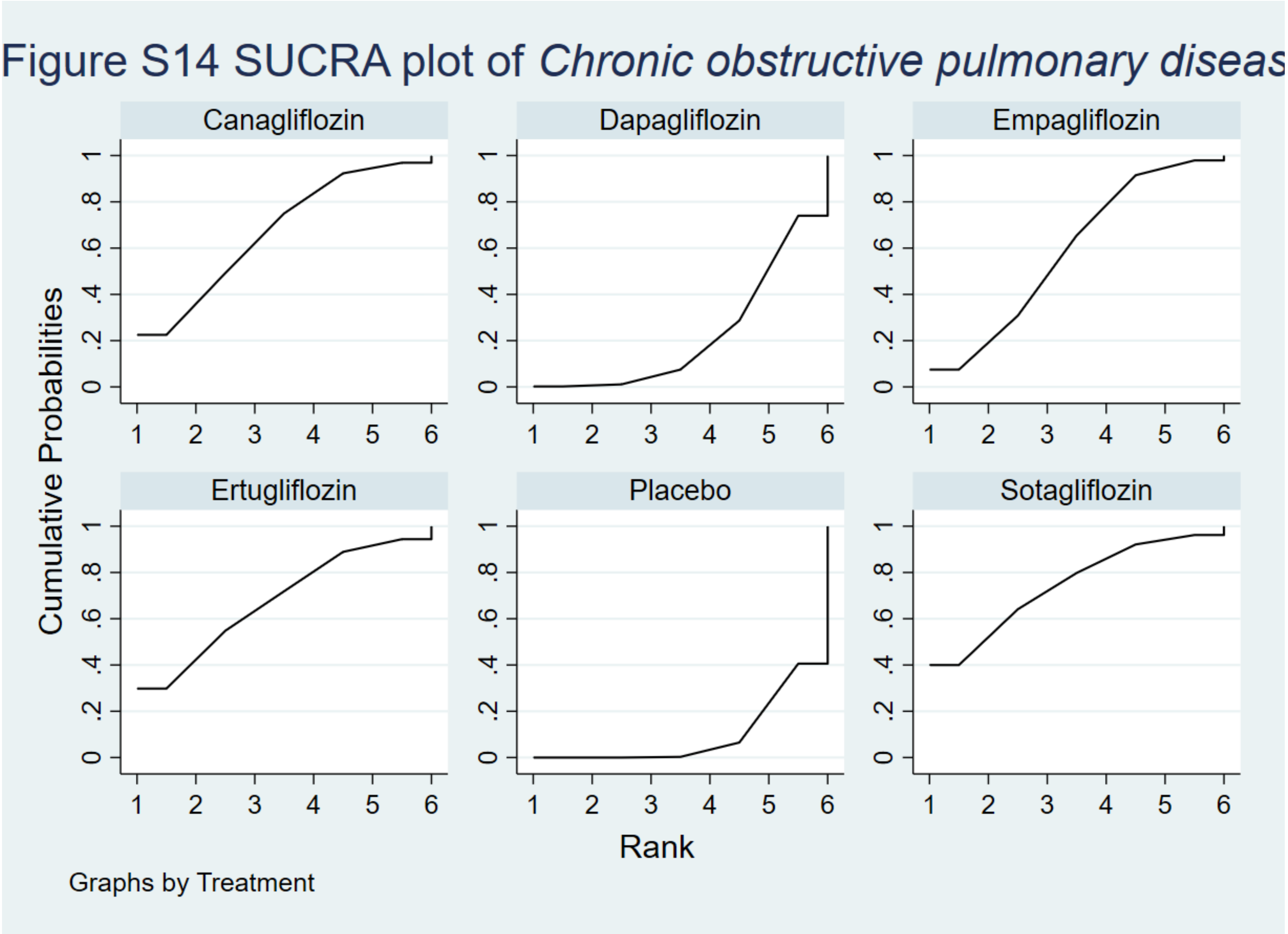
Figure S14 SUCRA plot of Chronic obstructive pulmonary diseas
Canagliflozin
Dapagliflozin
Empagliflozin
Ertugliflozin
Placebo
Sotagliflozin
Cumulative Probabilities
0
.2
.4
.6
.8
1
1
2
3
4
5
6
Rank
Graphs by Treatment

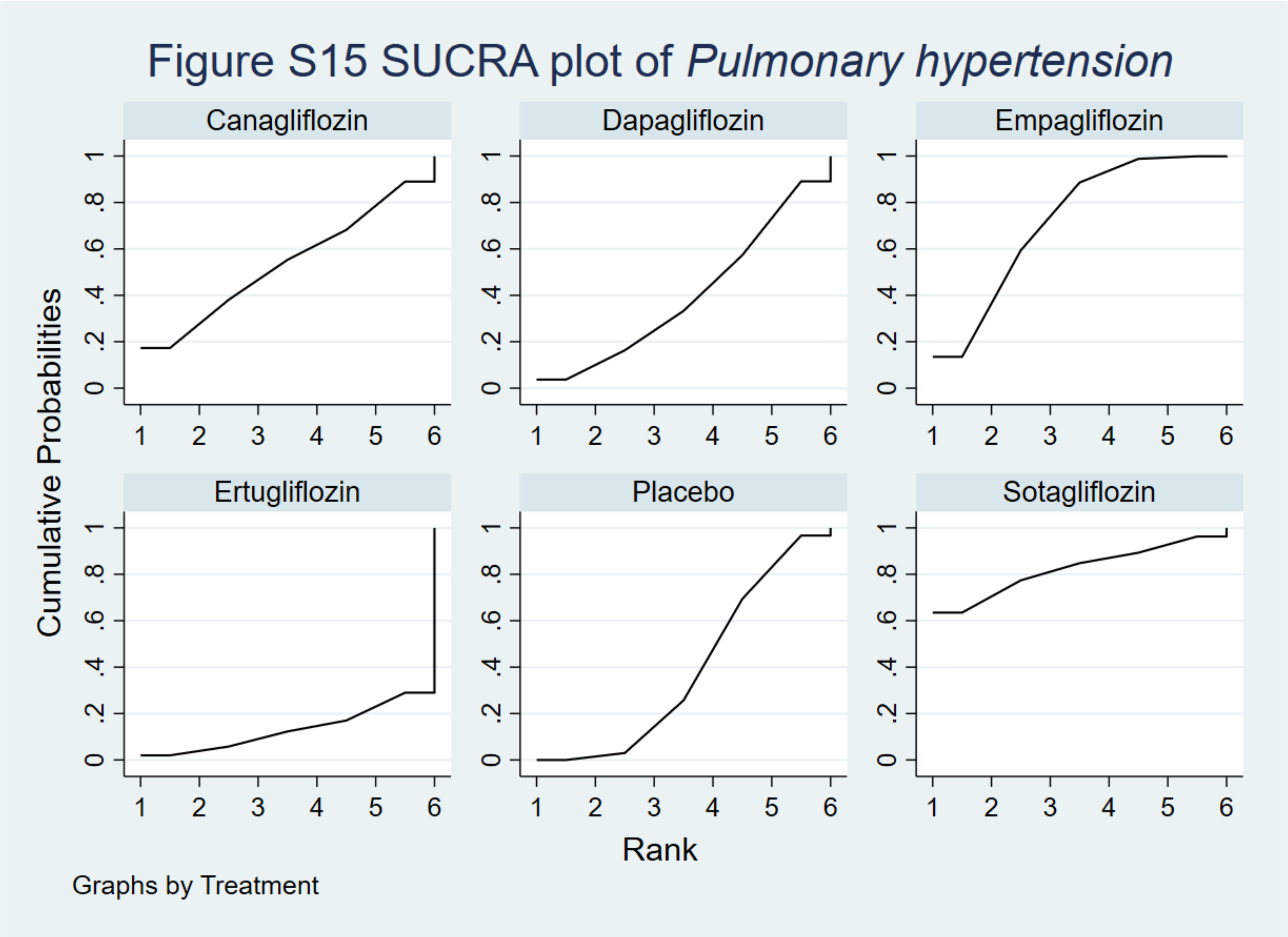

Figure S15 SUCRA plot of *Pulmonary hypertension*
Canagliflozin
Dapagliflozin
Empagliflozin
Ertugliflozin
Placebo
Sotagliflozin
Cumulative Probabilities
0 .2 .4 .6 .8 1
1 2 3 4 5 6
Rank
Graphs by Treatment

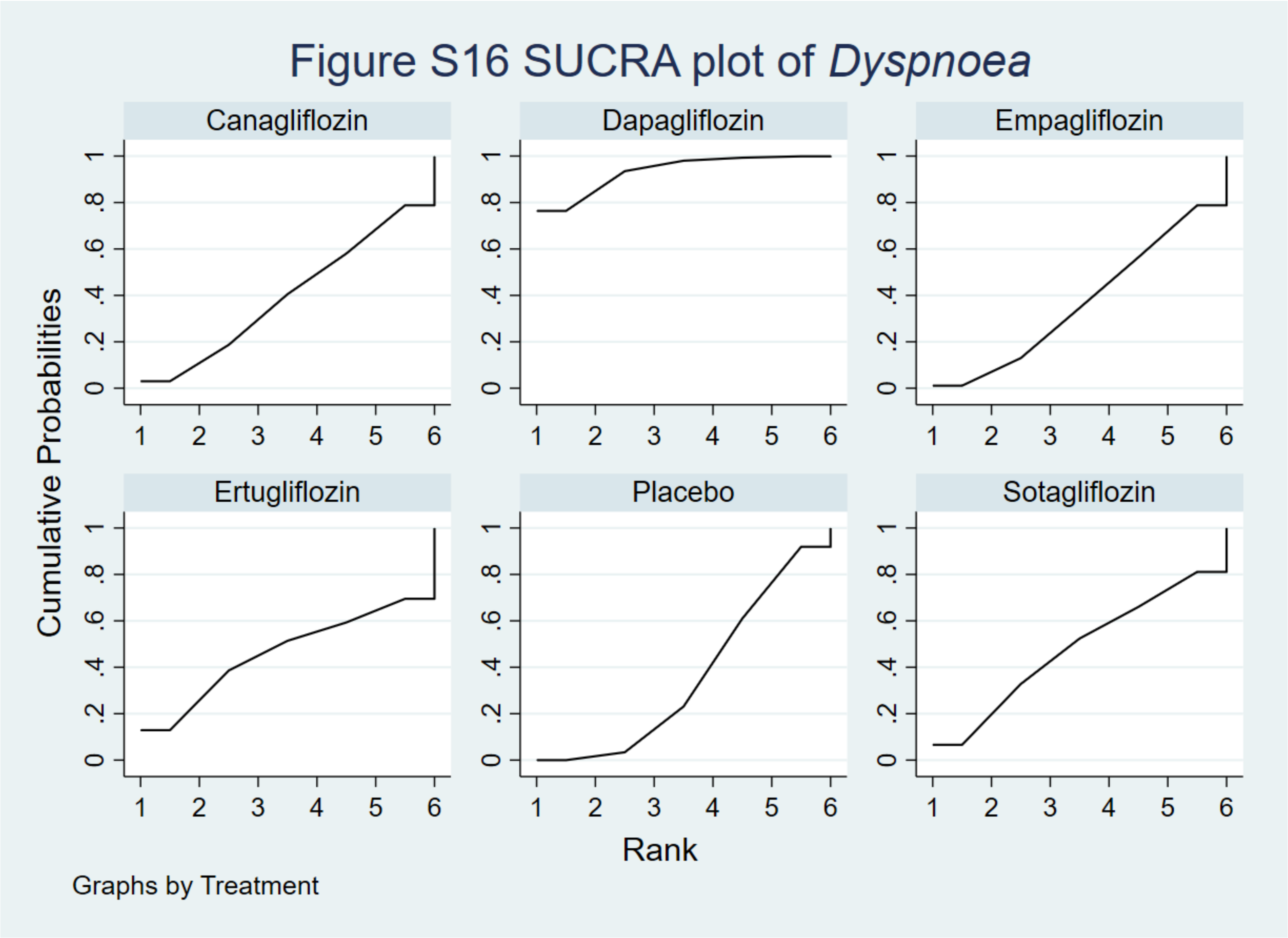

Figure S16 SUCRA plot of *Dyspnoea*
Canagliflozin
Dapagliflozin
Empagliflozin
Ertugliflozin
Placebo
Sotagliflozin
Cumulative Probabilities
0
.2
.4
.6
.8
1
1
2
3
4
5
6
Rank
Graphs by Treatment

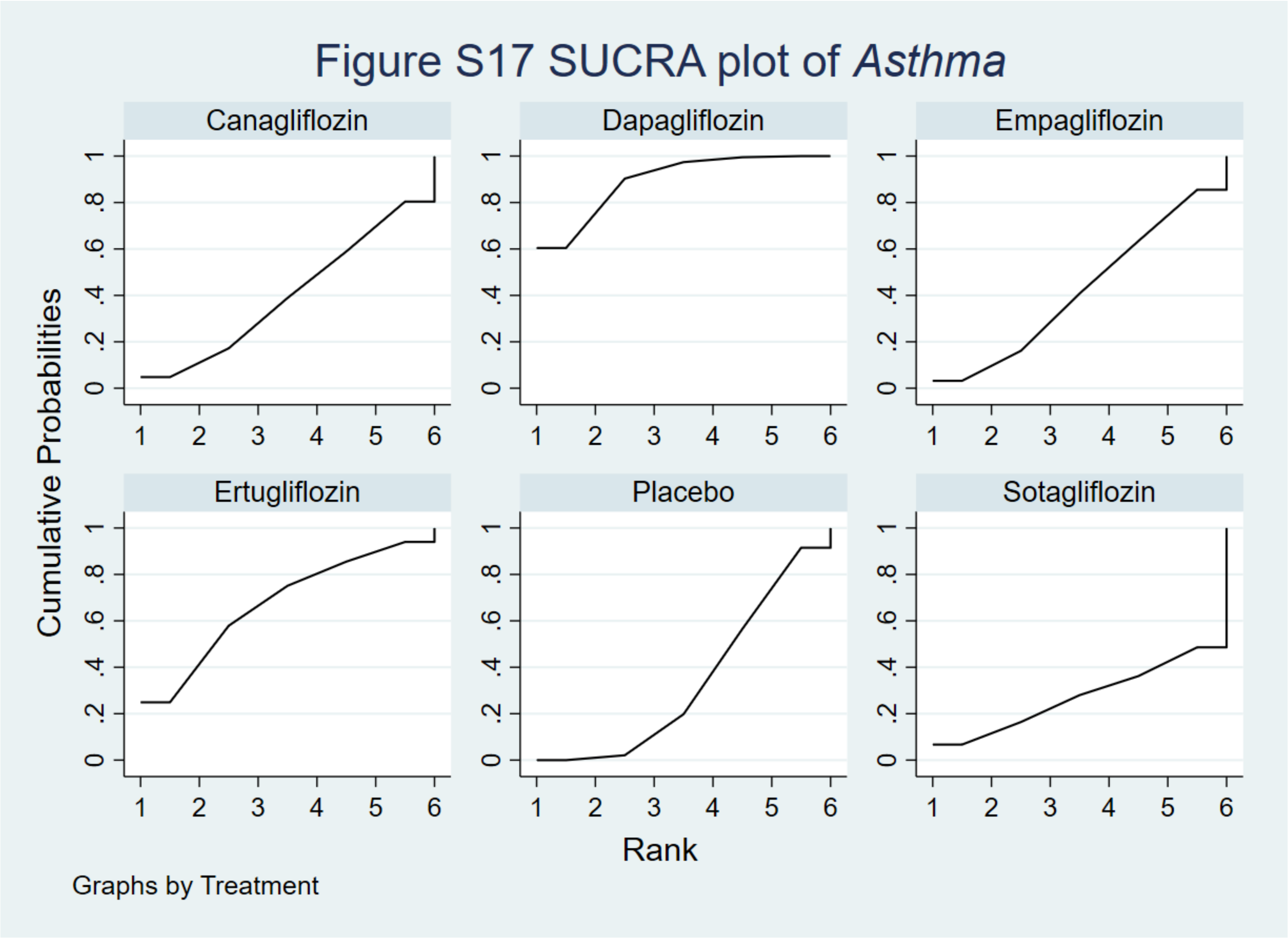
Figure S17 SUCRA plot of *Asthma*
Canagliflozin
Dapagliflozin
Empagliflozin
Ertugliflozin
Placebo
Sotagliflozin
Cumulative Probabilities
0
.2
.4
.6
.8
1
1
2
3
4
5
6
Rank
Graphs by Treatment

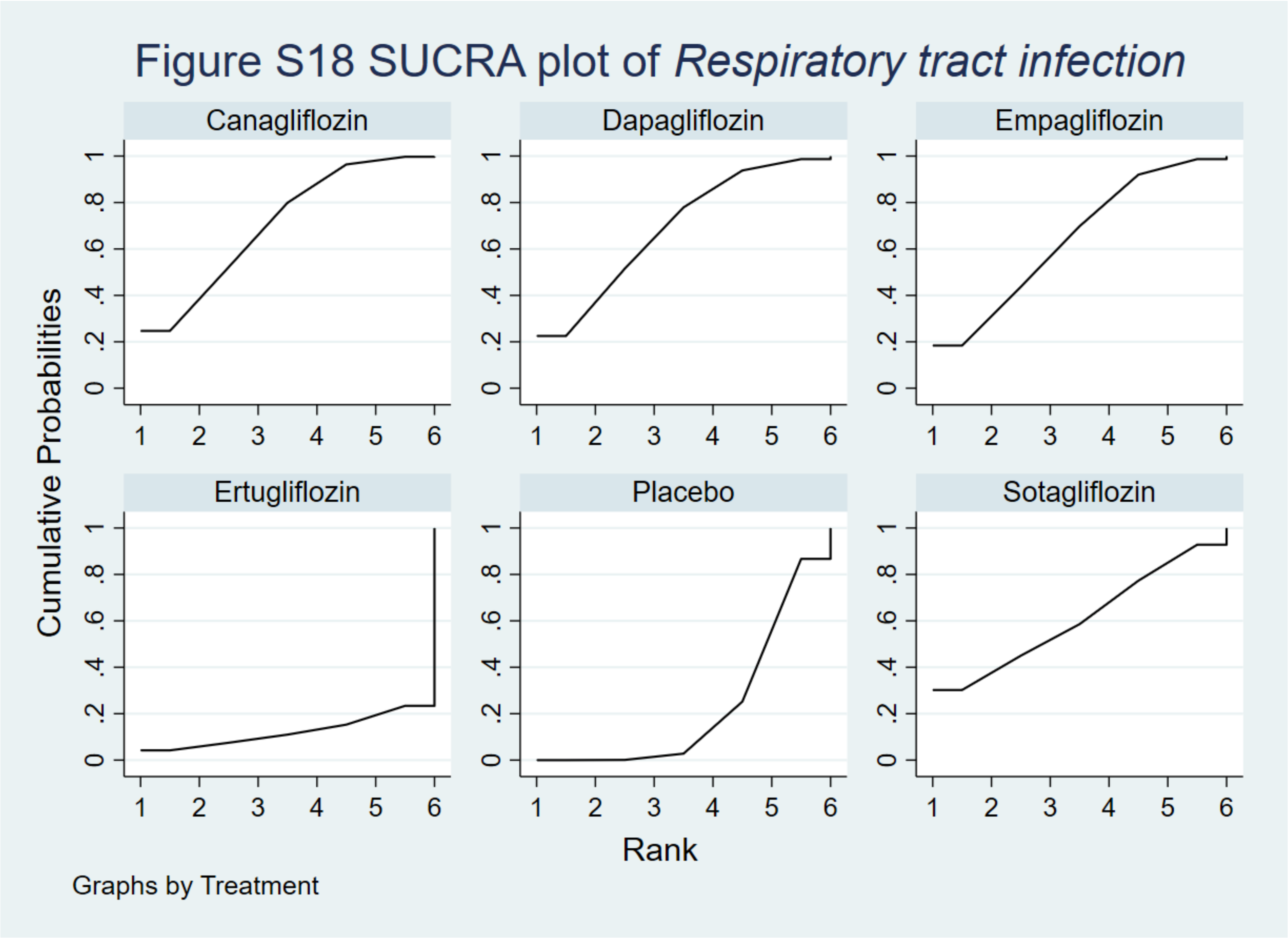
Figure S18 SUCRA plot of *Respiratory tract infection*
Canagliflozin
Dapagliflozin
Empagliflozin
Ertugliflozin
Placebo
Sotagliflozin
Cumulative Probabilities
0
.2
.4
.6
.8
1
1
2
3
4
5
6
Rank
Graphs by Treatment

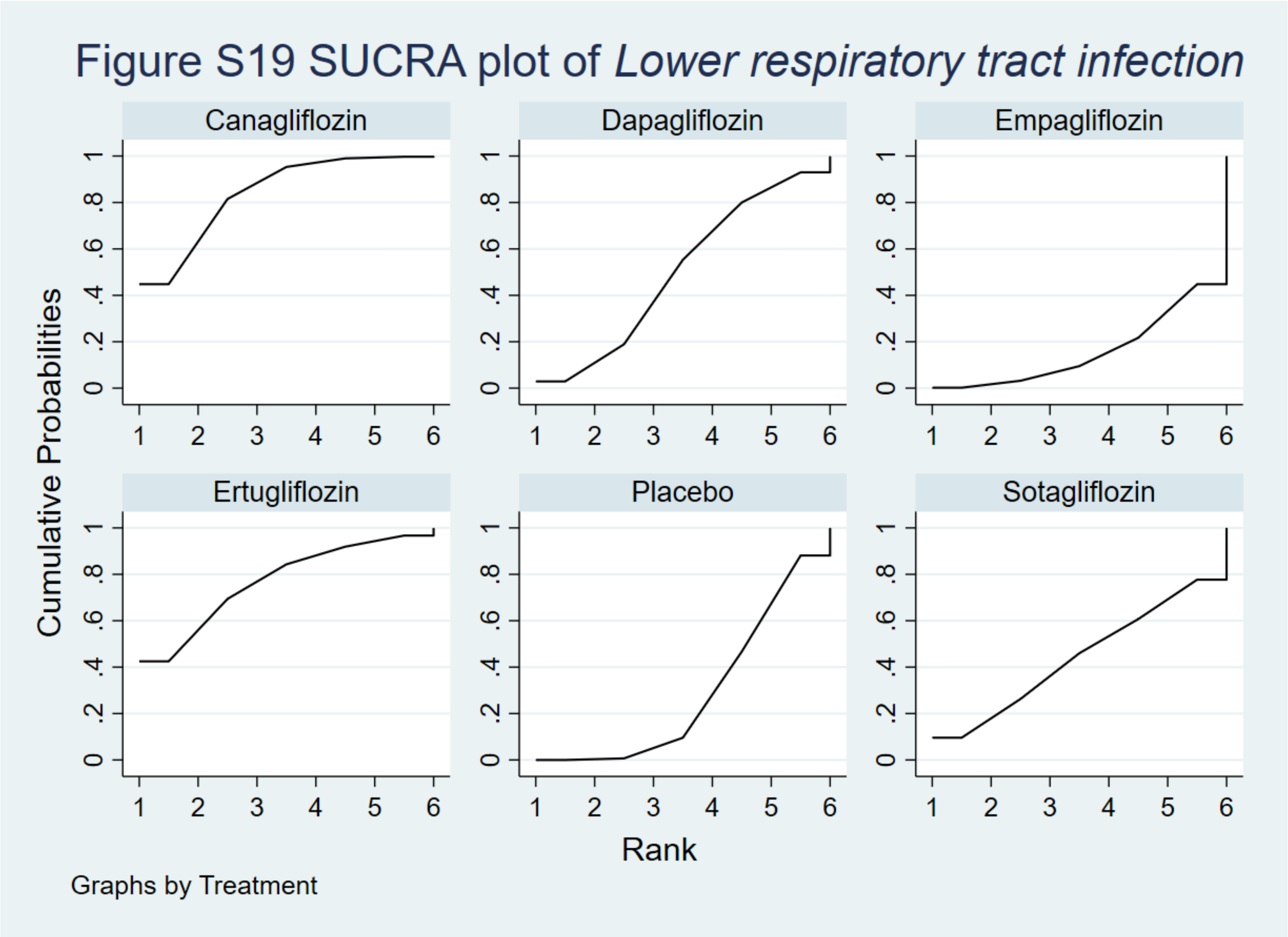
Figure S19 SUCRA plot of *Lower respiratory tract infection*
Canagliflozin
Dapagliflozin
Empagliflozin
Ertugliflozin
Placebo
Sotagliflozin
Cumulative Probabilities
0
.2
.4
.6
.8
1
1
2
3
4
5
6
Rank
Graphs by Treatment

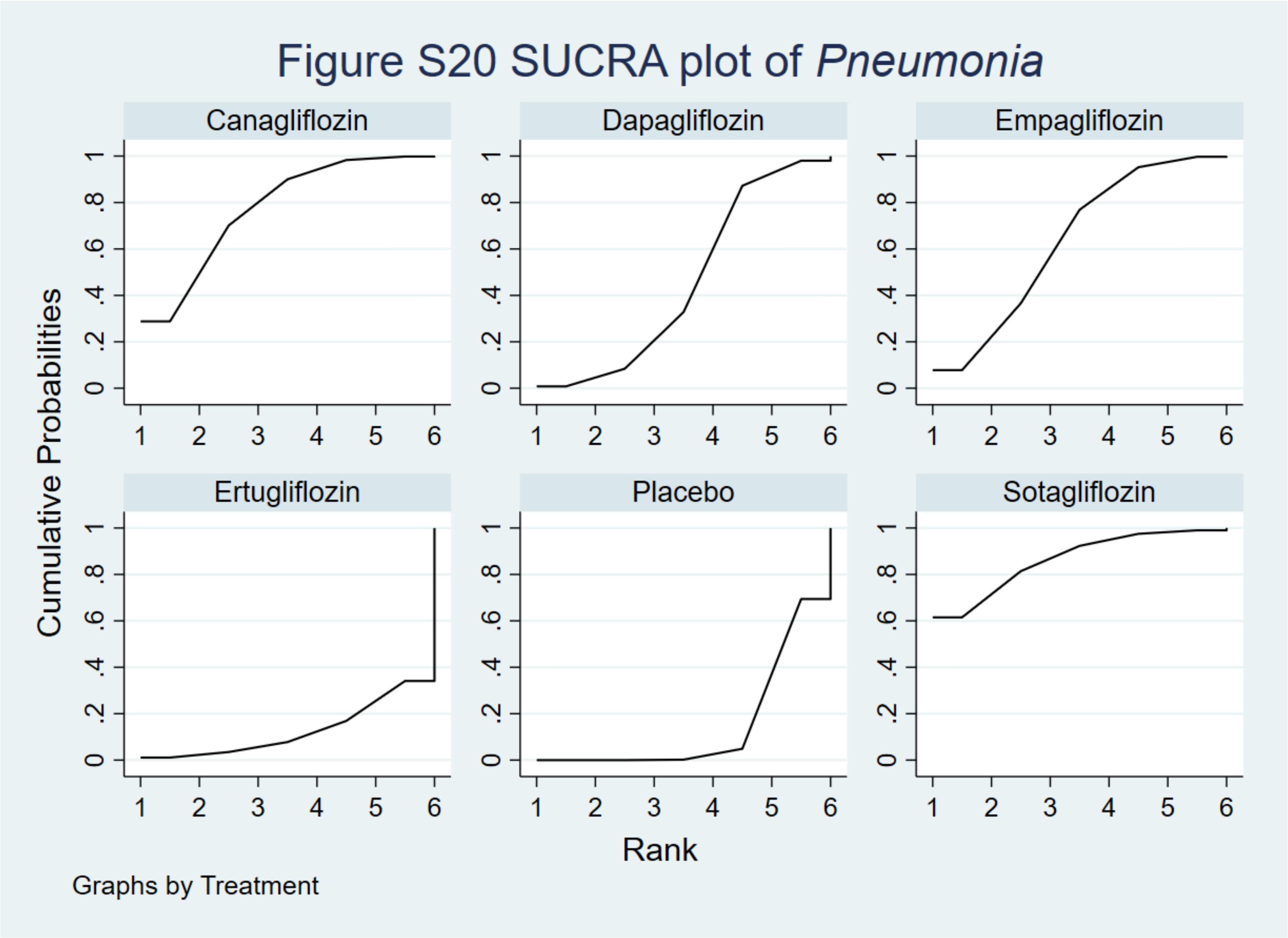
Figure S20 SUCRA plot of Pneumonia
Canagliflozin
Dapagliflozin
Empagliflozin
Ertugliflozin
Placebo
Sotagliflozin
Cumulative Probabilities
0
.2
.4
.6
.8
1
1
2
3
4
5
6
Rank
Graphs by Treatment

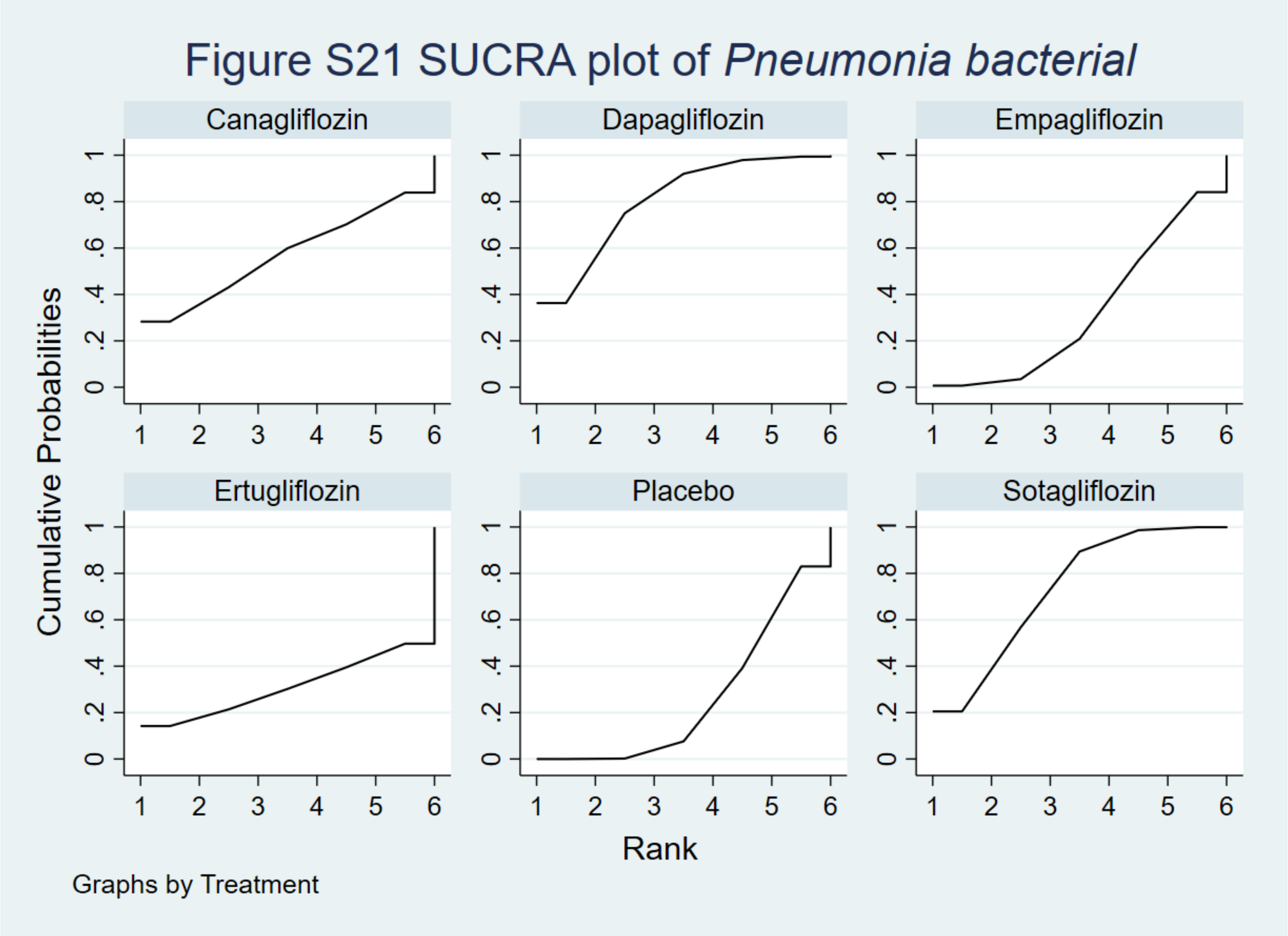
Figure S21 SUCRA plot of *Pneumonia bacterial*
Canagliflozin
Dapagliflozin
Empagliflozin
Ertugliflozin
Placebo
Sotagliflozin
Cumulative Probabilities
0 .2 .4 .6 .8 1
1 2 3 4 5 6
Rank
Graphs by Treatment

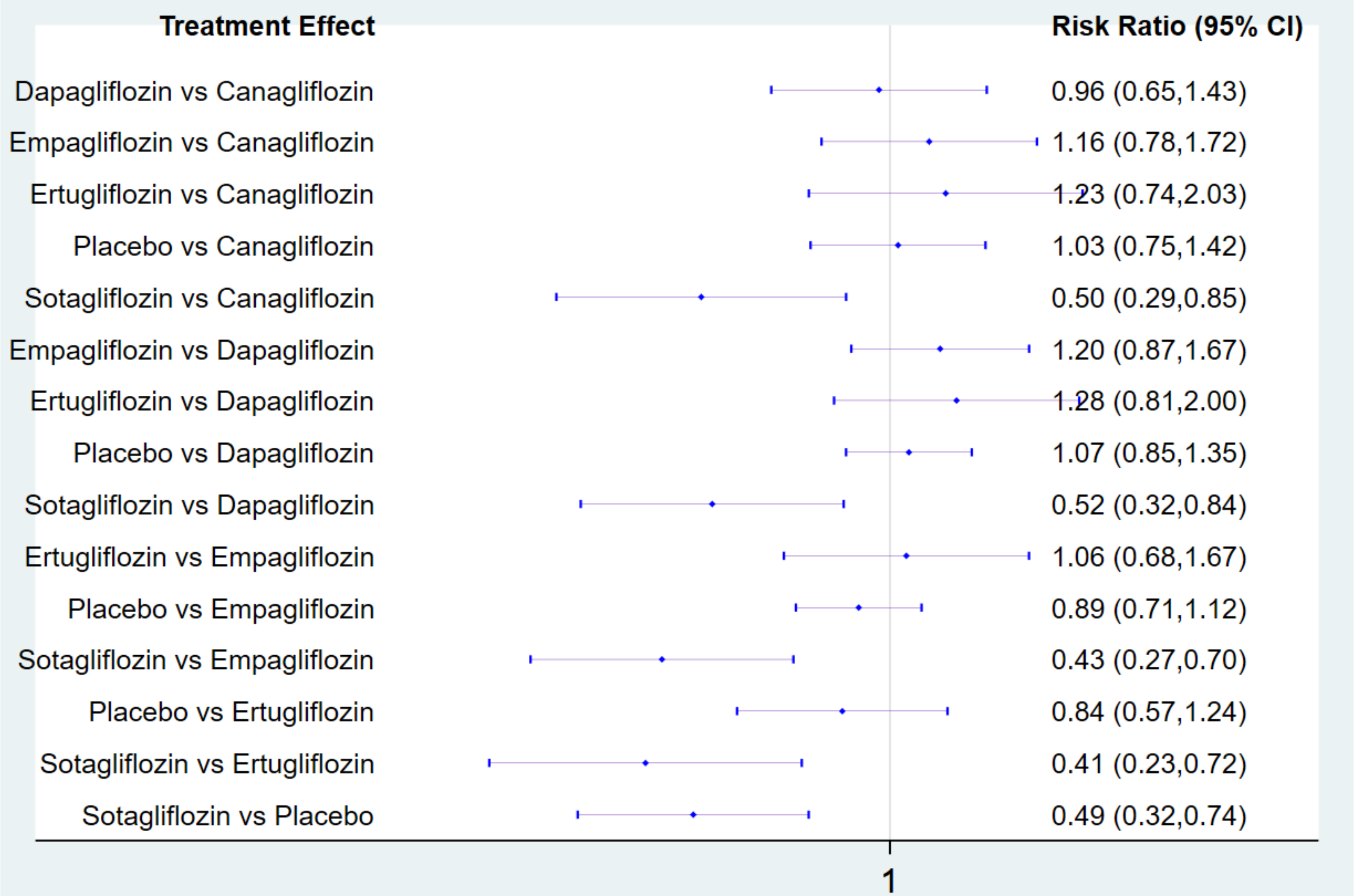

Figure S22 Forest plot of *Myocardial infarction*

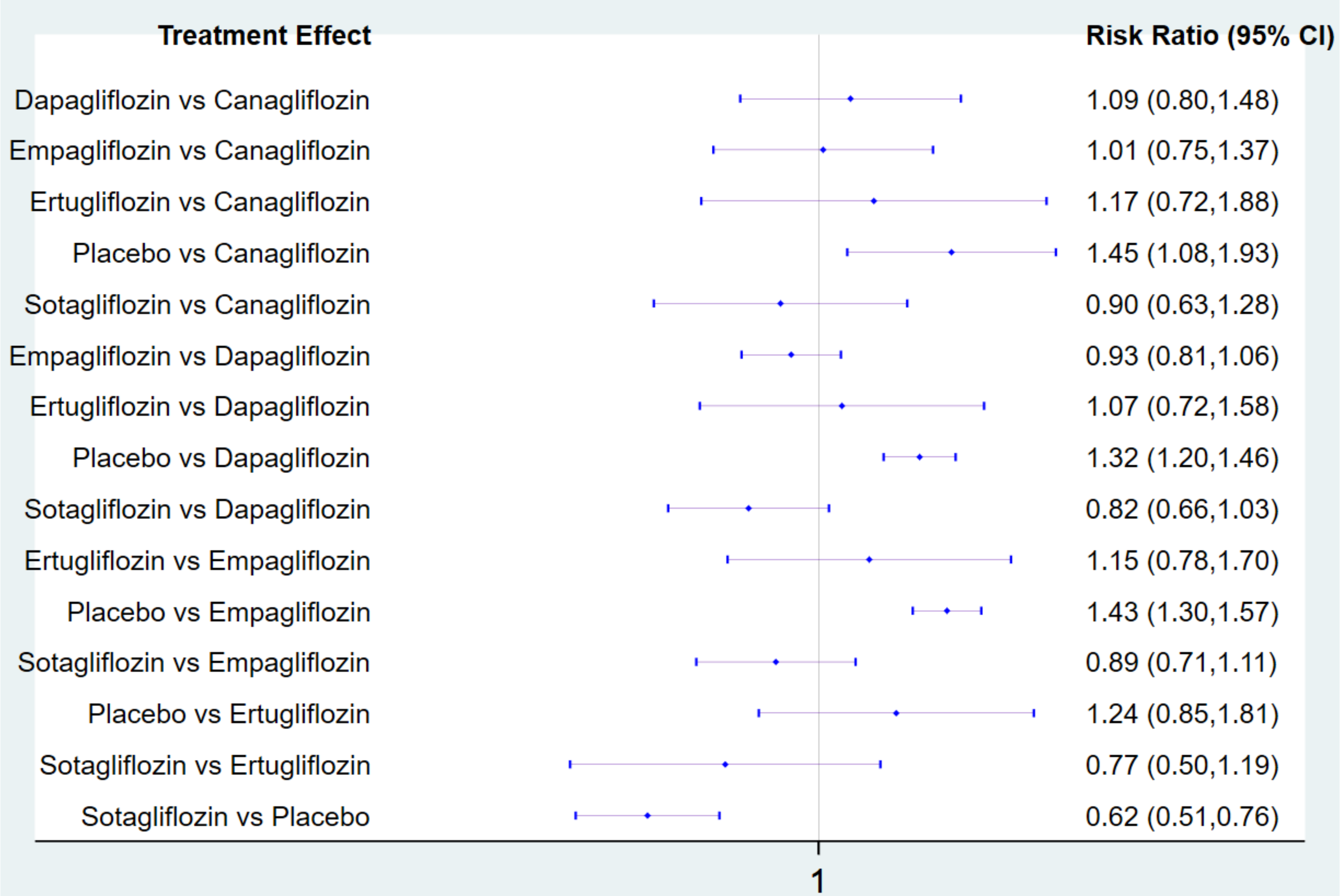

Figure S23 Forest plot of *Cardiac failure*

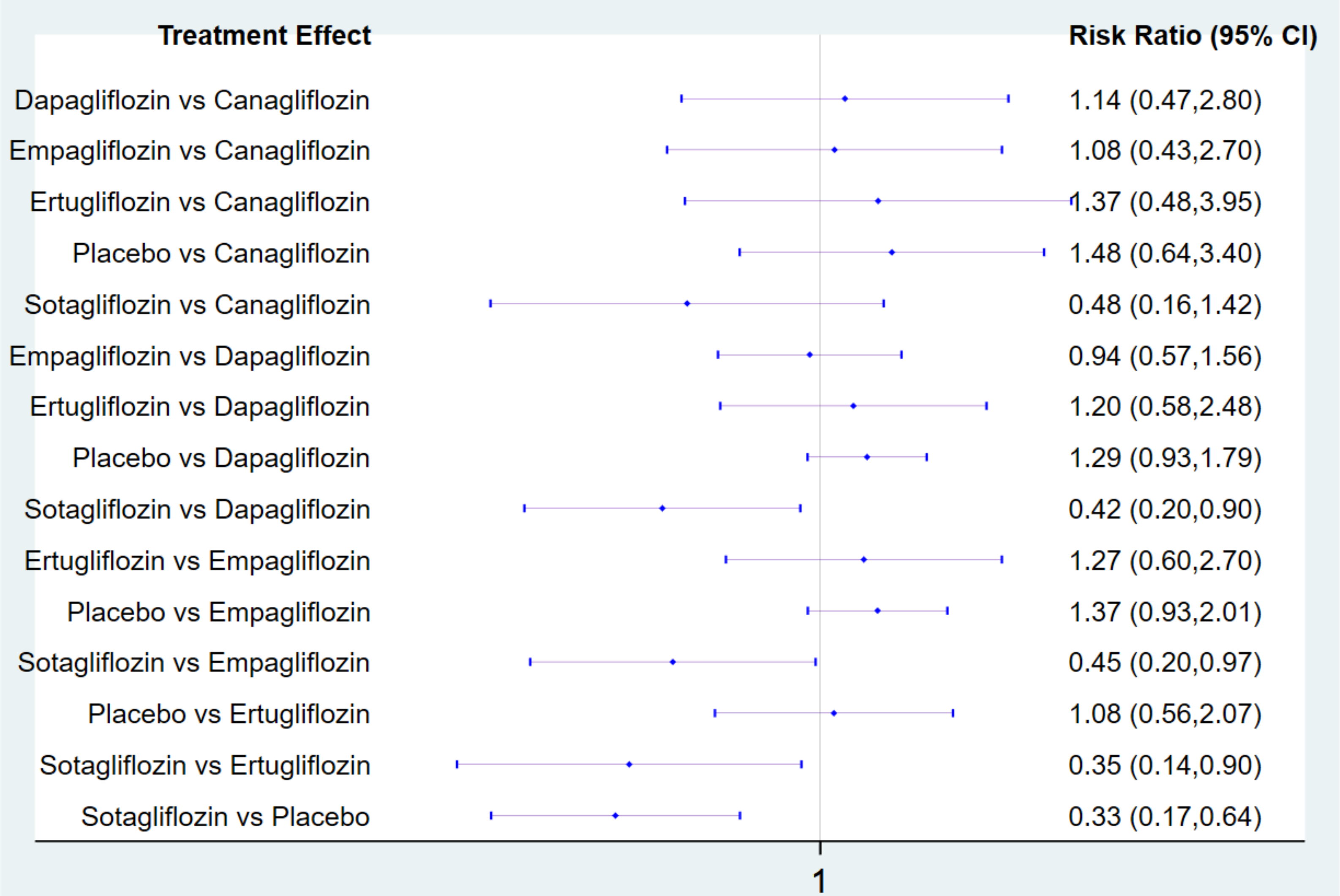

Figure S24 Forest plot of *Cardiac failure chronic*

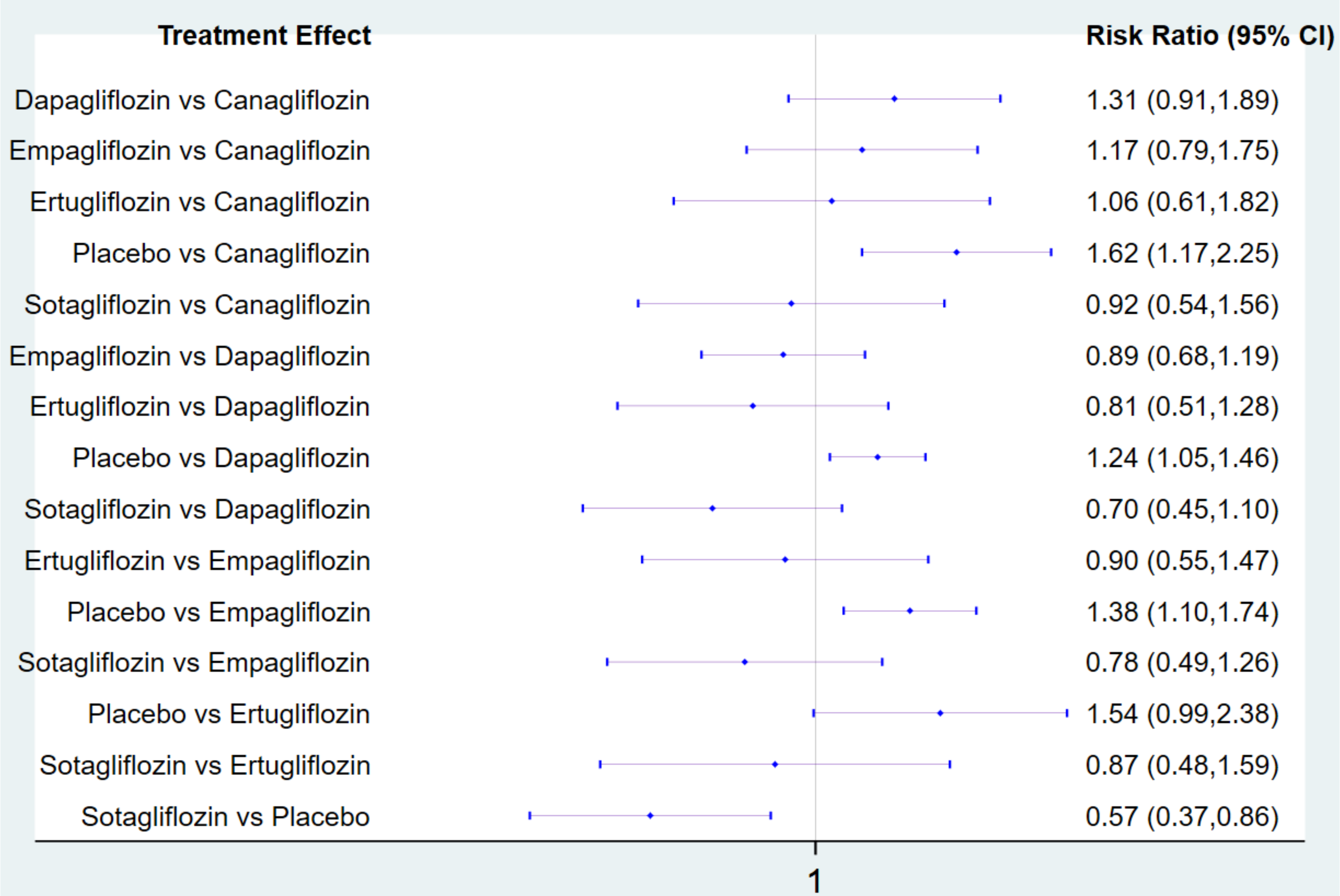

Figure S25 Forest plot of *Cardiac failure congestive*

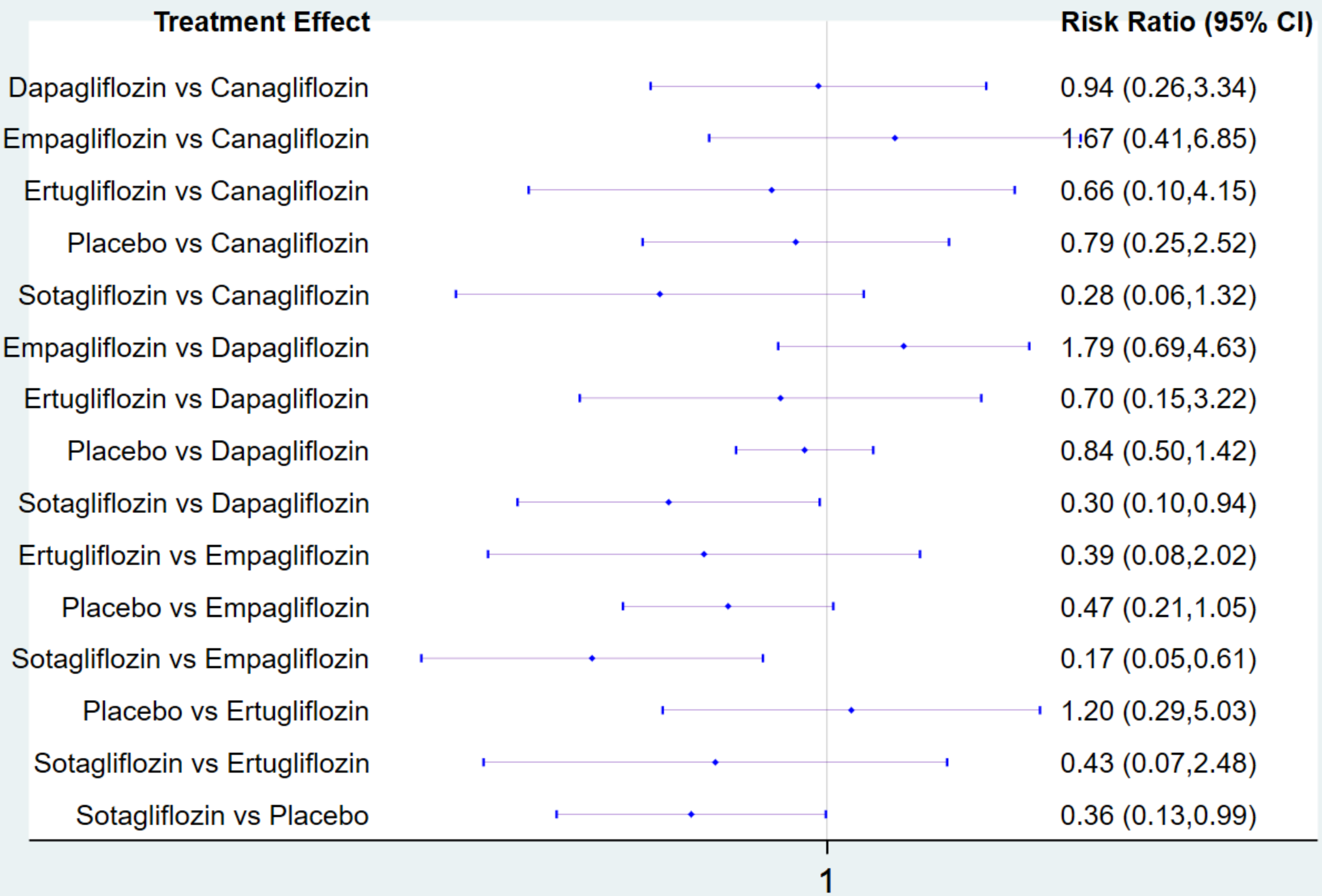

Figure S26 Forest plot of *Atrioventricular block complete*

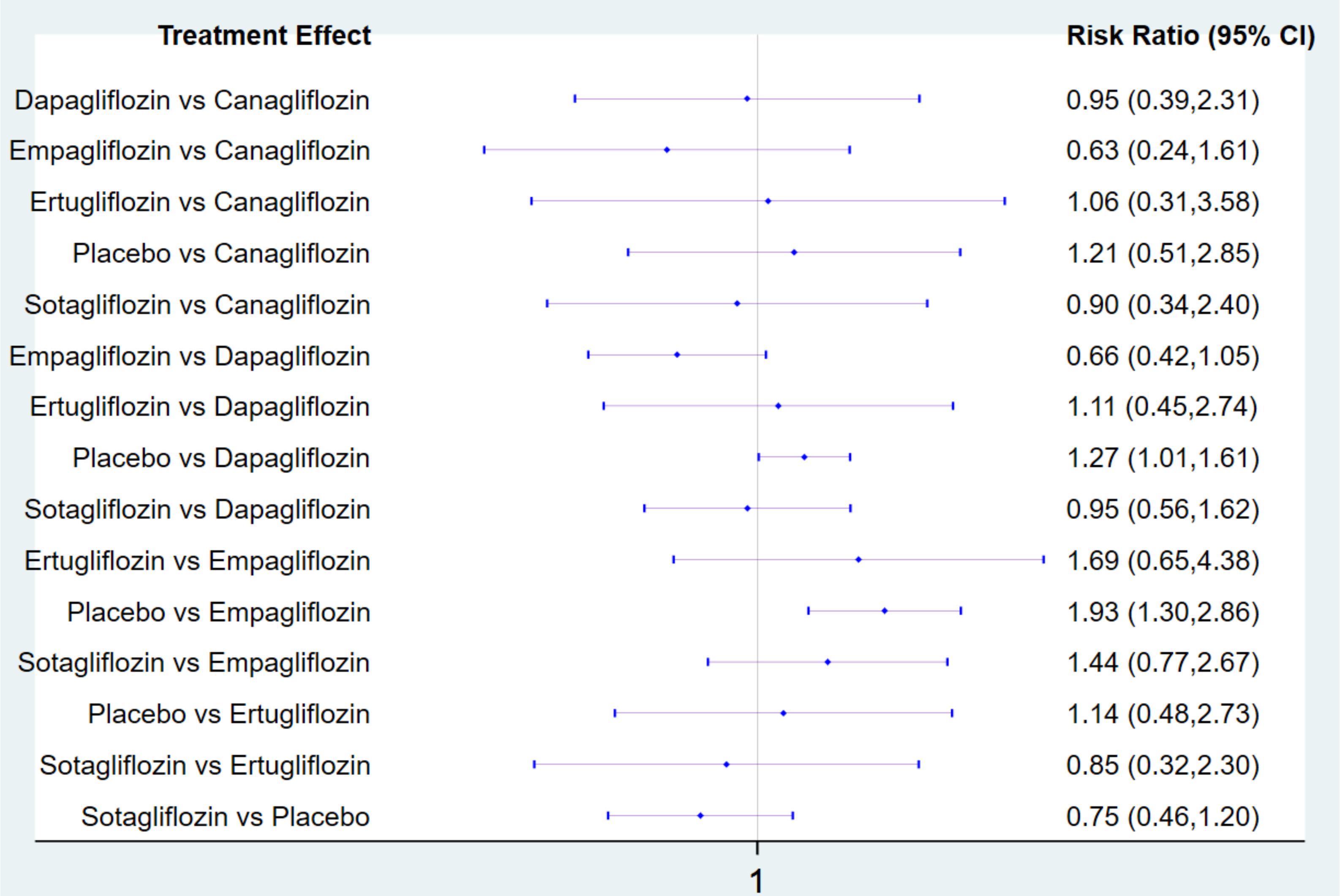

Figure S27 Forest plot of *Cardiac failure acute*

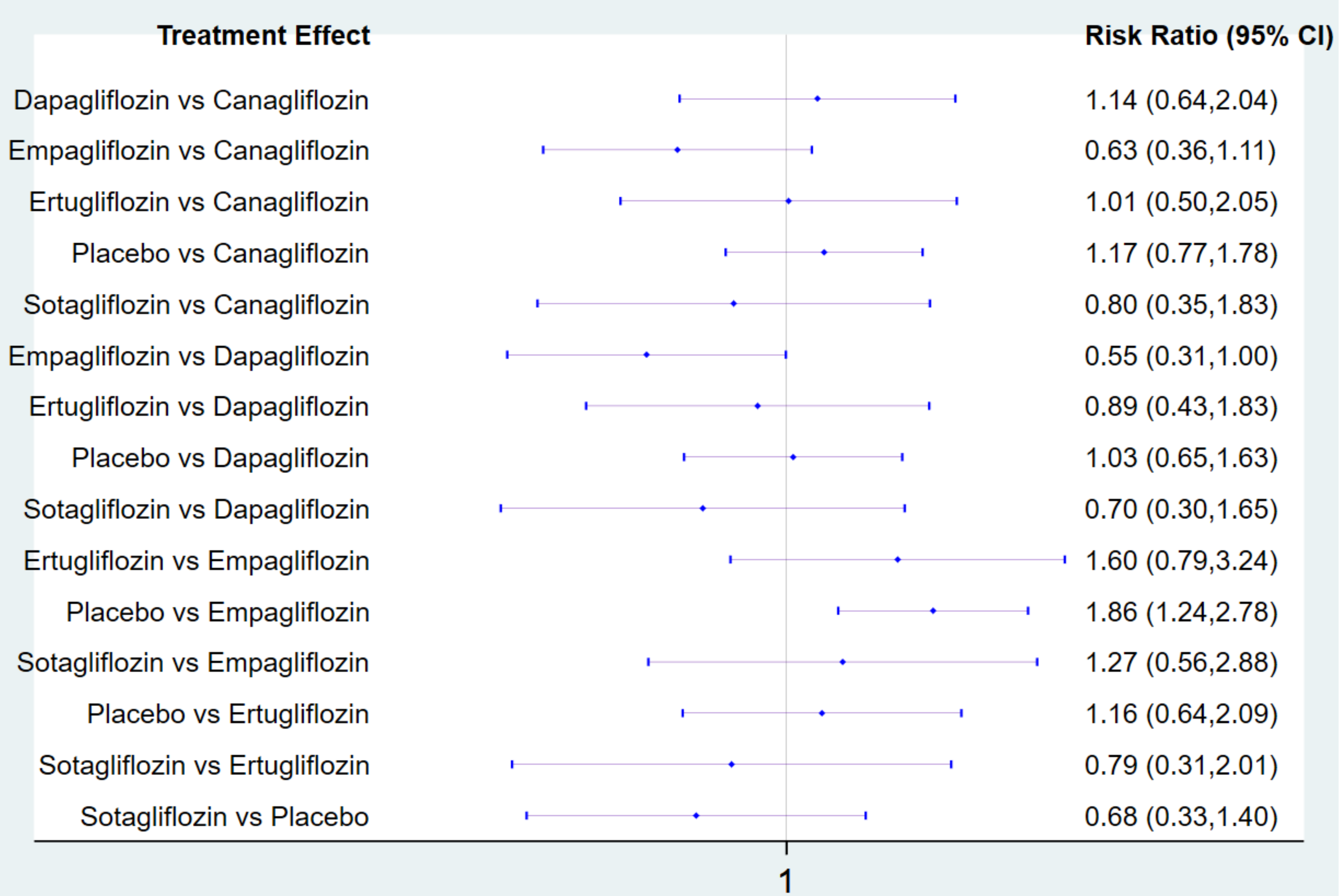

Figure S28 Forest plot of *Coronary artery disease*

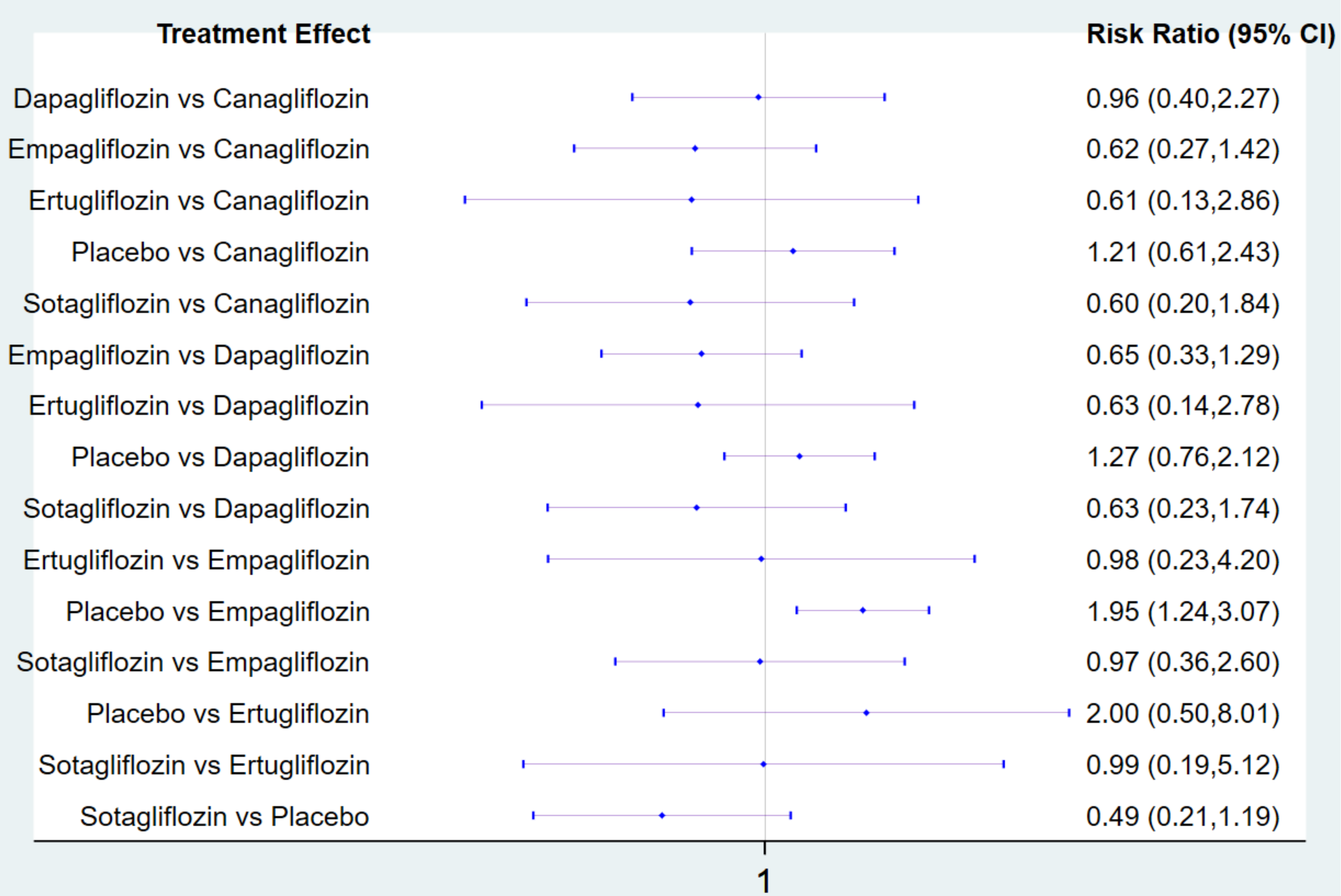

Figure S29 Forest plot of *Hypertensive crisis*

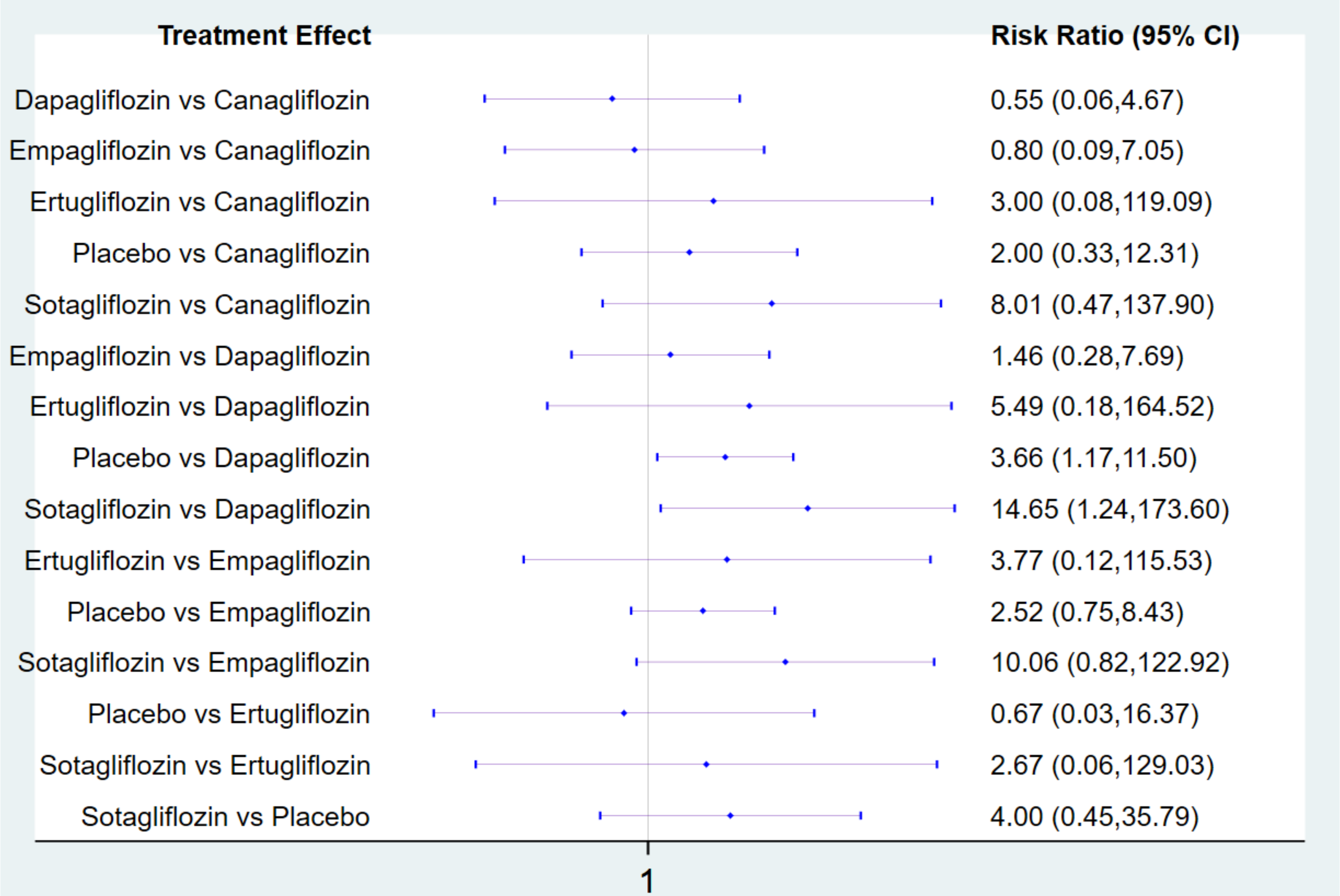

Figure S30 Forest plot of *Hypertensive emergency*

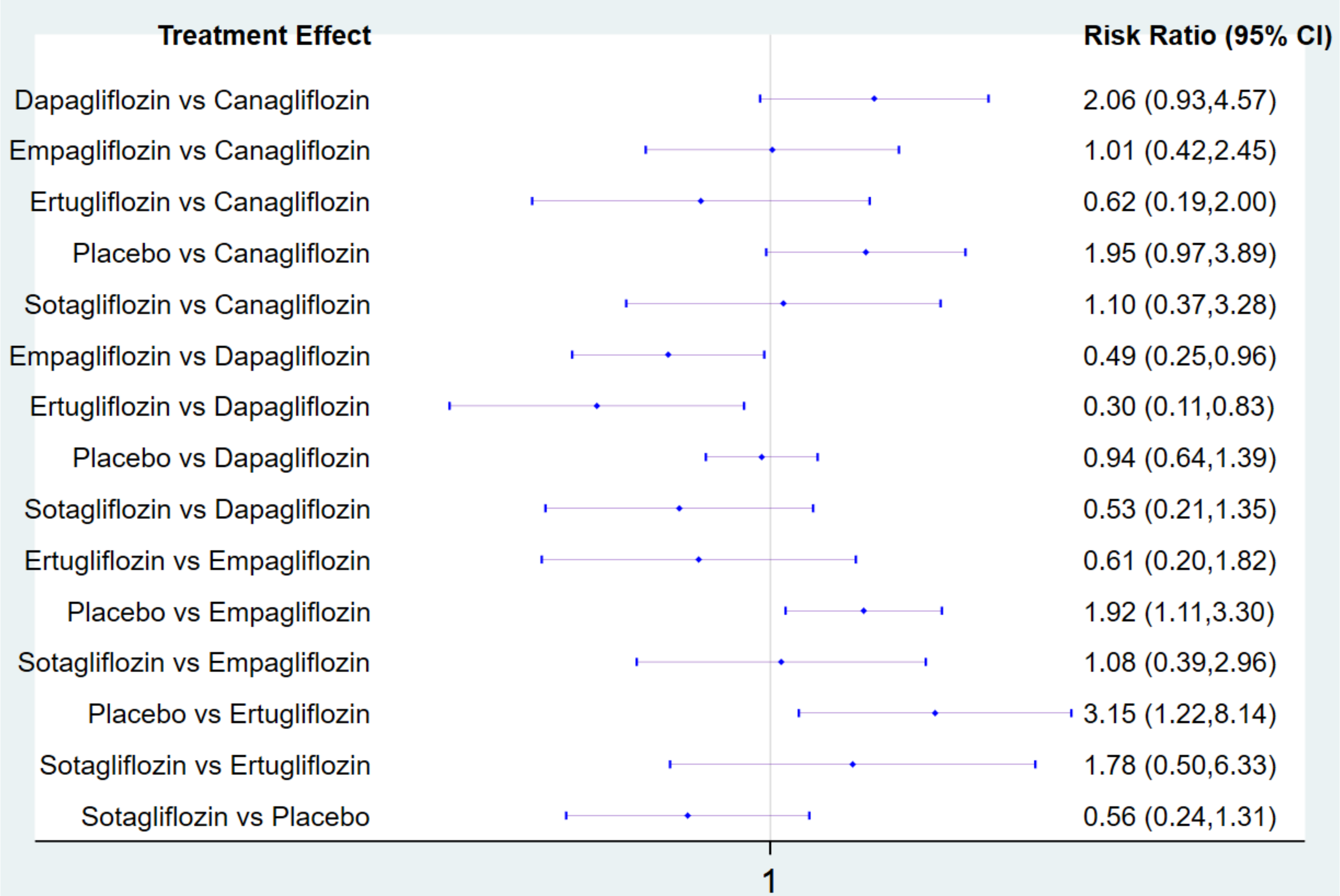

Figure S31 Forest plot of *Hypertension*

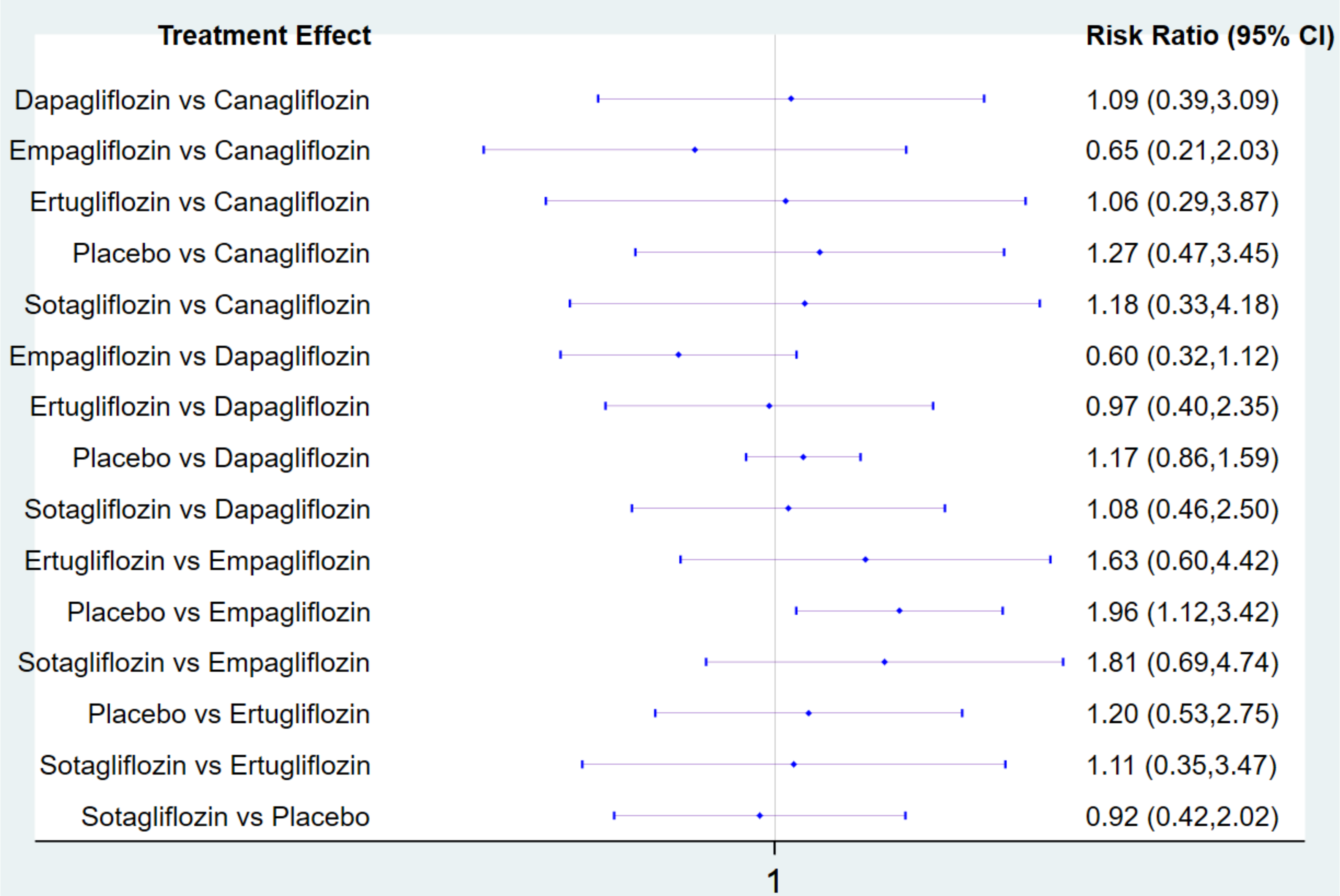

Figure S32 Forest plot of *Acute respiratory failure*

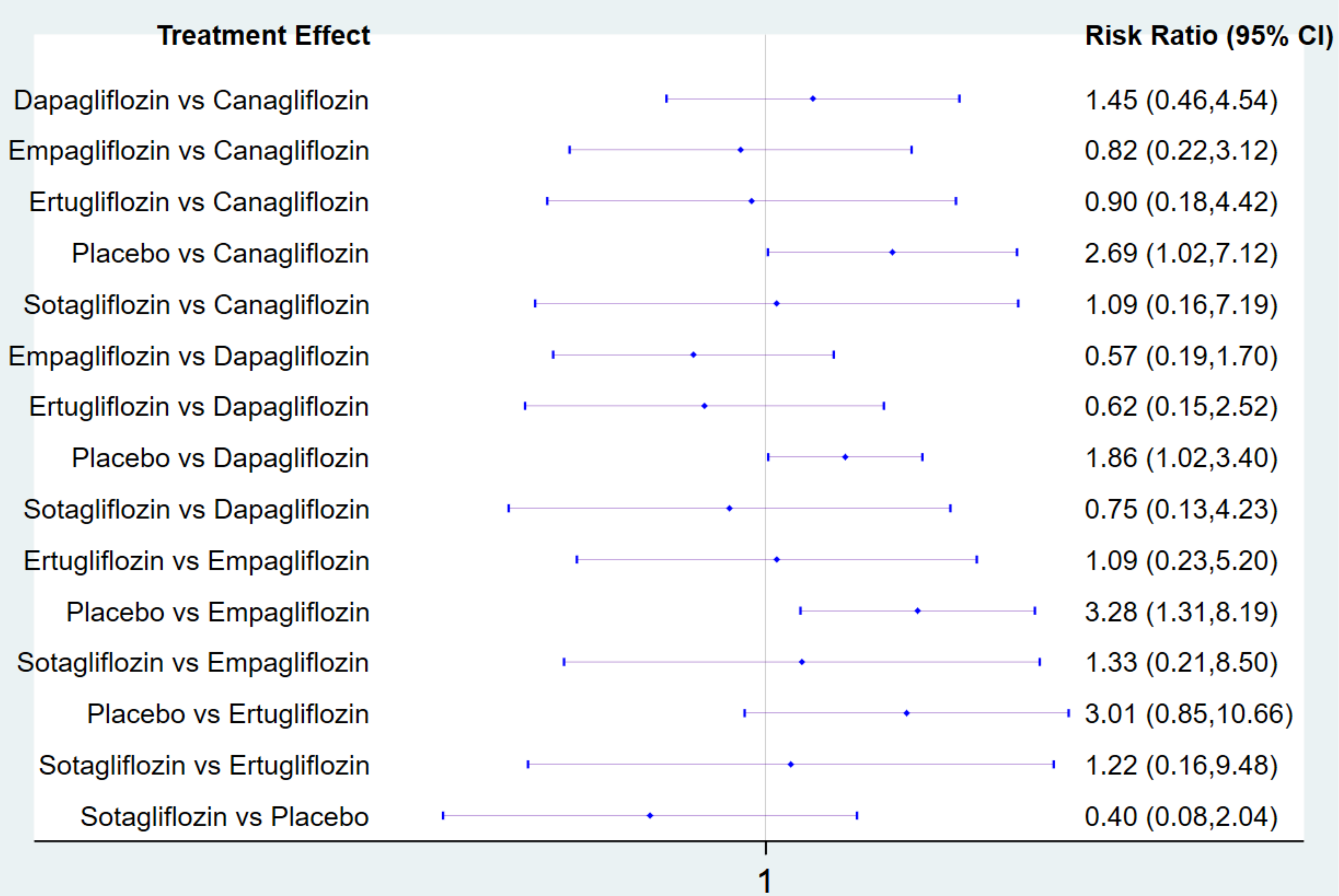

Figure S33 Forest plot of *Pulmonary oedema*

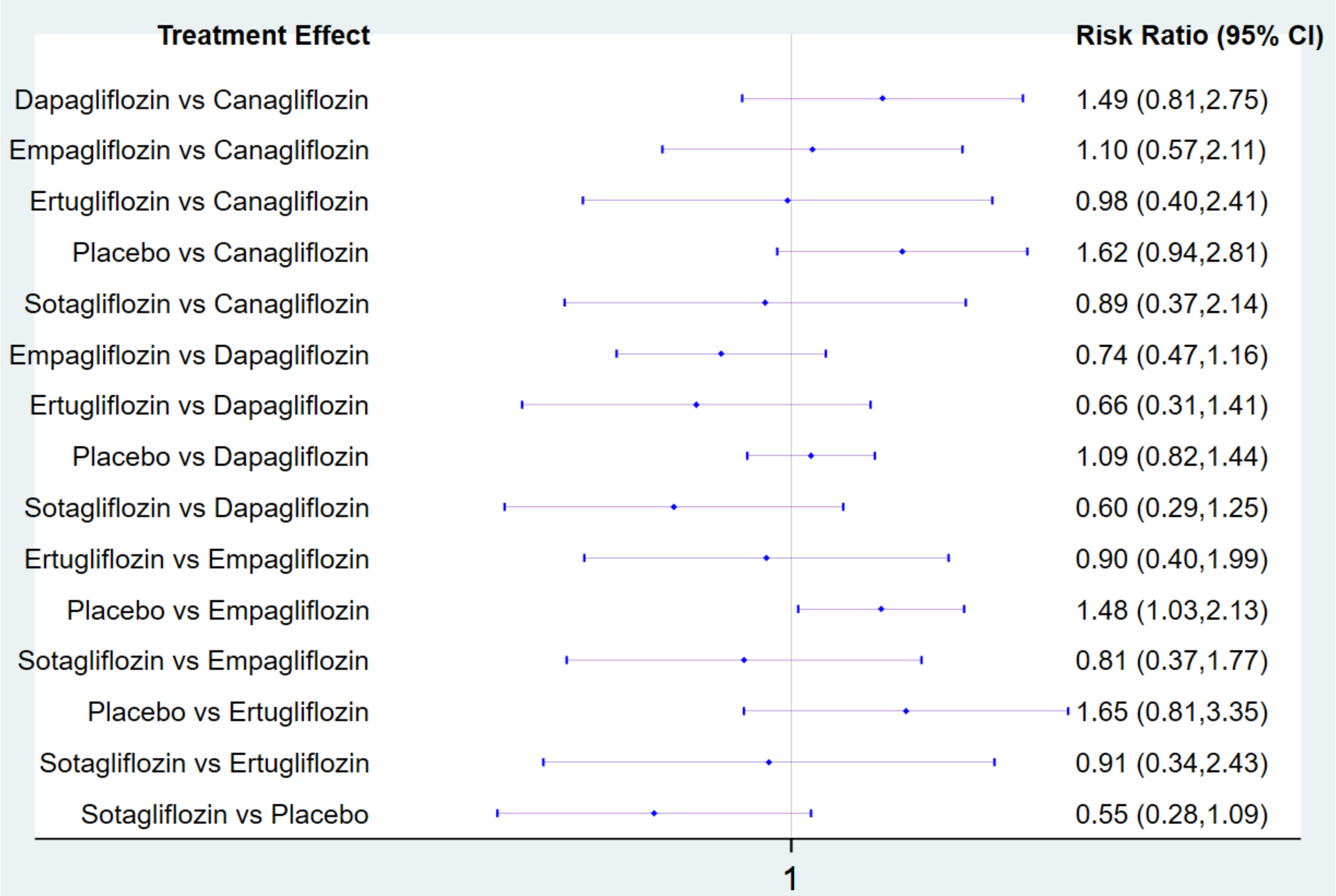

Figure S34 Forest plot of *Chronic obstructive pulmonary disease*

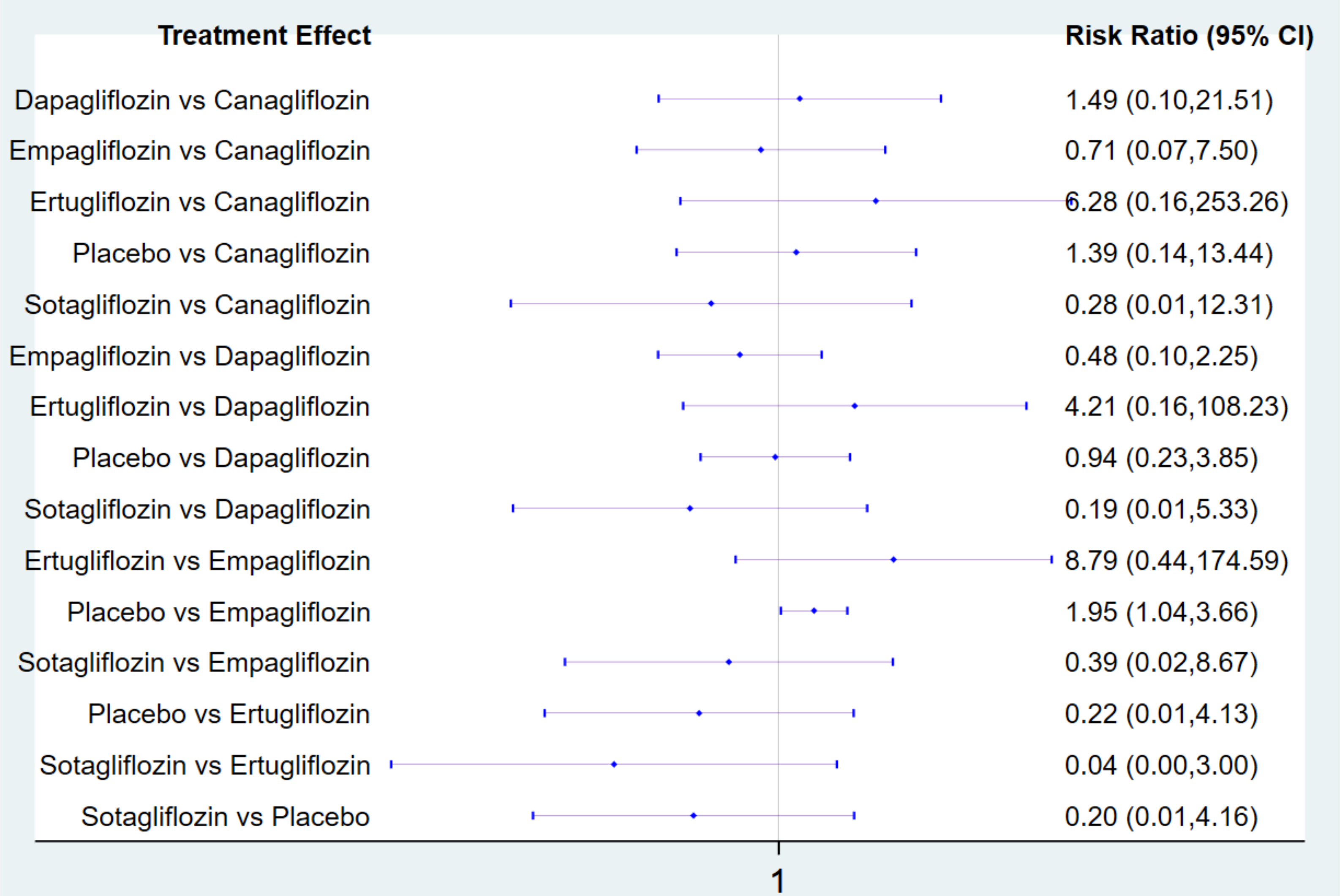

Figure S35 Forest plot of *Pulmonary hypertension*

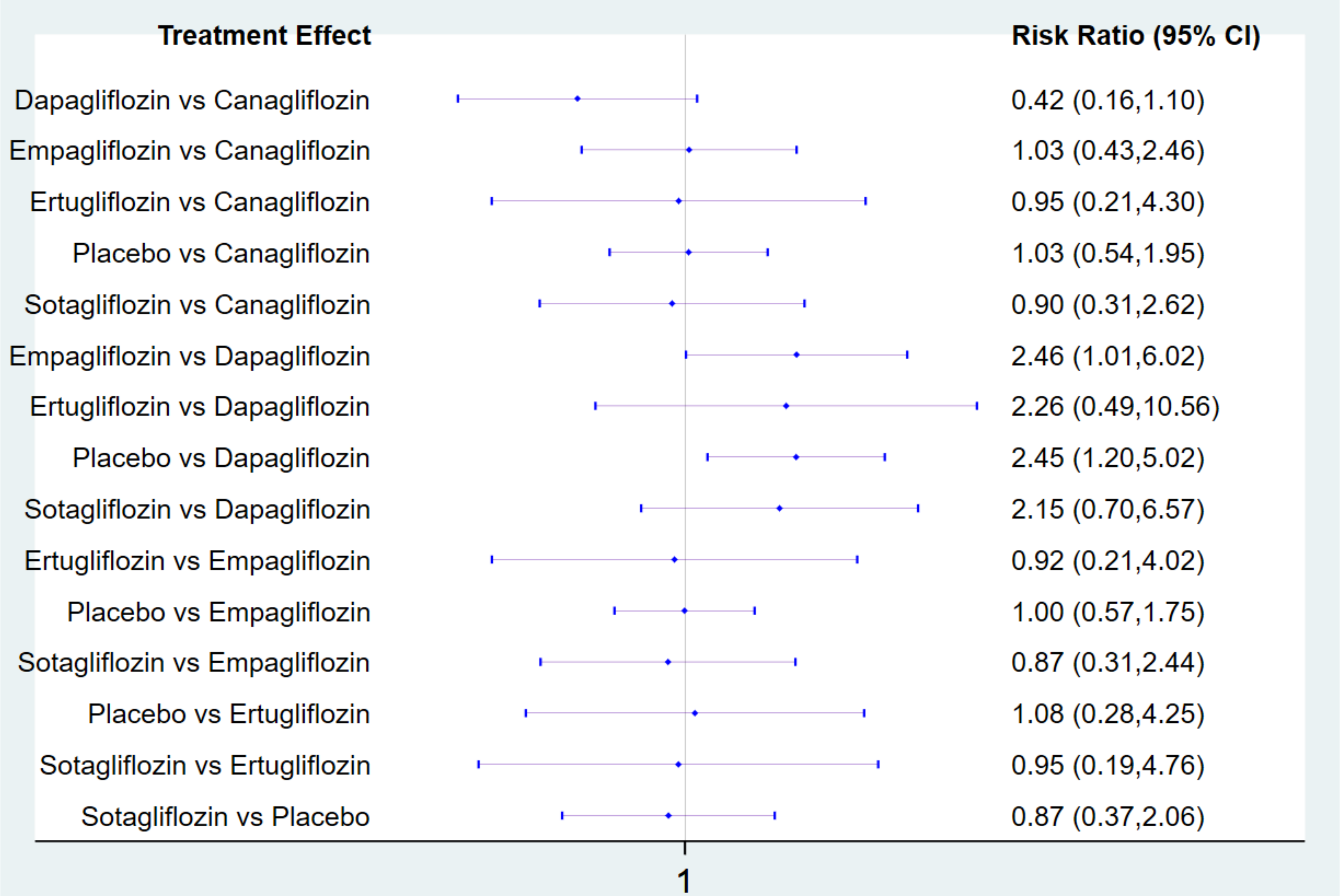

Figure S36 Forest plot of *Dyspnoea*

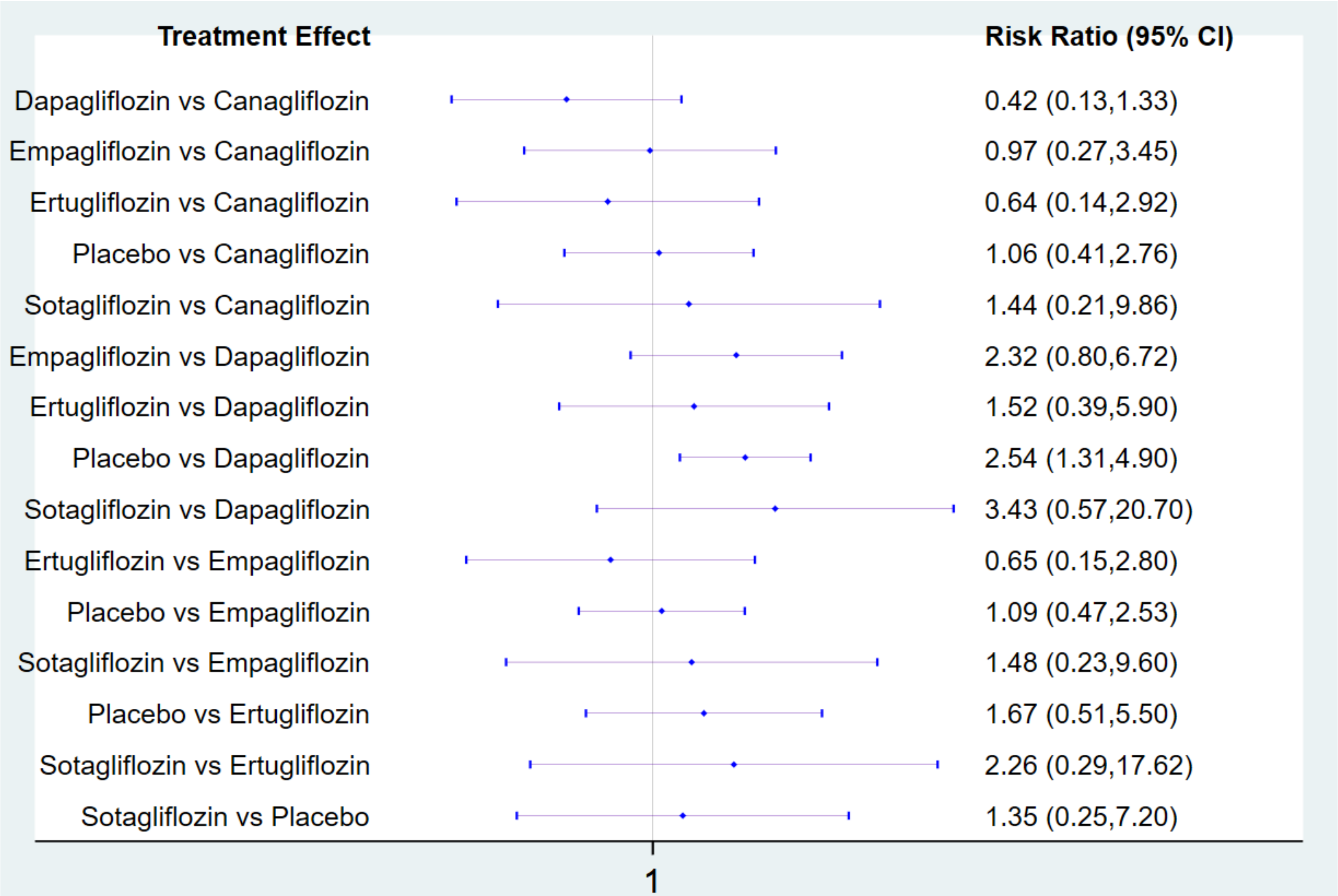

Figure S37 Forest plot of *Asthma*

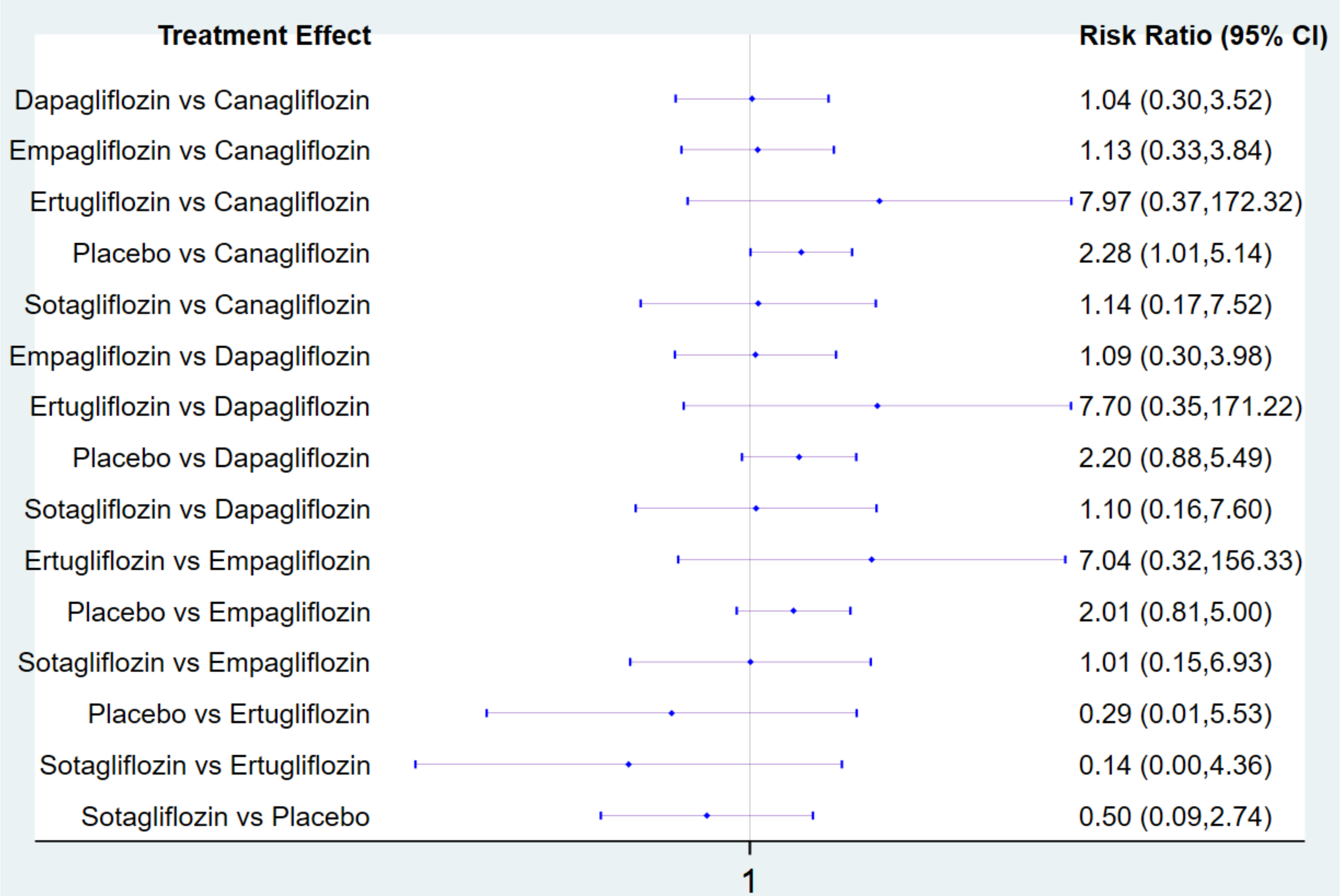

Figure S38 Forest plot of *Respiratory tract infection*

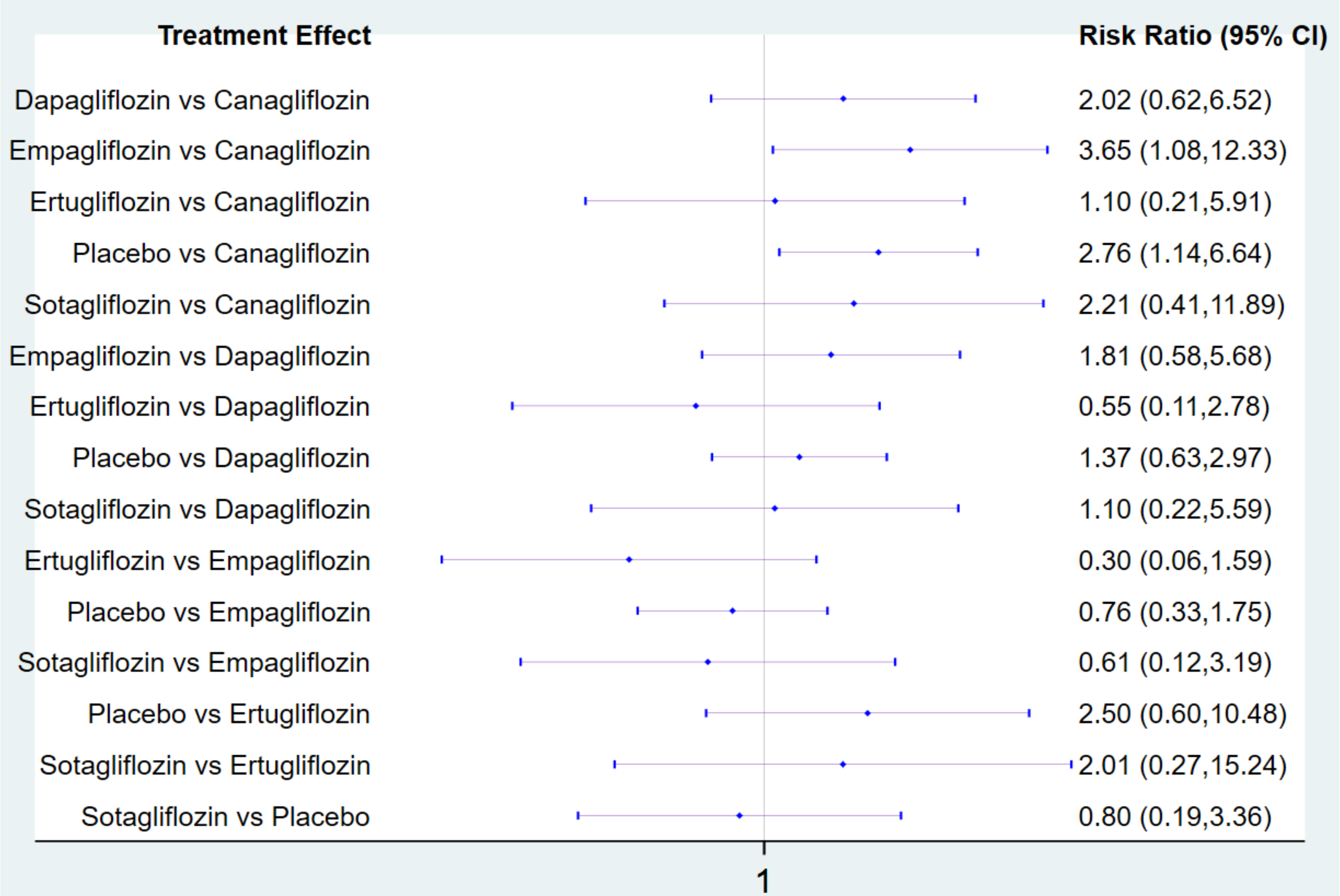

Figure S39 Forest plot of *Lower respiratory tract infection*

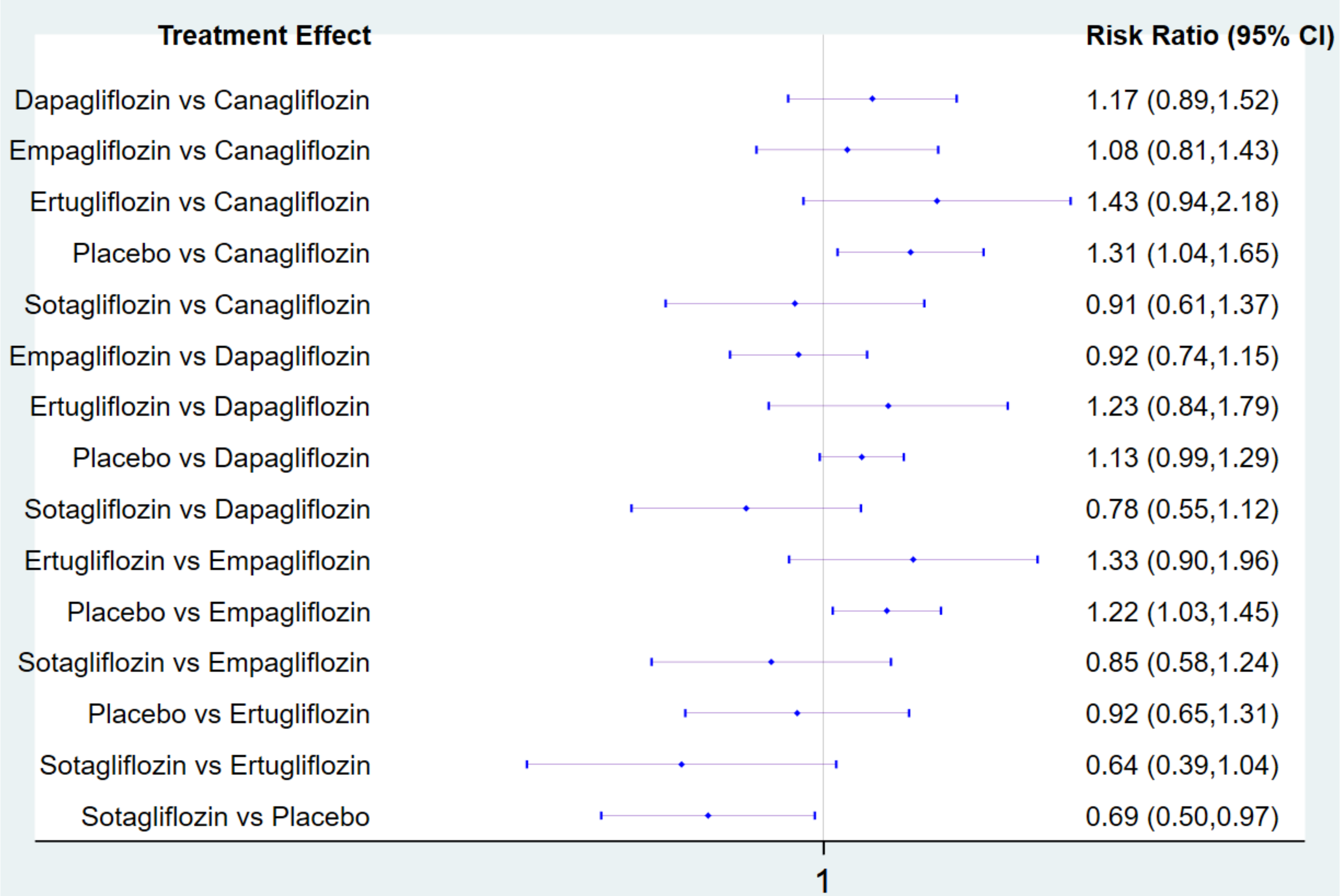

Figure S40 Forest plot of *Pneumonia*

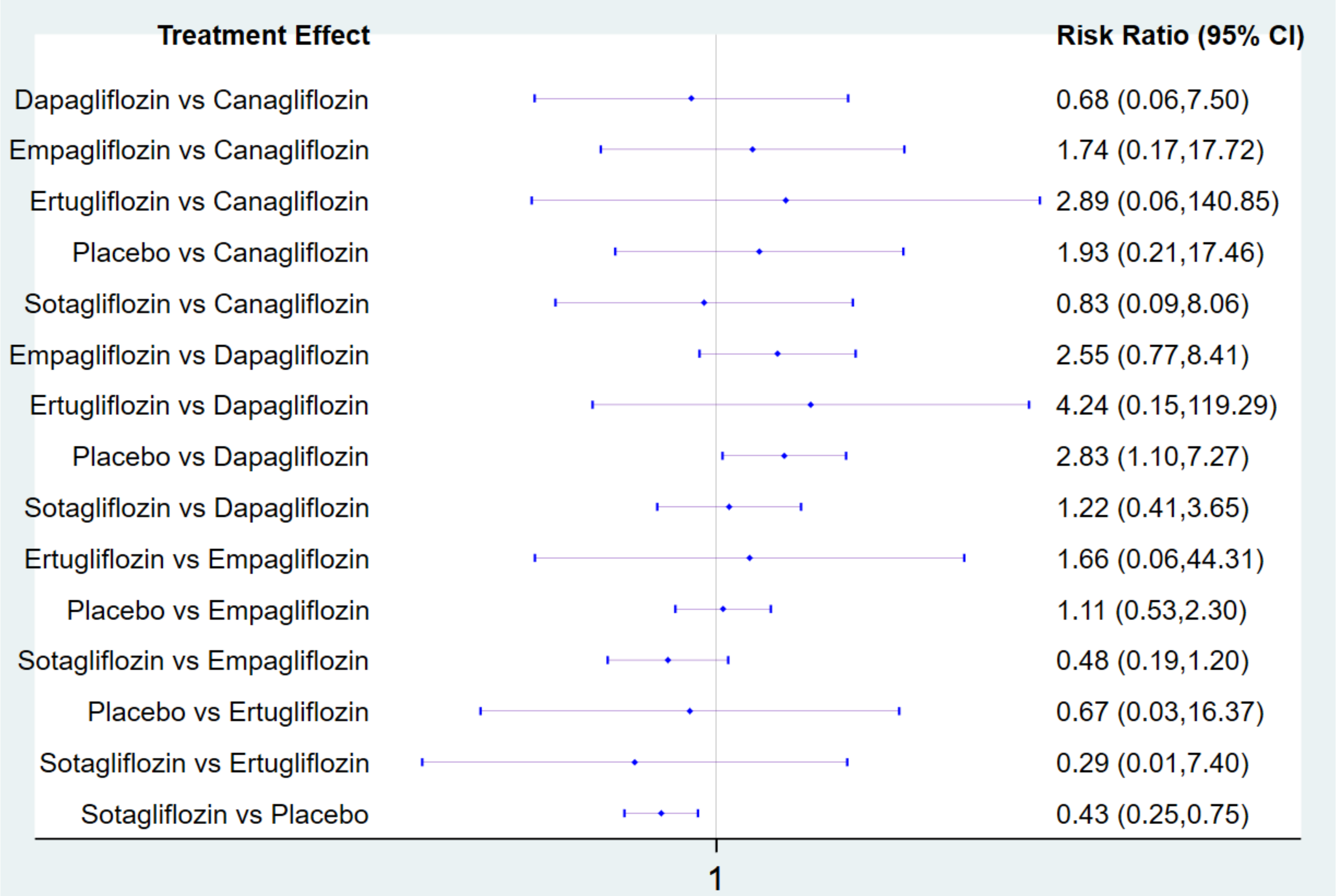

Figure S41 Forest plot of *Pneumonia bacterial*

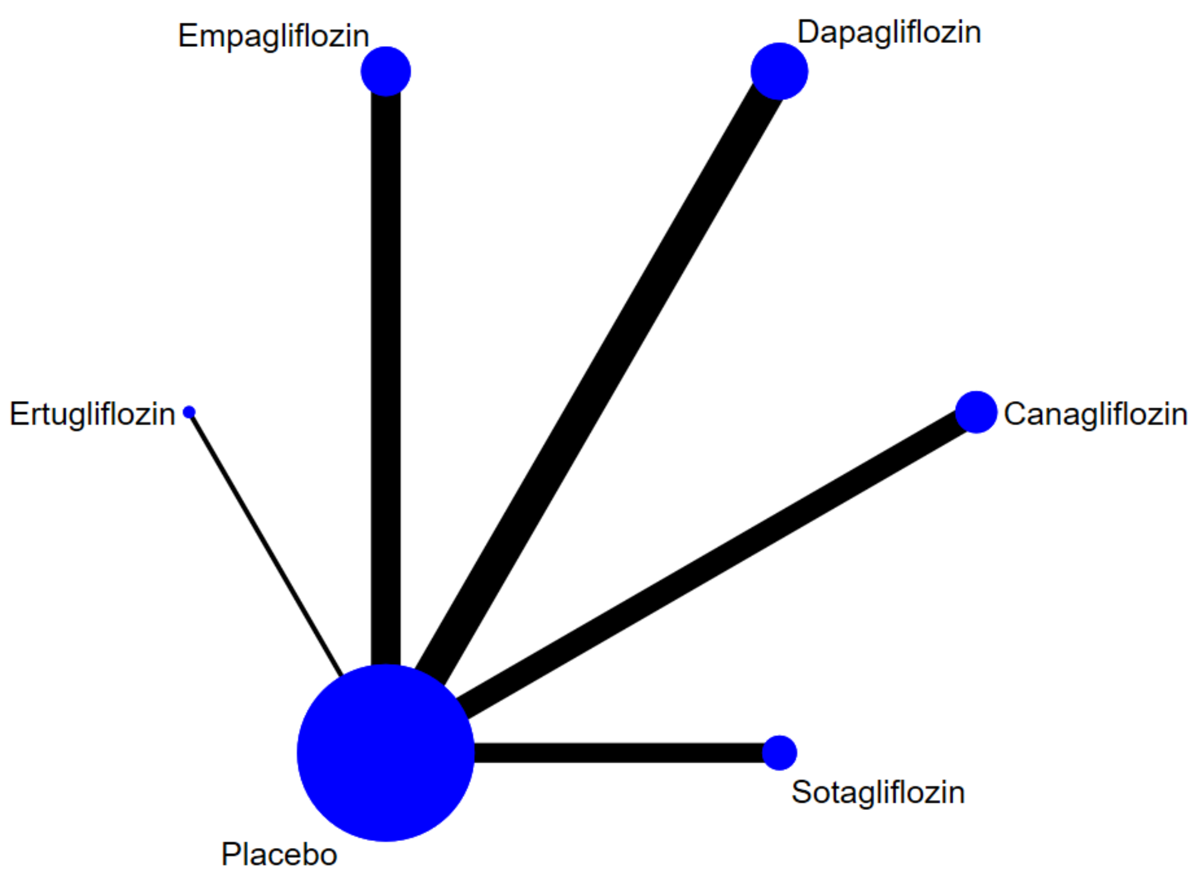

Figure S42 Network plot of *Myocardial infarction*

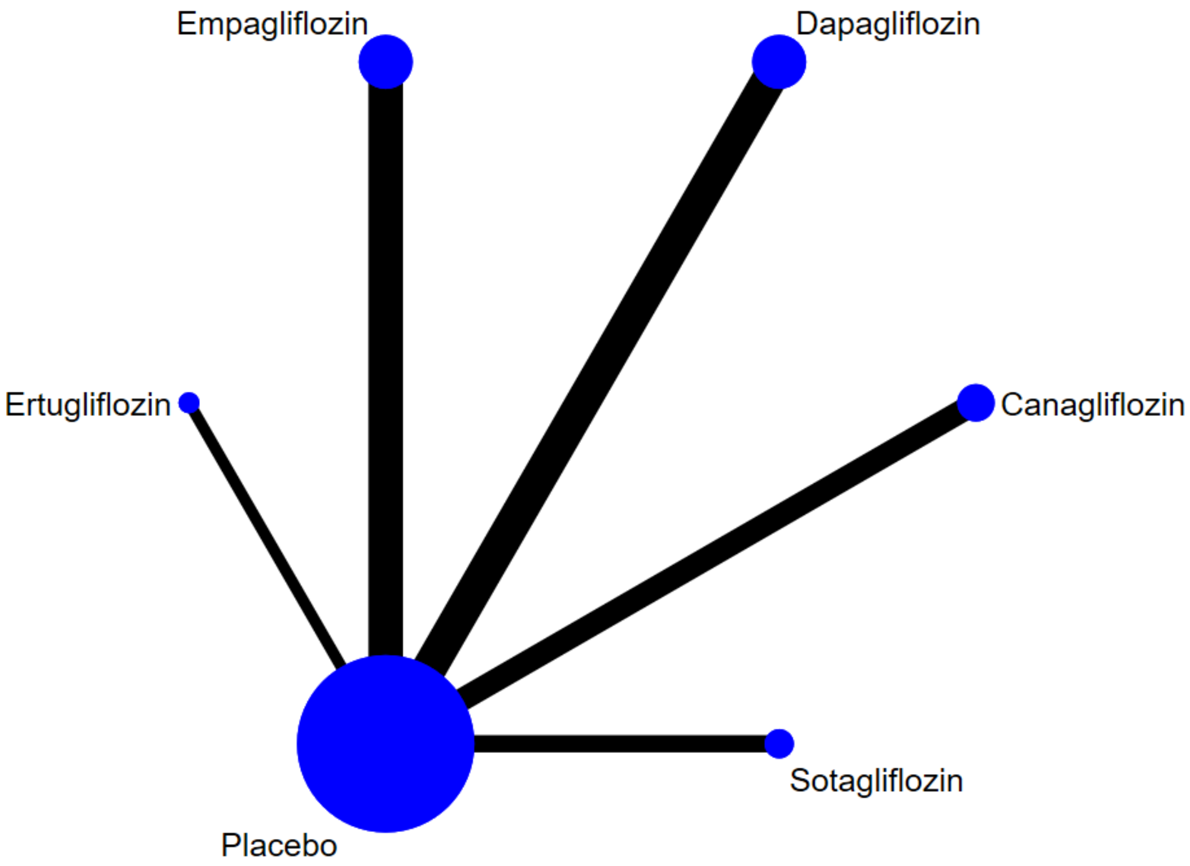

Figure S43 Network plot of *Cardiac failure*

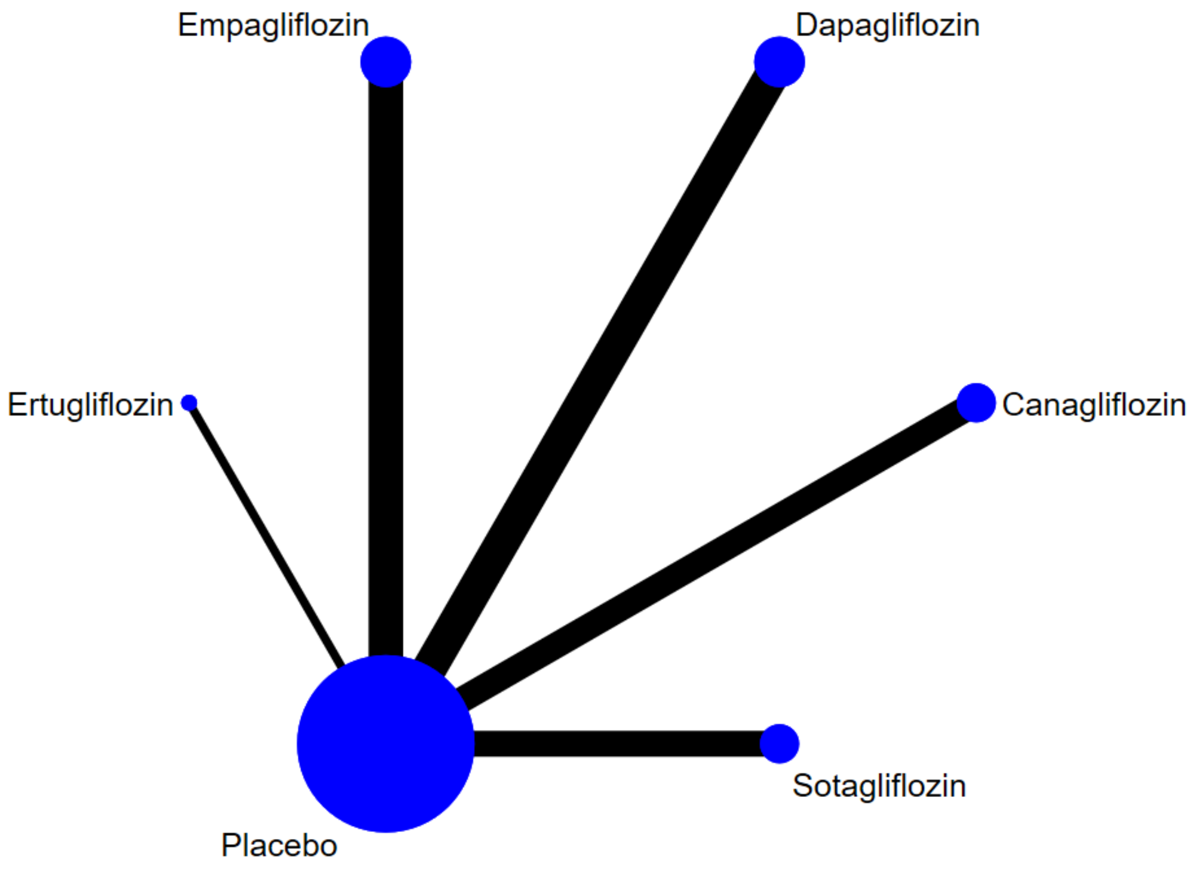

Figure S44 Network plot of *Cardiac failure chronic*

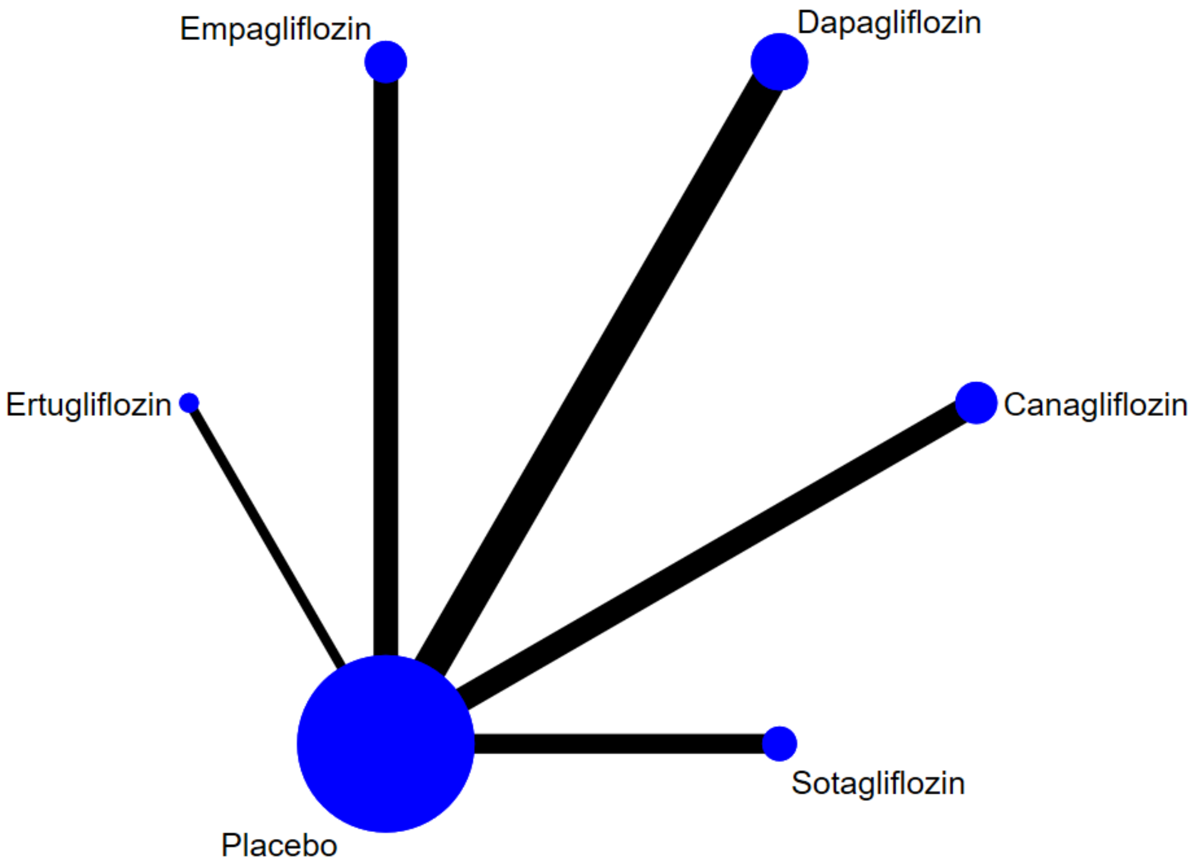

Figure S45 Network plot of *Cardiac failure congestive*

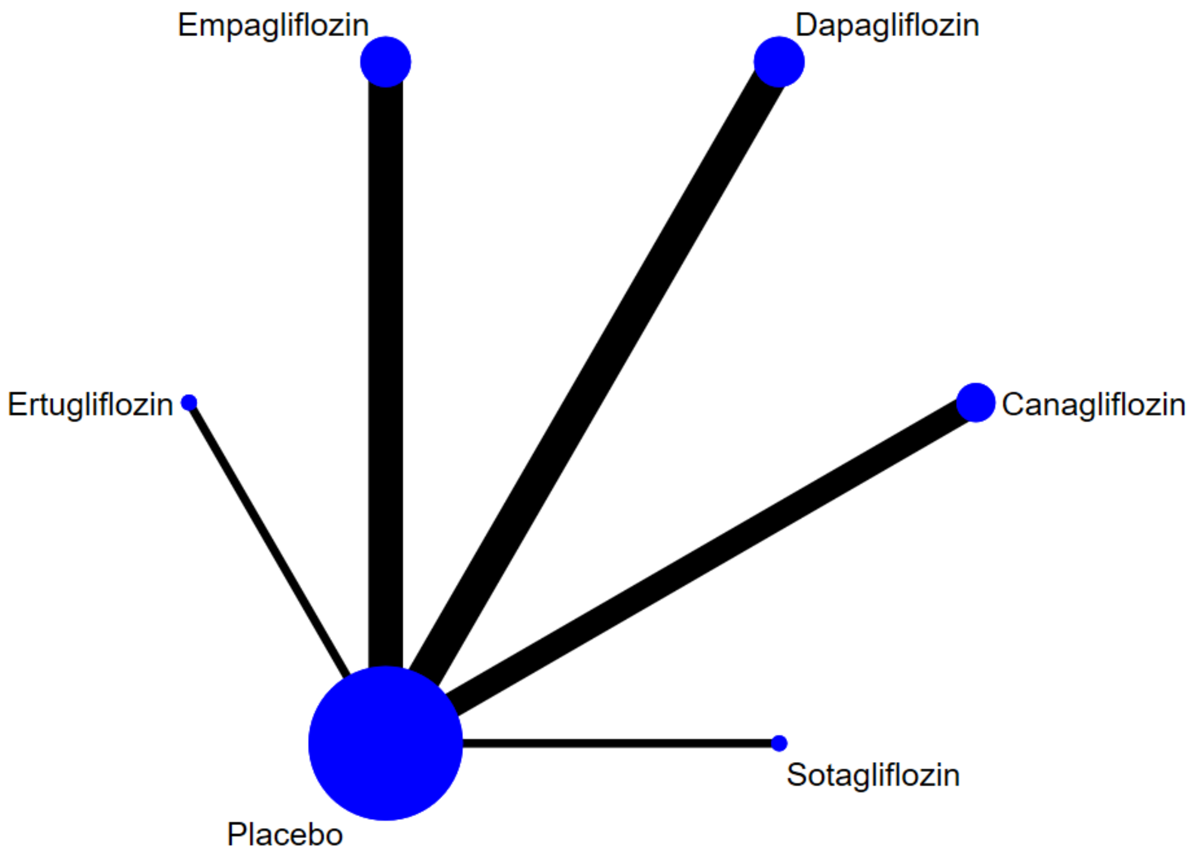

Figure S46 Network plot of *Atrioventricular block complete*

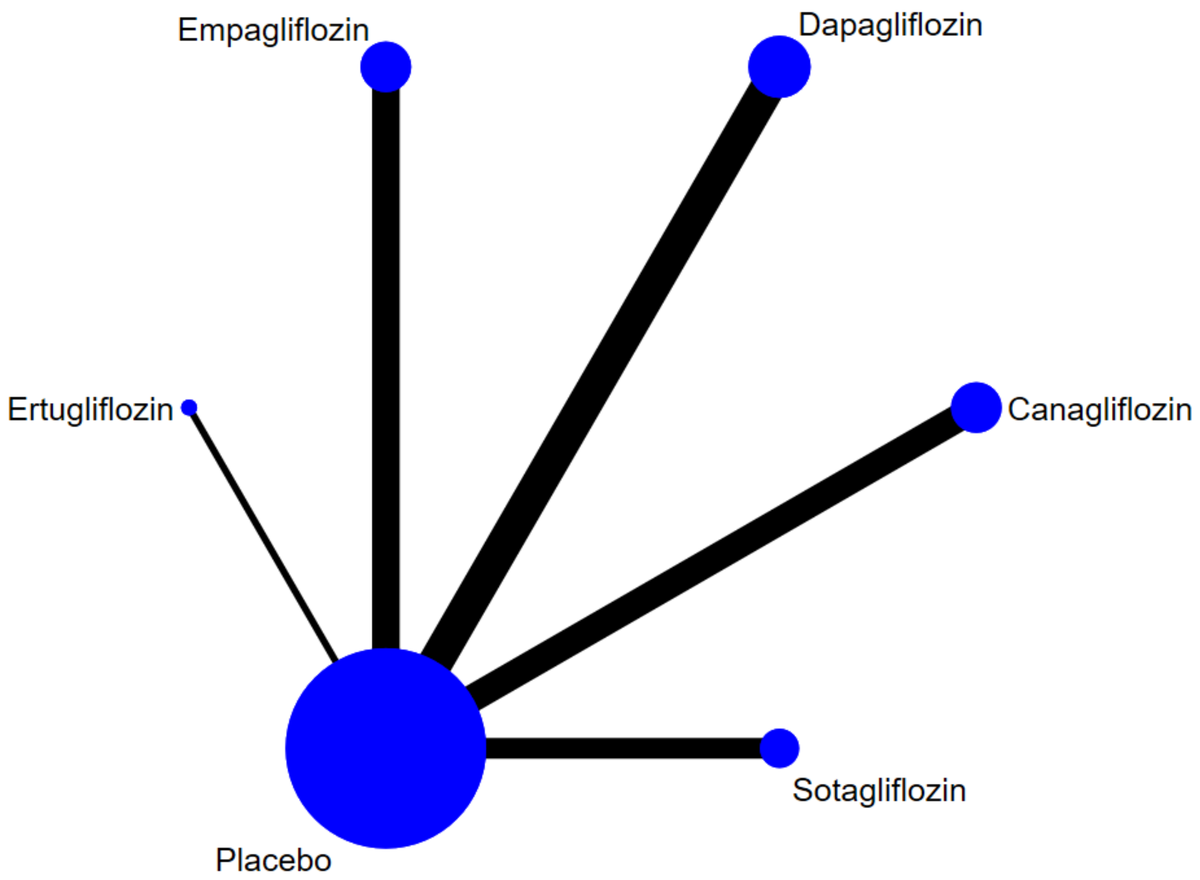

Figure S47 Network plot of *Cardiac failure acute*

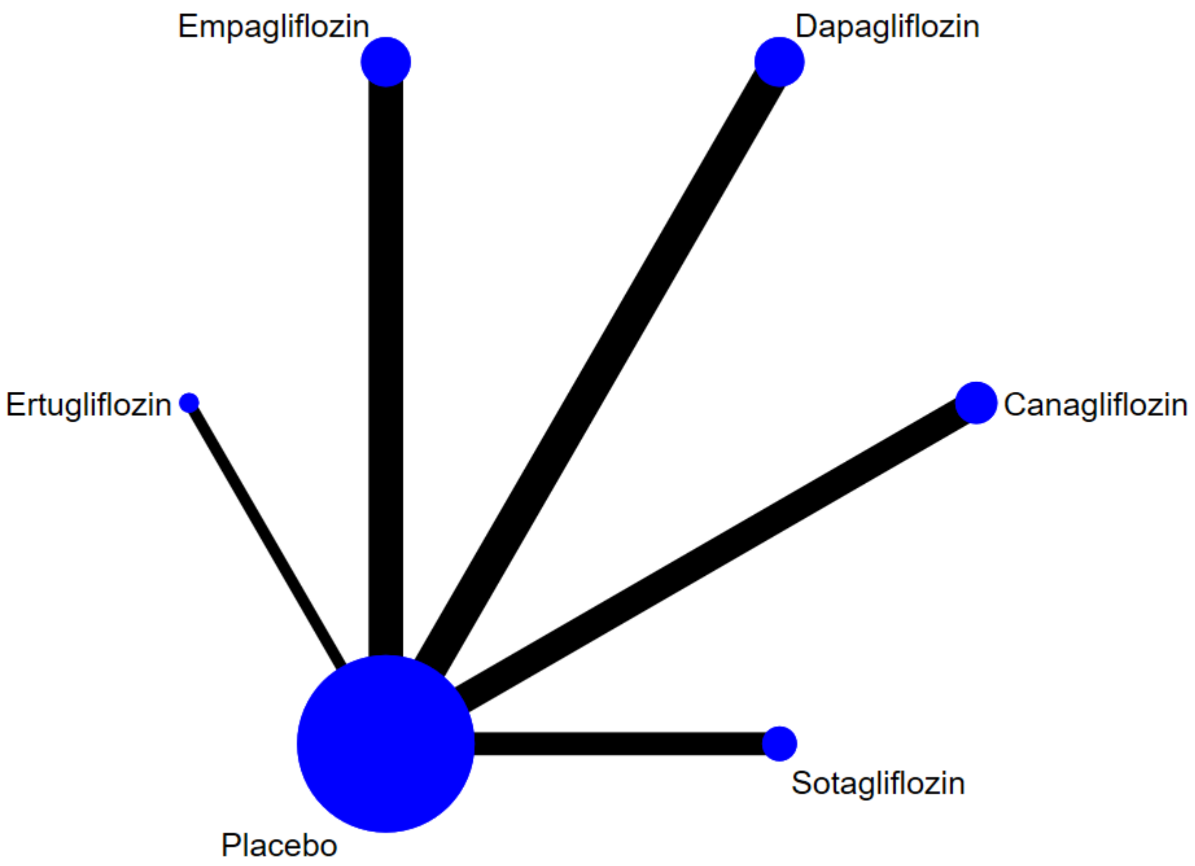

Figure S48 Network plot of *Coronary artery disease*

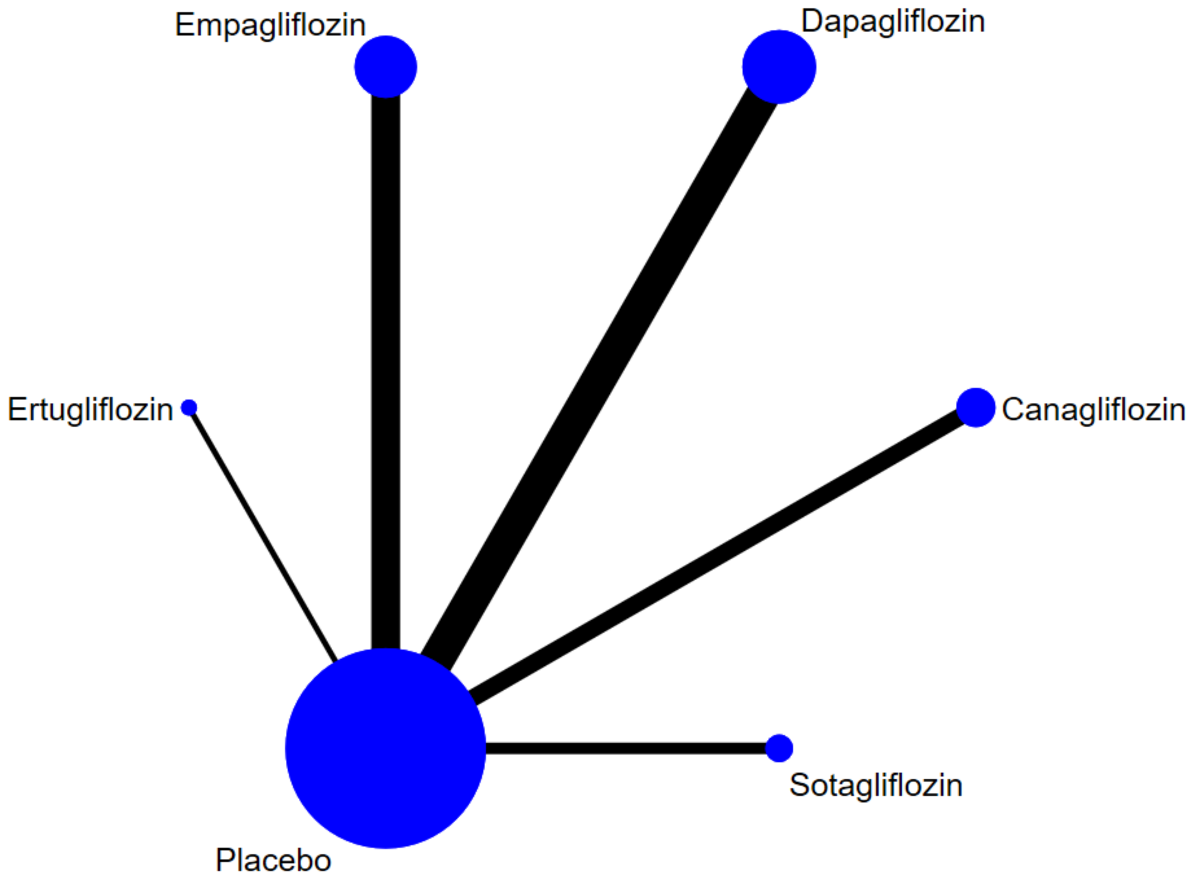

Figure S49 Network plot of *Hypertensive crisis*

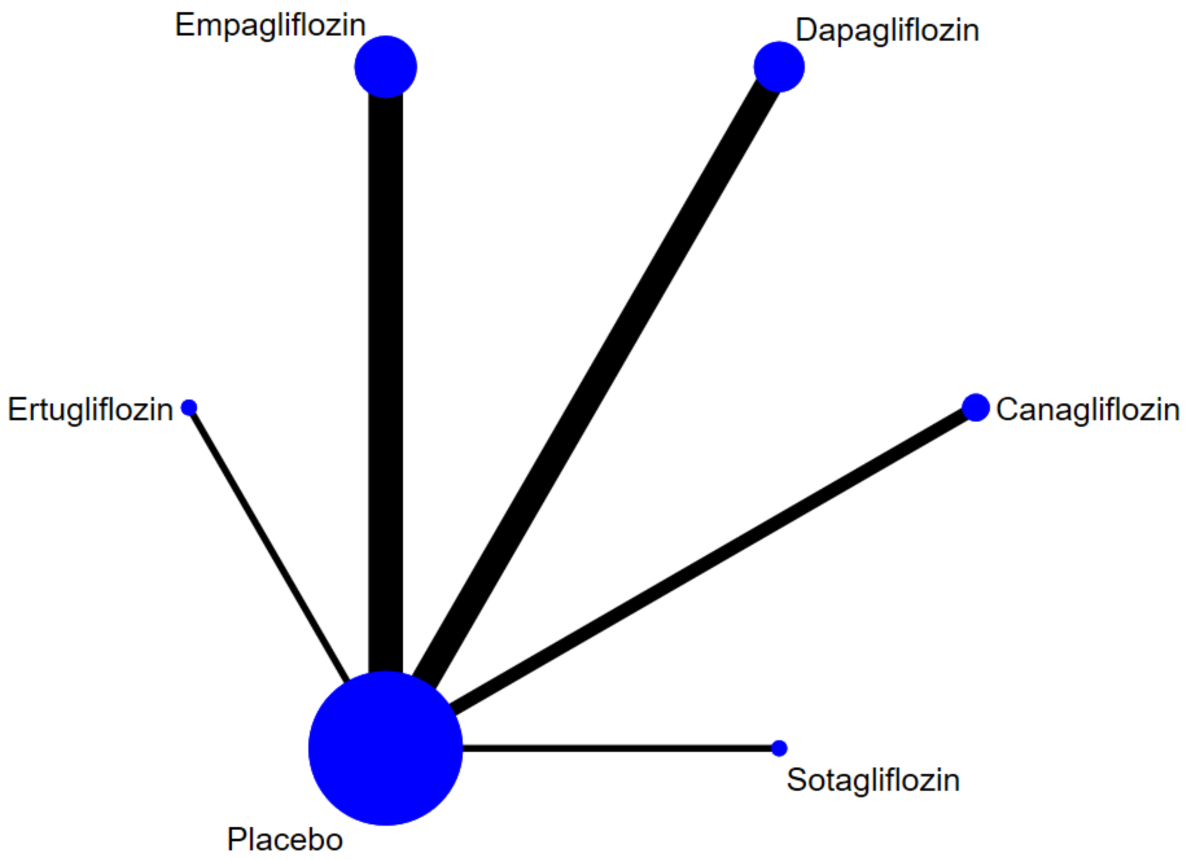

Figure S50 Network plot of *Hypertensive emergency*

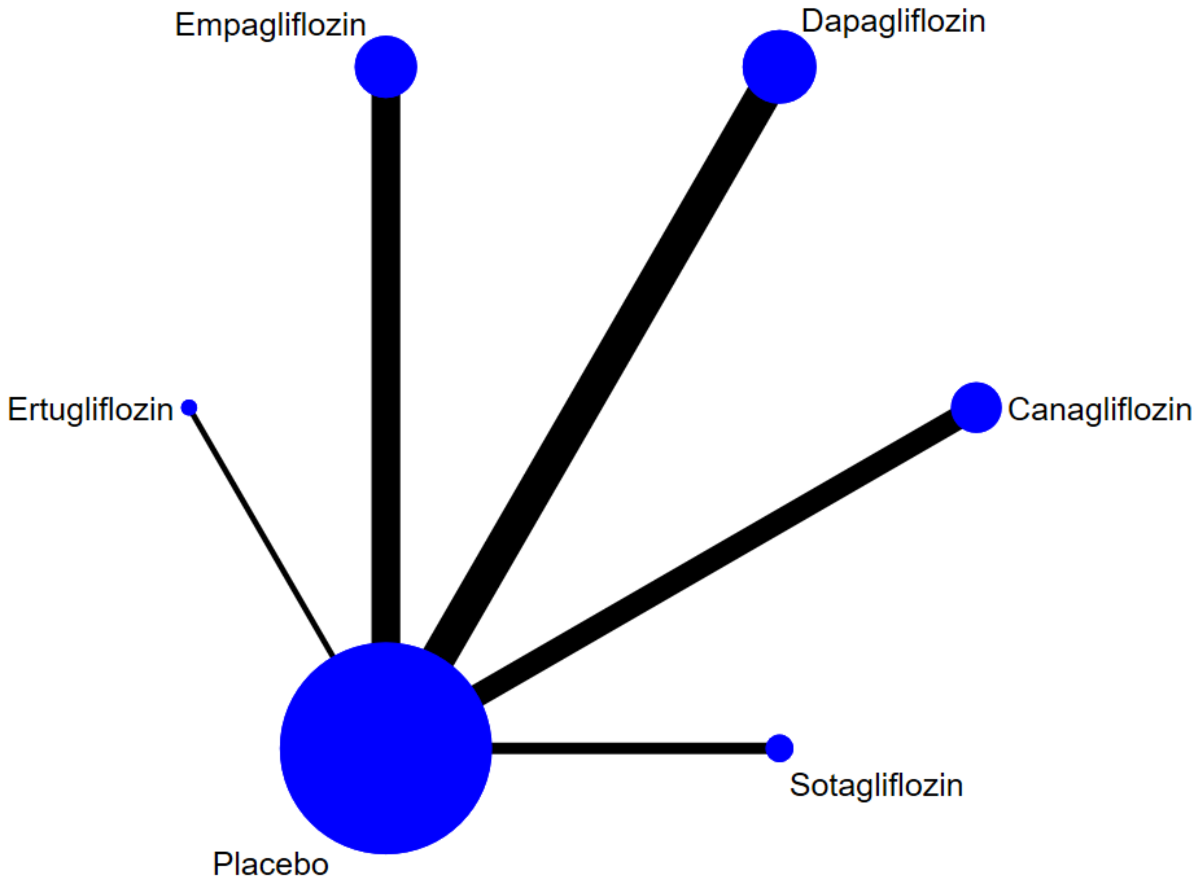

Figure S51 Network plot of *Hypertension*

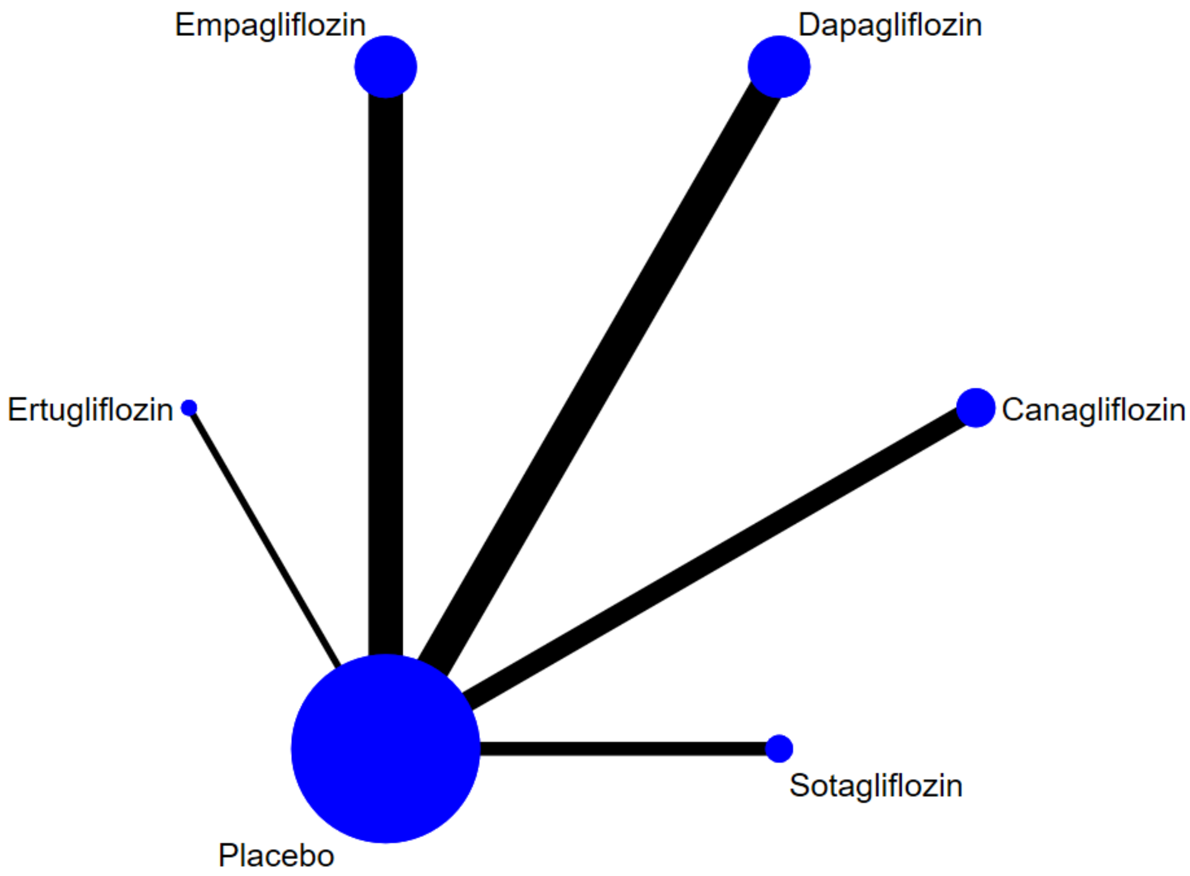

Figure S52 Network plot of *Acute respiratory failure*

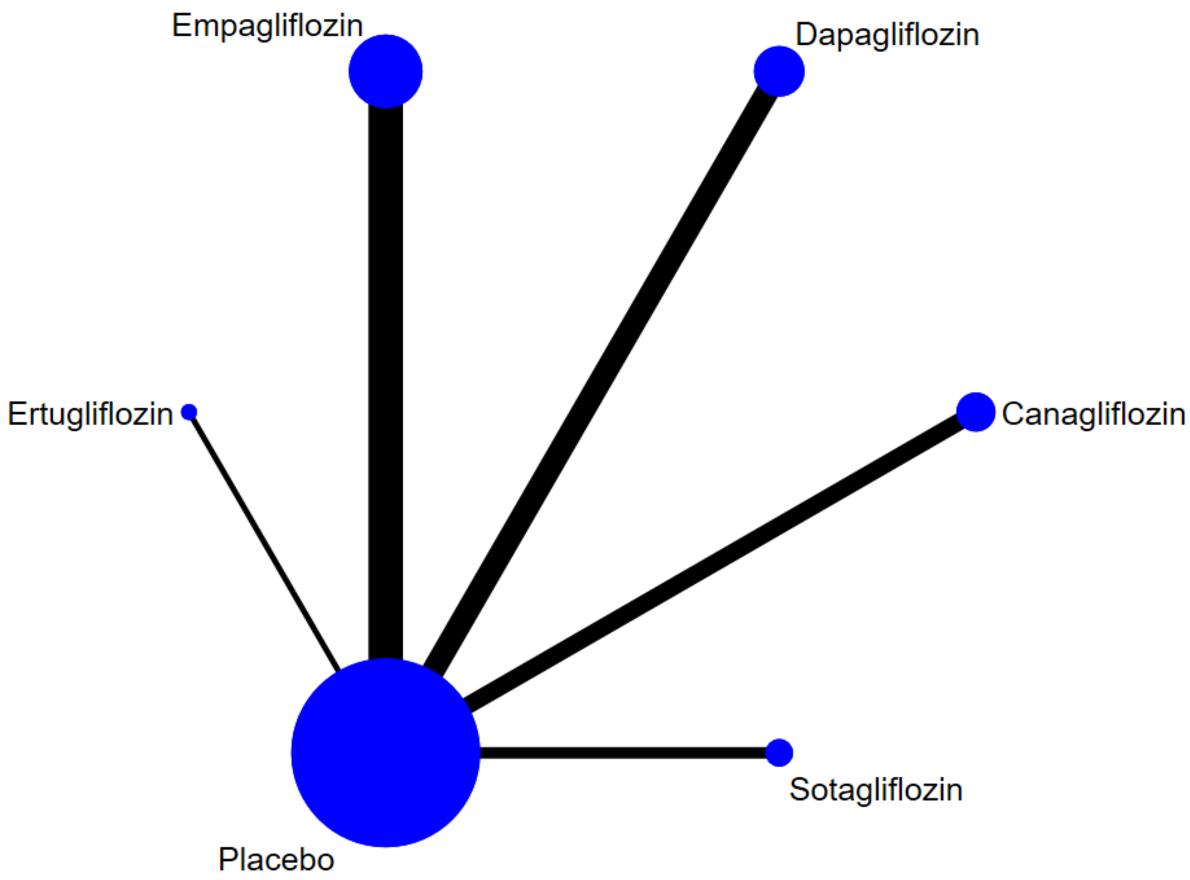

Figure S53 Network plot of *Pulmonary oedema*

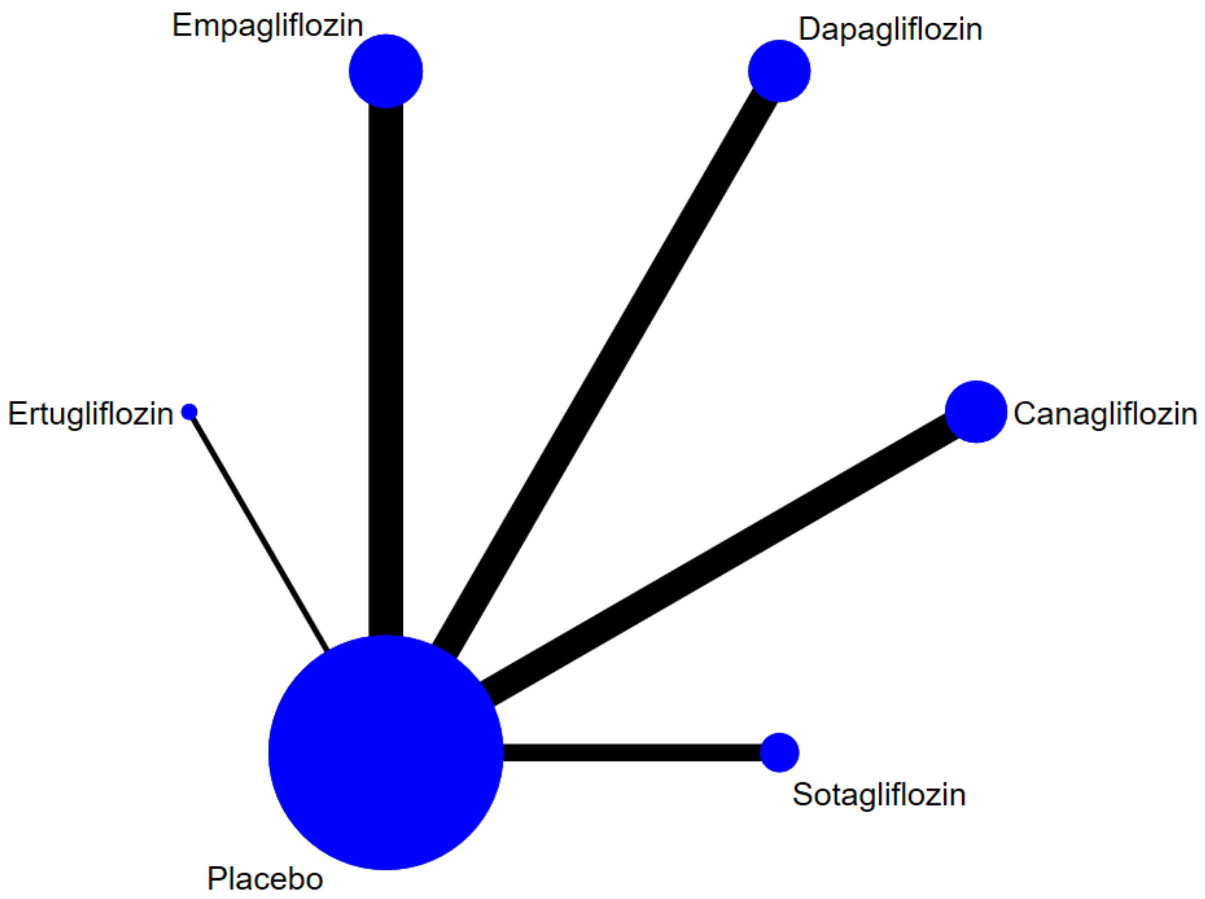

Figure S54 Network plot of *Chronic obstructive pulmonary disease*

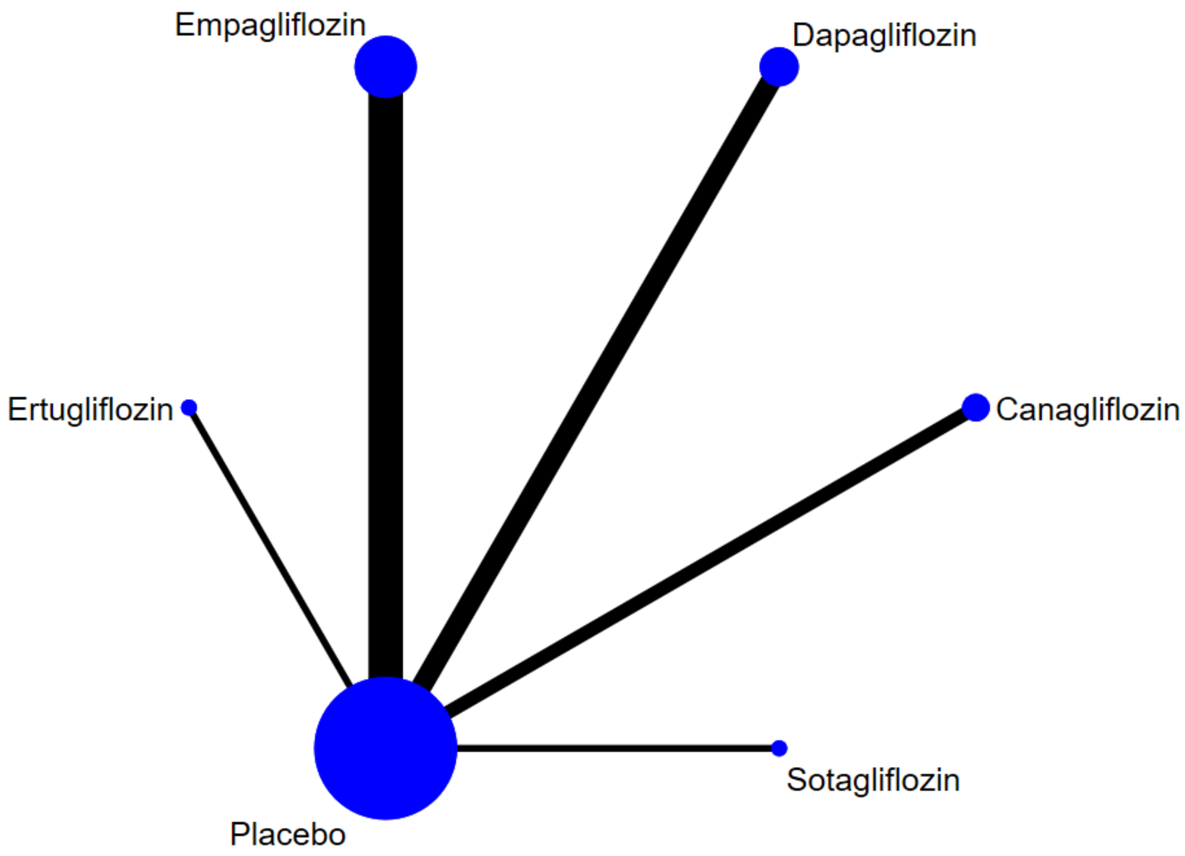

Figure S55 Network plot of *Pulmonary hypertension*

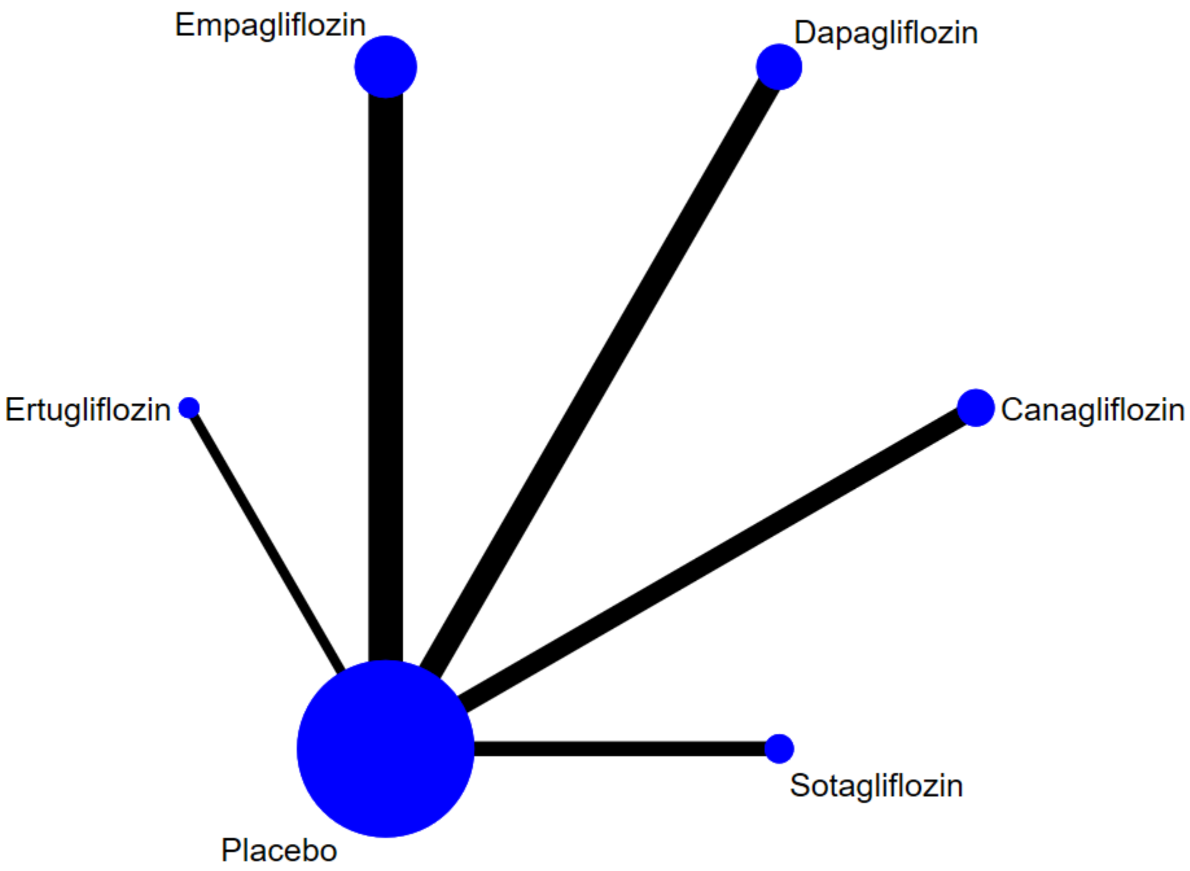

Figure S56 Network plot of *Dyspnoea*

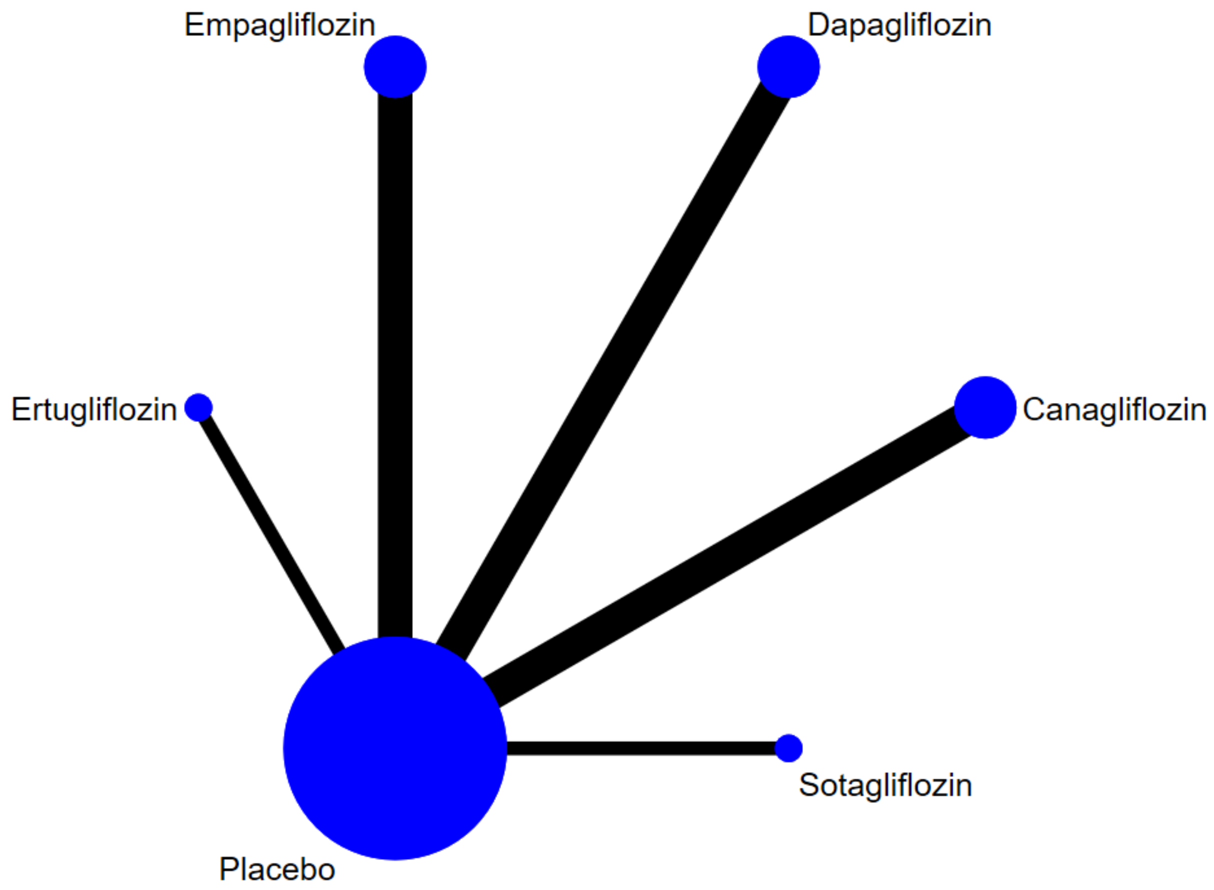

Figure S57 Network plot of *Asthma*

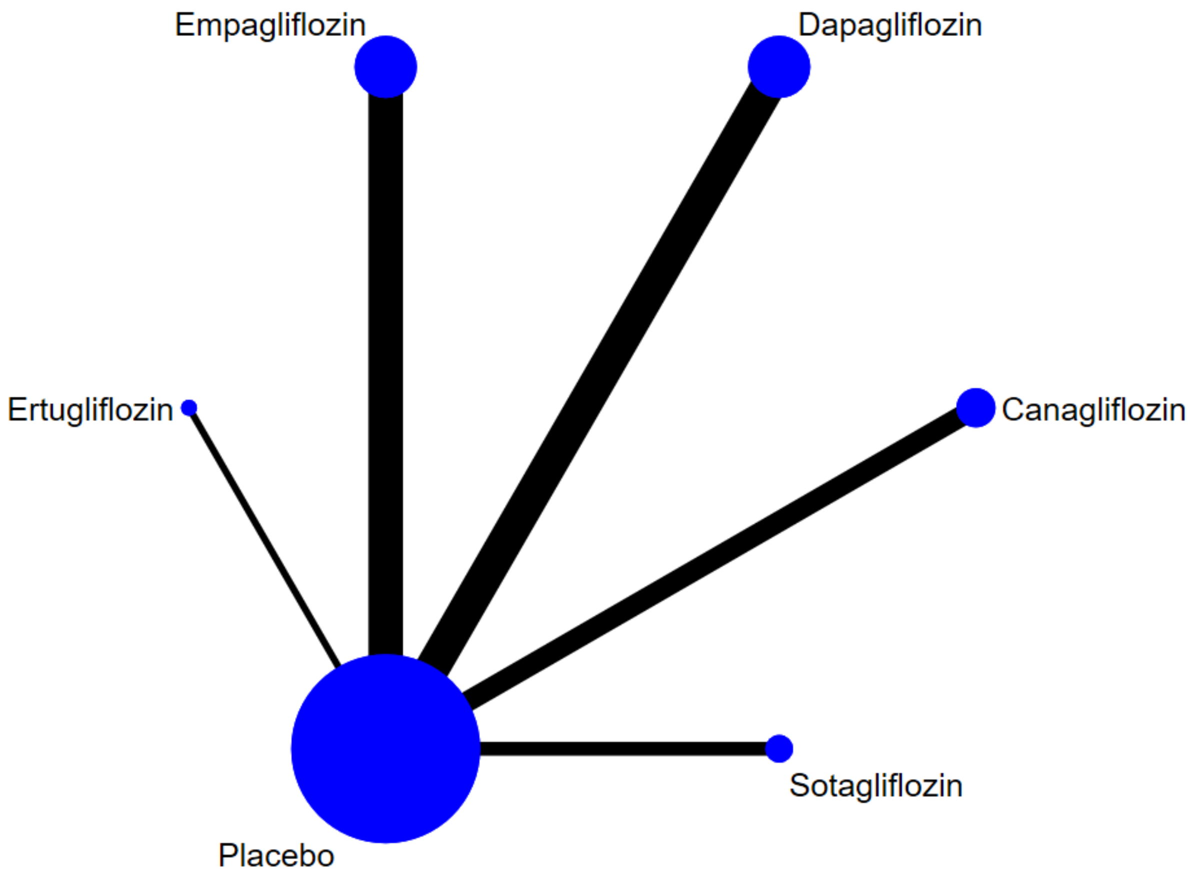

Figure S58 Network plot of *Respiratory tract infection*

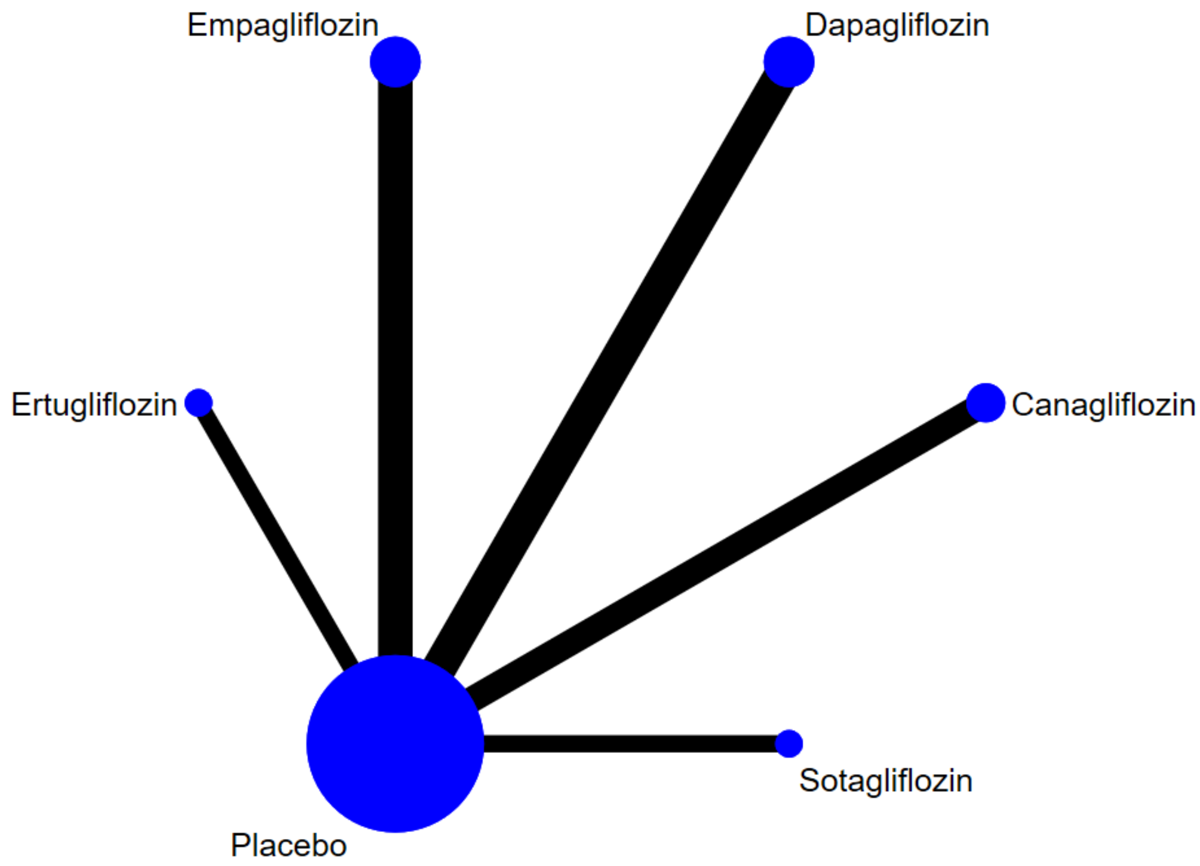

Figure S59 Network plot of *Lower respiratory tract infection*

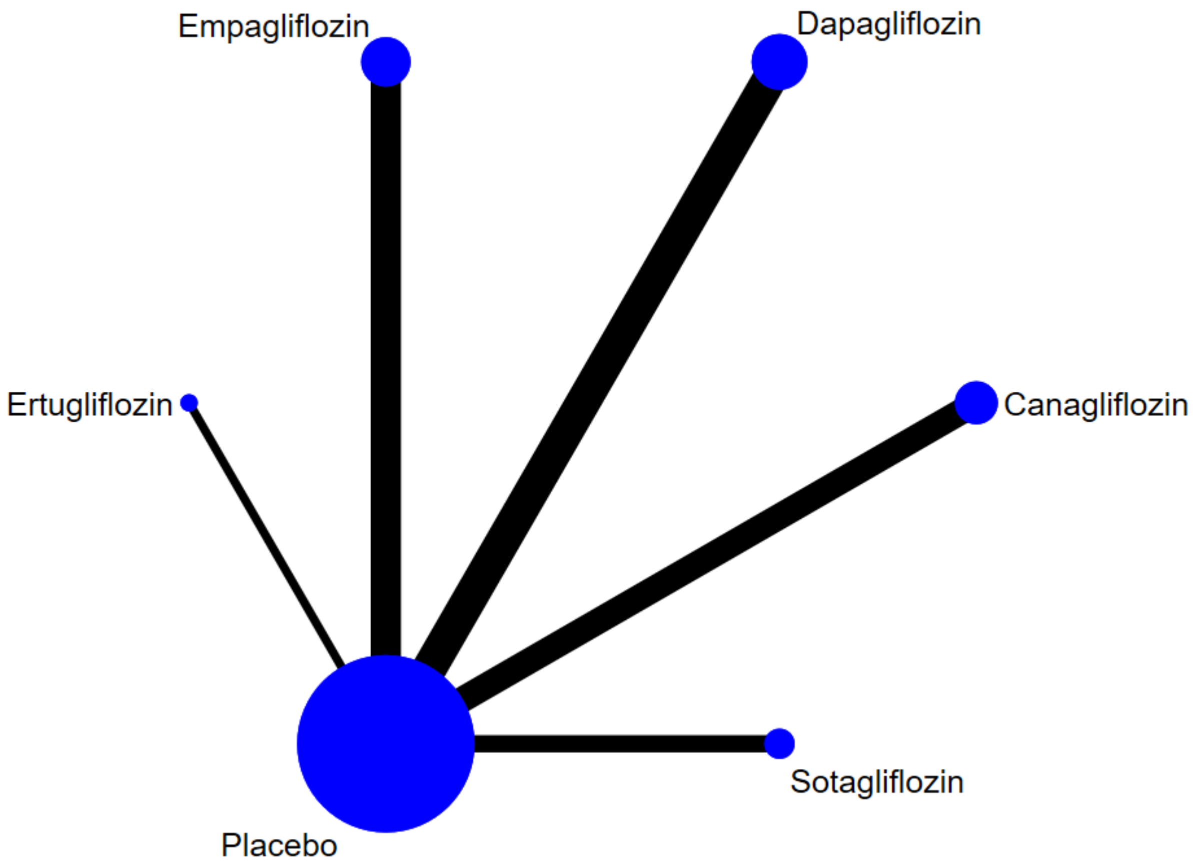

Figure S60 Network plot of *Pneumonia*

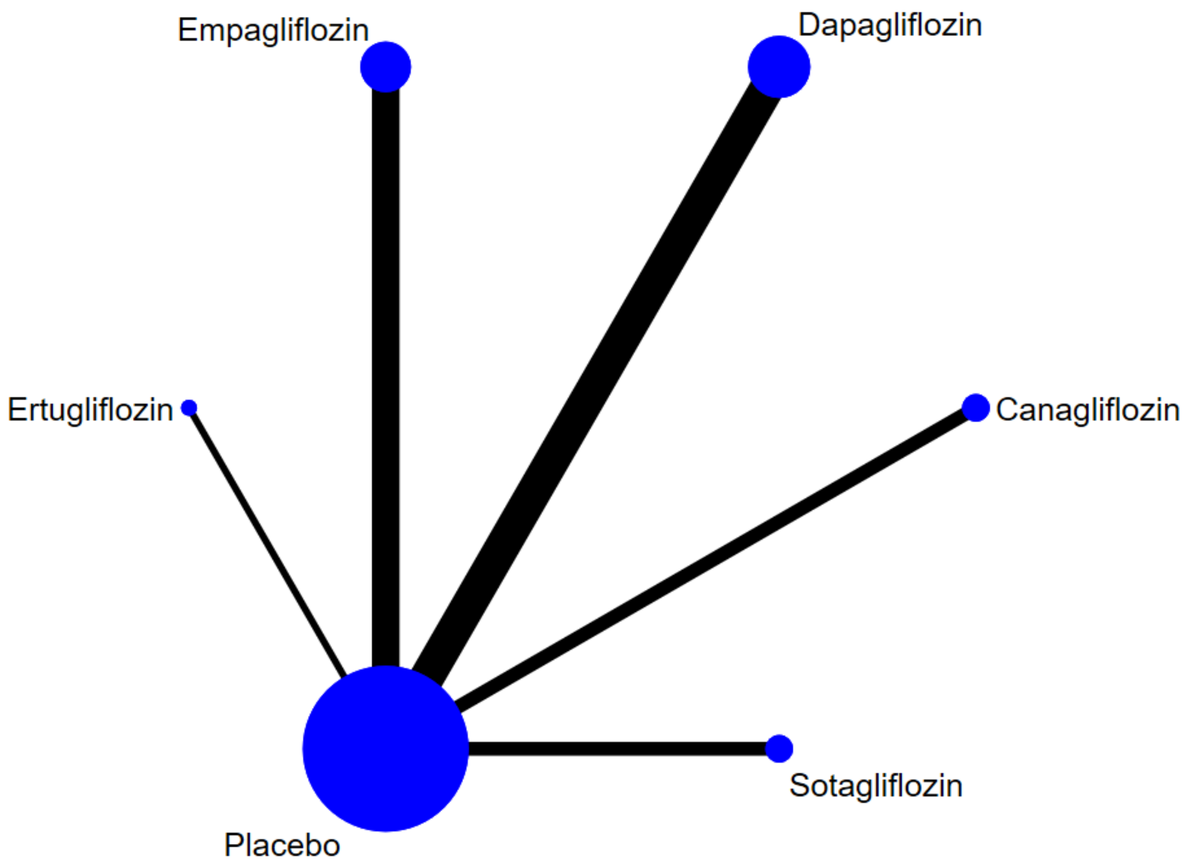

Figure S61 Network plot of *Pneumonia bacterial*

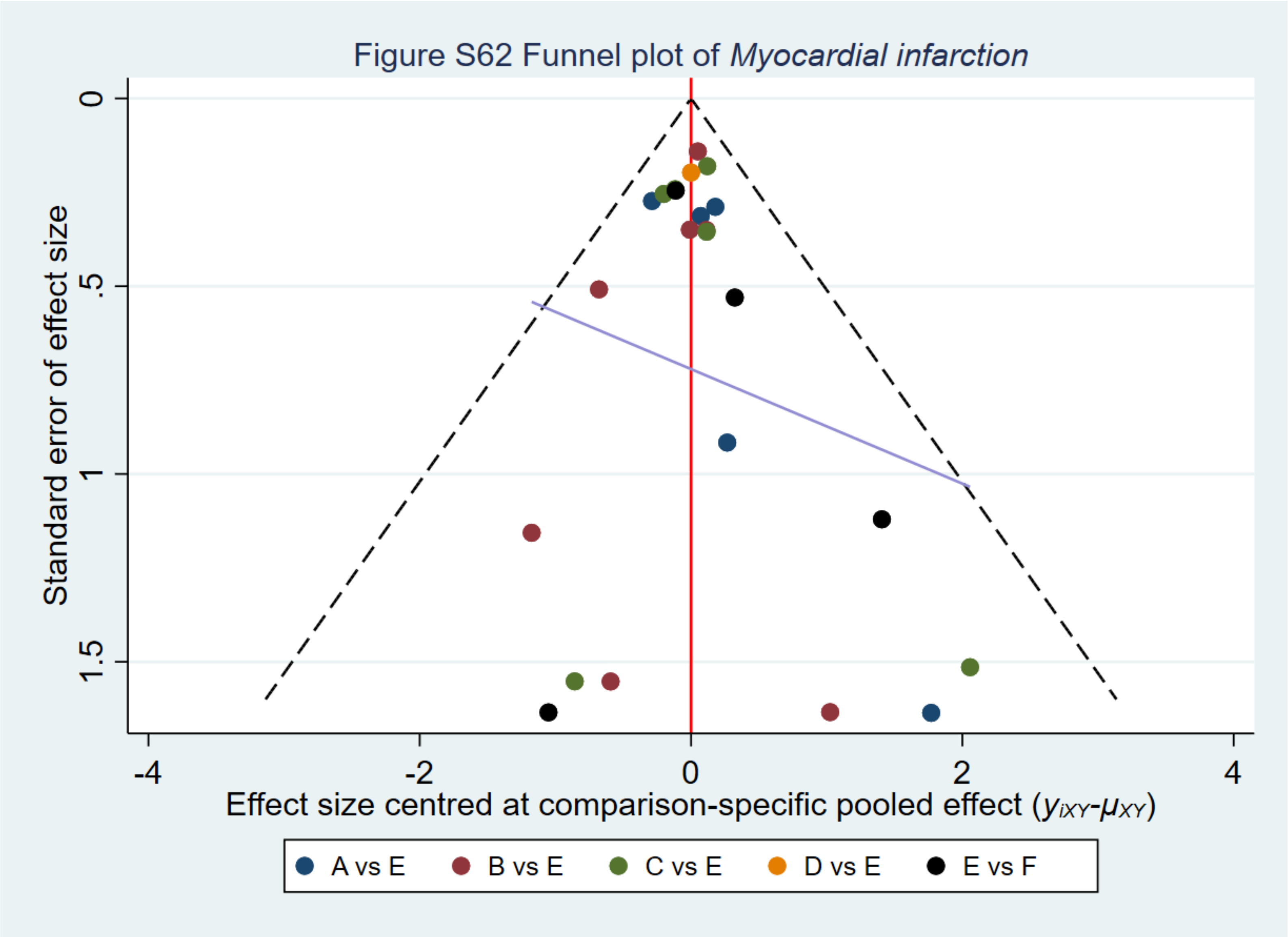
Figure S62 Funnel plot of *Myocardial infarction*
Standard error of effect size
0
.5
1
1.5
-4
-2
0
2
4
Effect size centred at comparison-specific pooled effect (y_{iXY}-μ_{XY})
A vs E
B vs E
C vs E
D vs E
E vs F

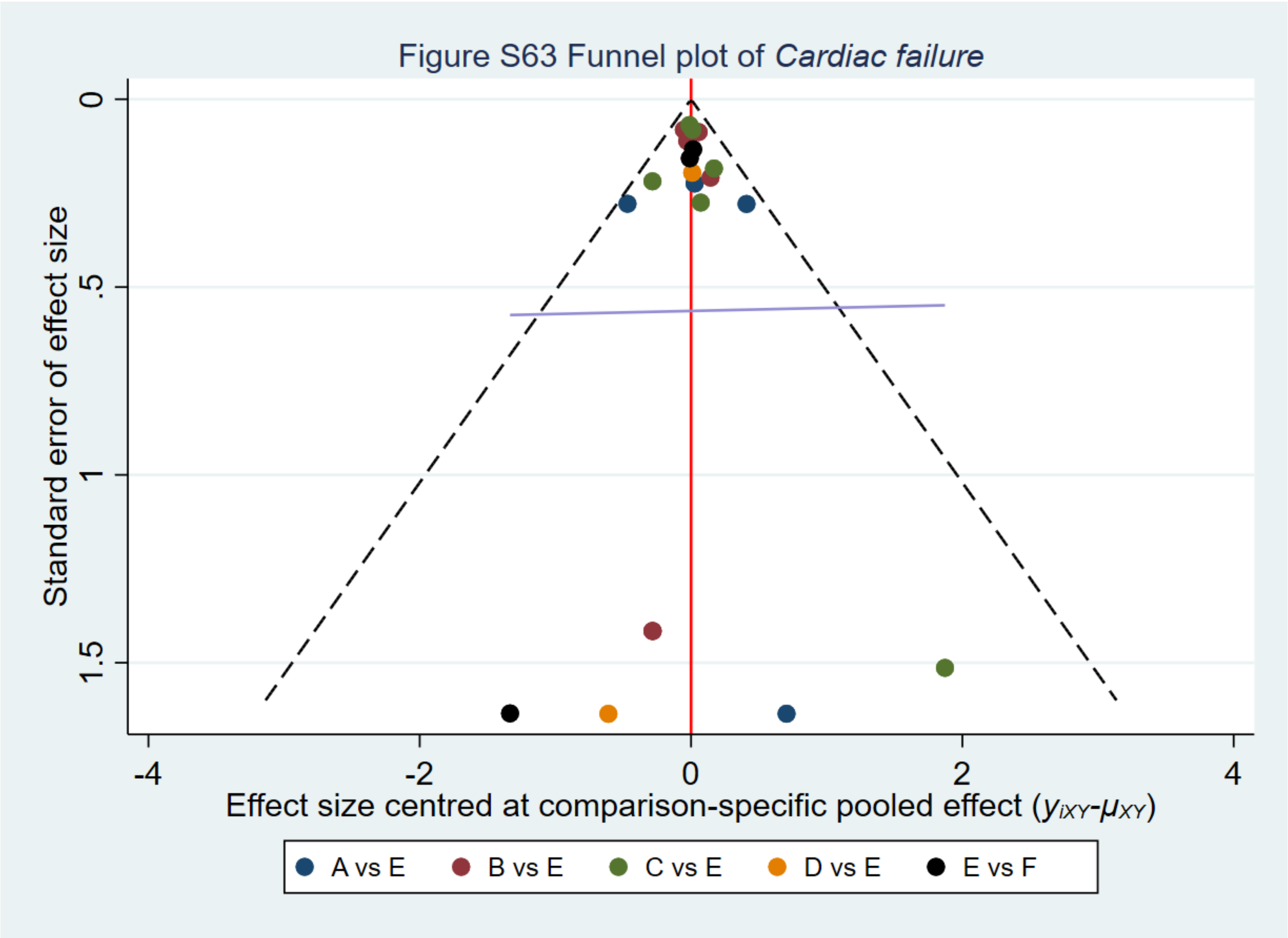

Figure S63 Funnel plot of Cardiac failure
Standard error of effect size
0
.5
1
1.5
-4
-2
0
2
4
Effect size centred at comparison-specific pooled effect (y_{iXY}-μ_{XY})
A vs E
B vs E
C vs E
D vs E
E vs F

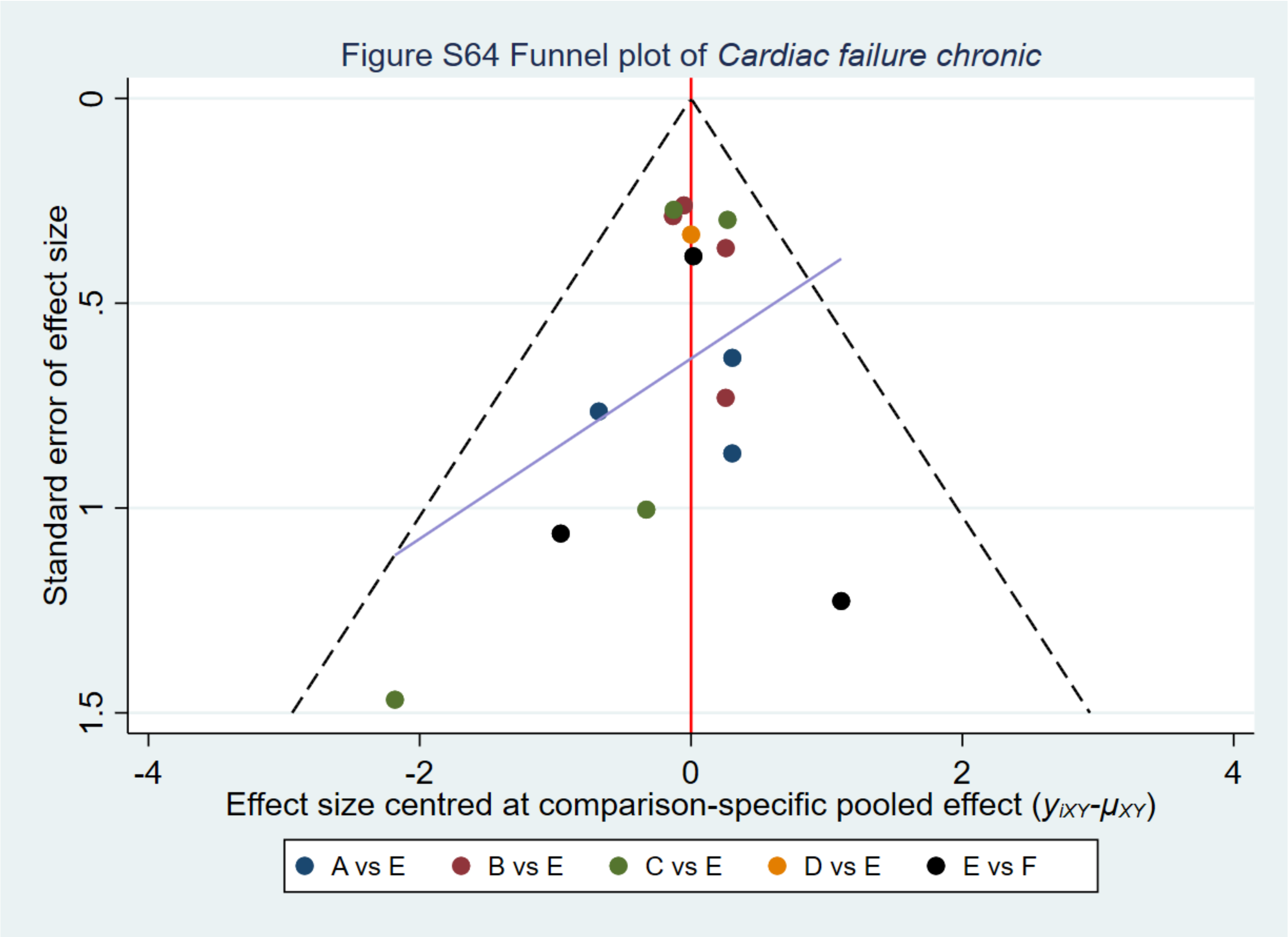
Figure S64 Funnel plot of *Cardiac failure chronic*
Standard error of effect size
0
.5
1
1.5
-4
-2
0
2
4
Effect size centred at comparison-specific pooled effect (y_{iXY}-μ_{XY})
A vs E
B vs E
C vs E
D vs E
E vs F

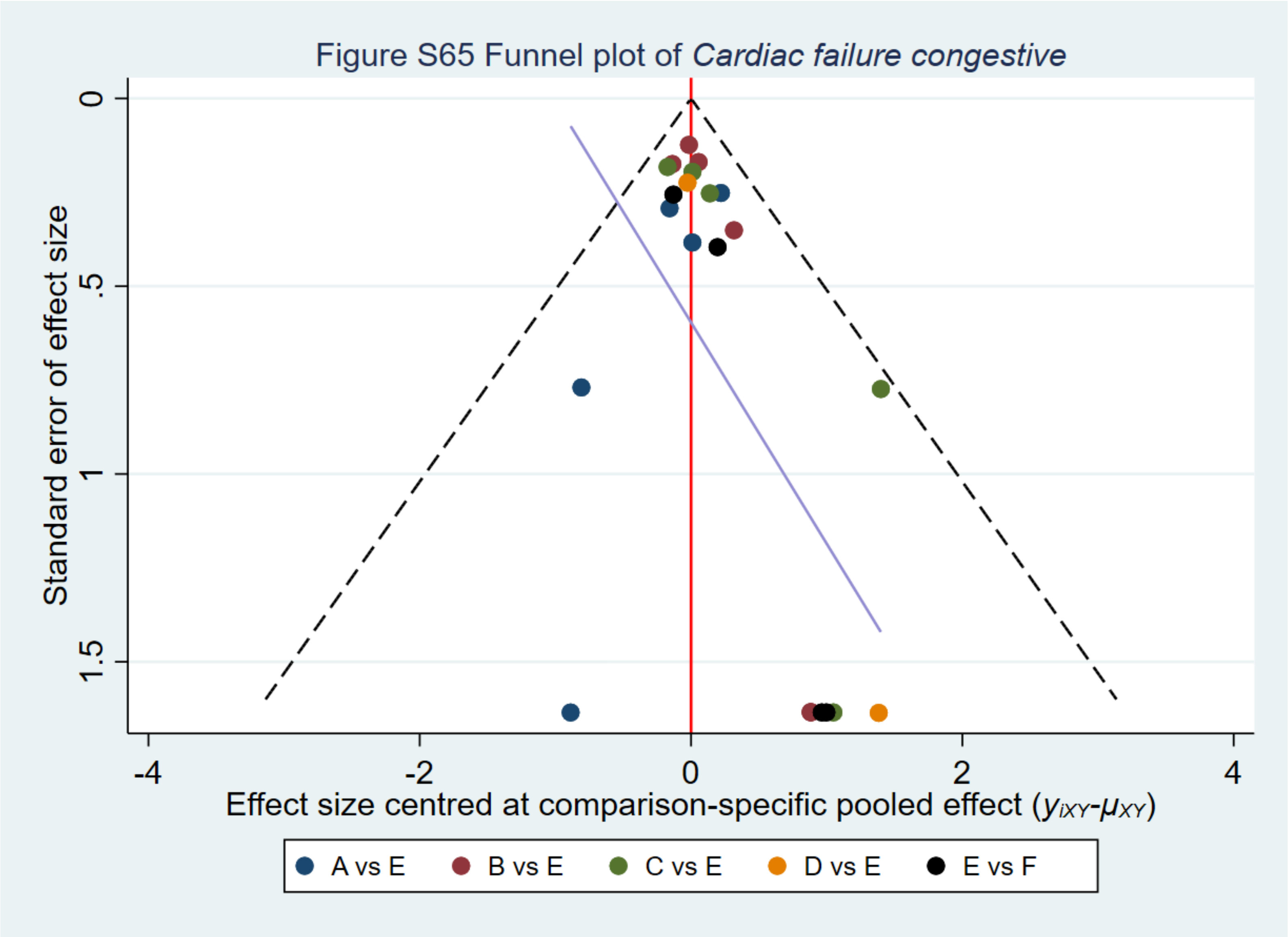
Figure S65 Funnel plot of Cardiac failure congestive
Standard error of effect size
0
.5
1
1.5
-4
-2
0
2
4
Effect size centred at comparison-specific pooled effect (y_{iXY}-μ_{XY})
A vs E
B vs E
C vs E
D vs E
E vs F

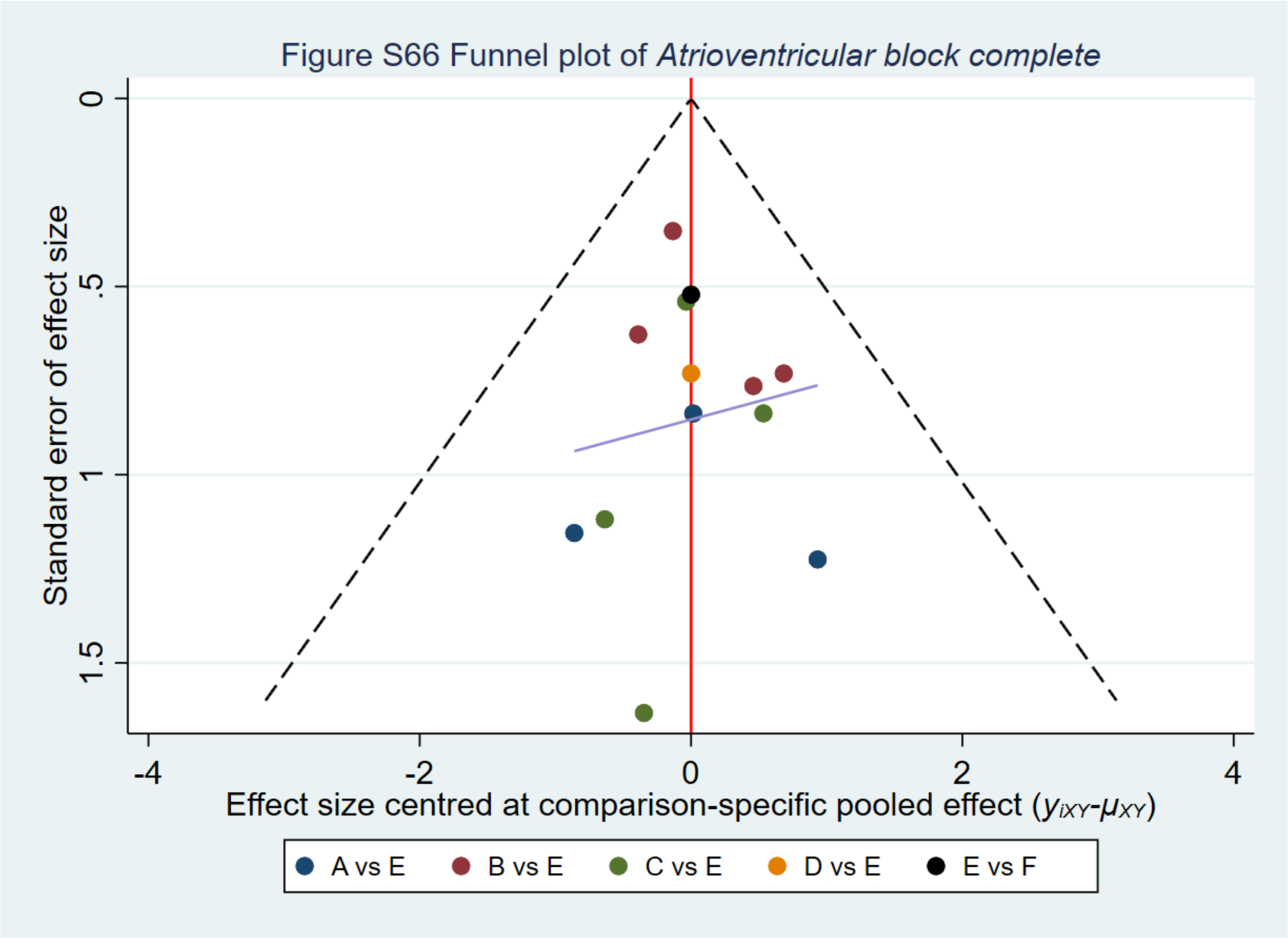

Figure S66 Funnel plot of *Atrioventricular block complete*
Standard error of effect size
0
.5
1
1.5
-4
-2
0
2
4
Effect size centred at comparison-specific pooled effect (y_{iXY}-μ_{XY})
A vs E
B vs E
C vs E
D vs E
E vs F

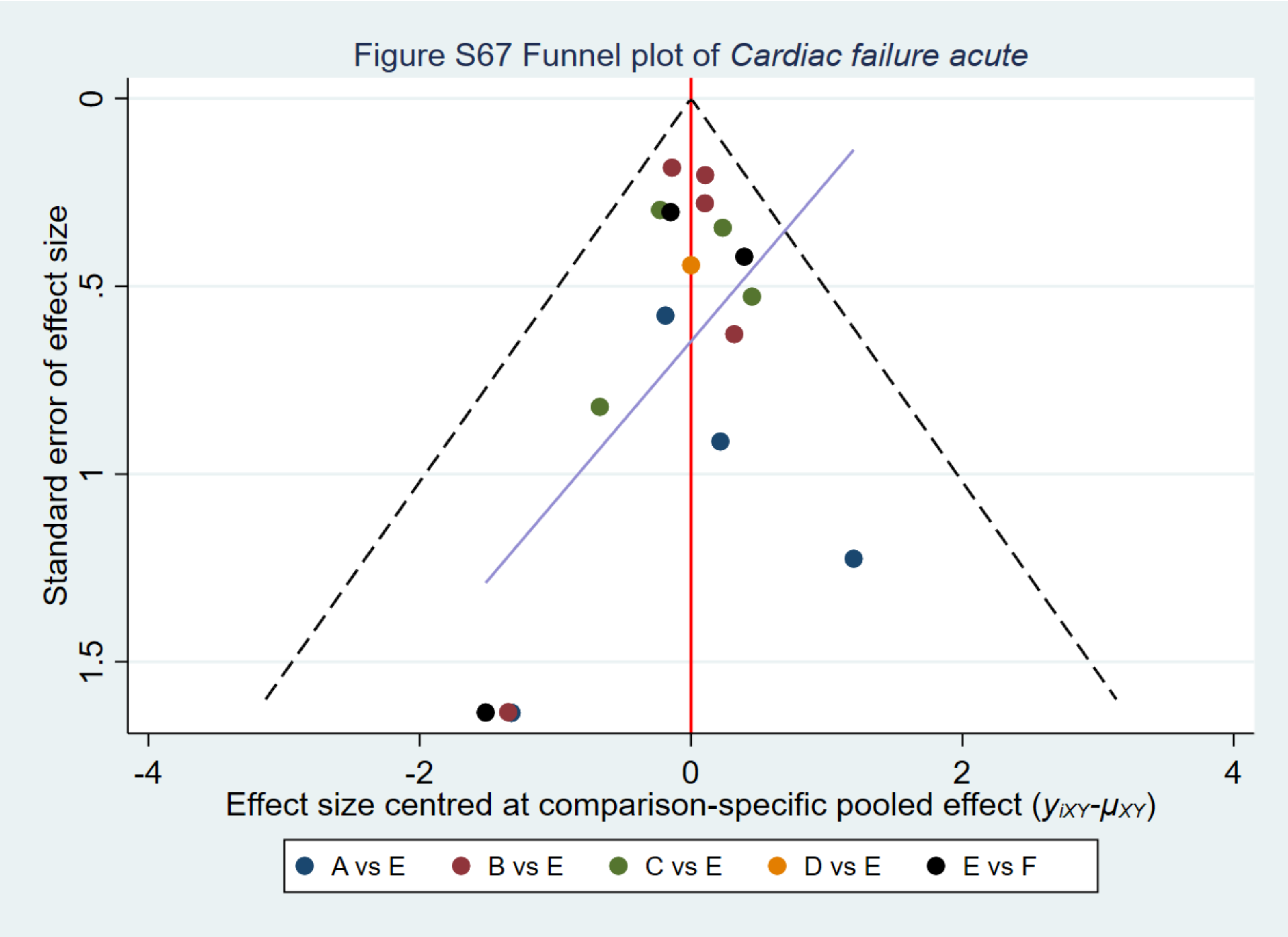

Figure S67 Funnel plot of *Cardiac failure acute*
Standard error of effect size
0
.5
1
1.5
-4
-2
0
2
4
Effect size centred at comparison-specific pooled effect ($y_{iXY}-\mu_{XY}$)
A vs E
B vs E
C vs E
D vs E
E vs F

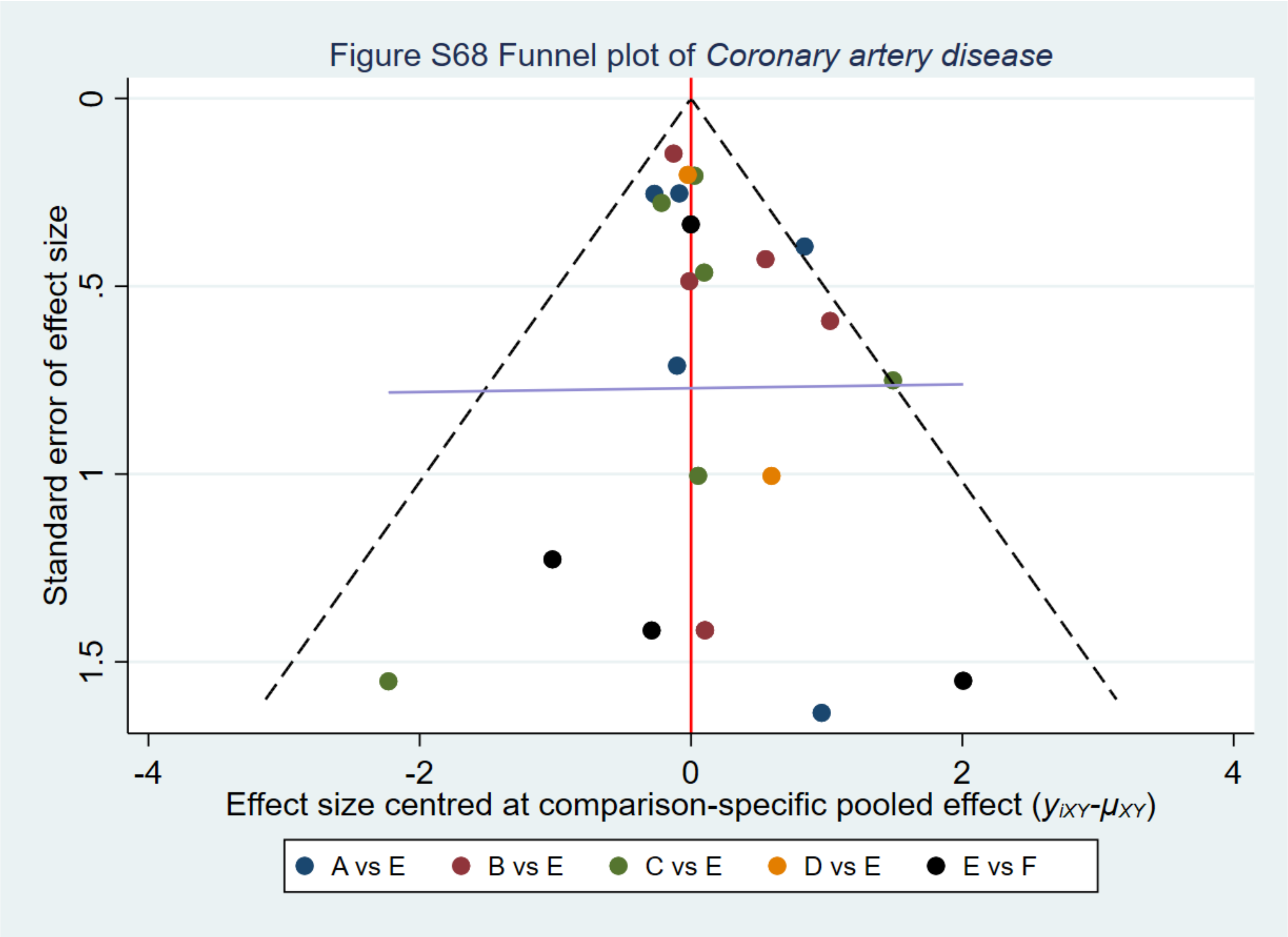

Figure S68 Funnel plot of *Coronary artery disease*
Standard error of effect size
0
.5
1
1.5
-4
-2
0
2
4
Effect size centred at comparison-specific pooled effect (y_{iXY}-μ_{XY})
A vs E
B vs E
C vs E
D vs E
E vs F

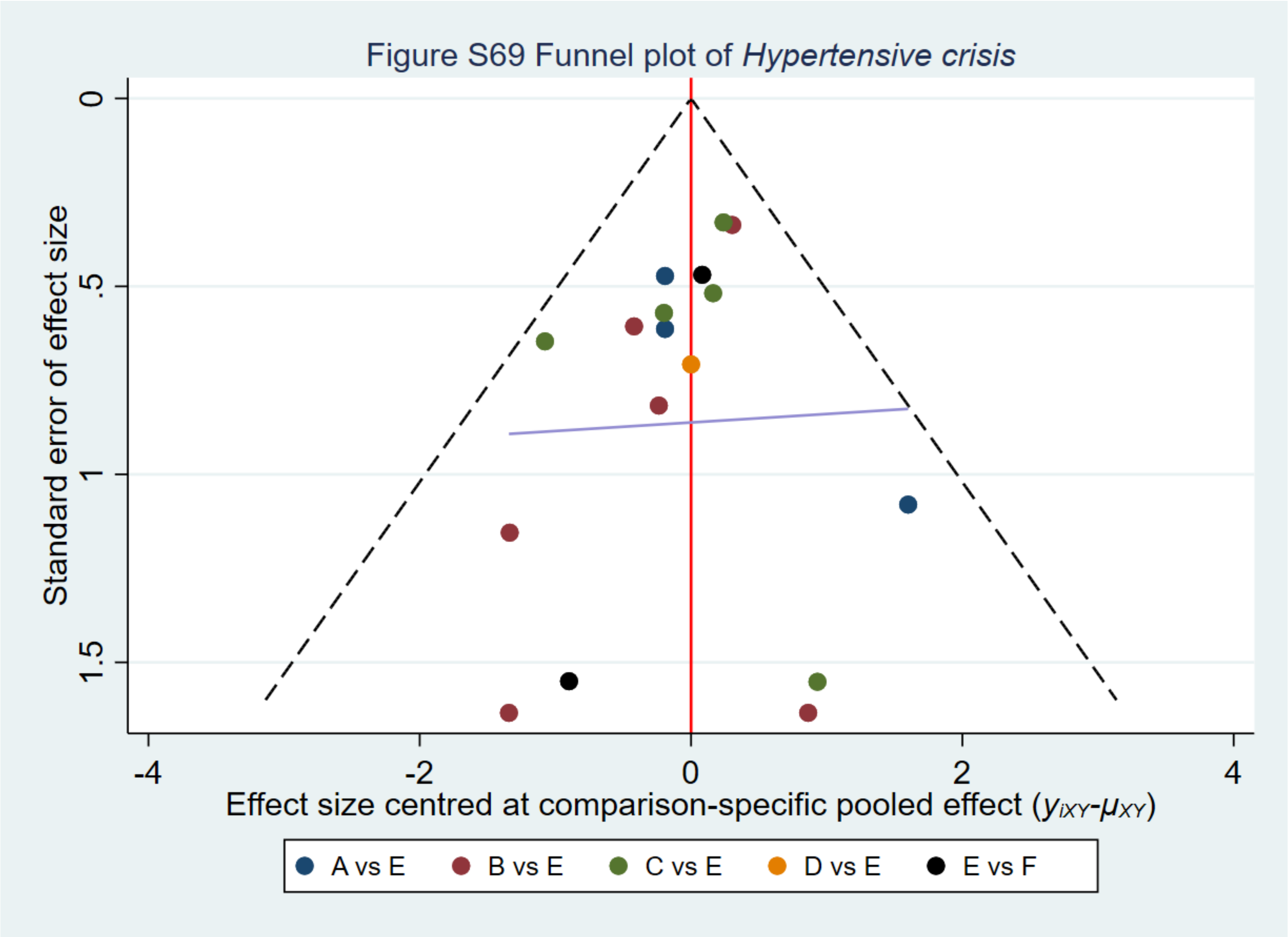
Figure S69 Funnel plot of Hypertensive crisis
Standard error of effect size
0
.5
1
1.5
-4
-2
0
2
4
Effect size centred at comparison-specific pooled effect (yiXY-μXY)
A vs E
B vs E
C vs E
D vs E
E vs F

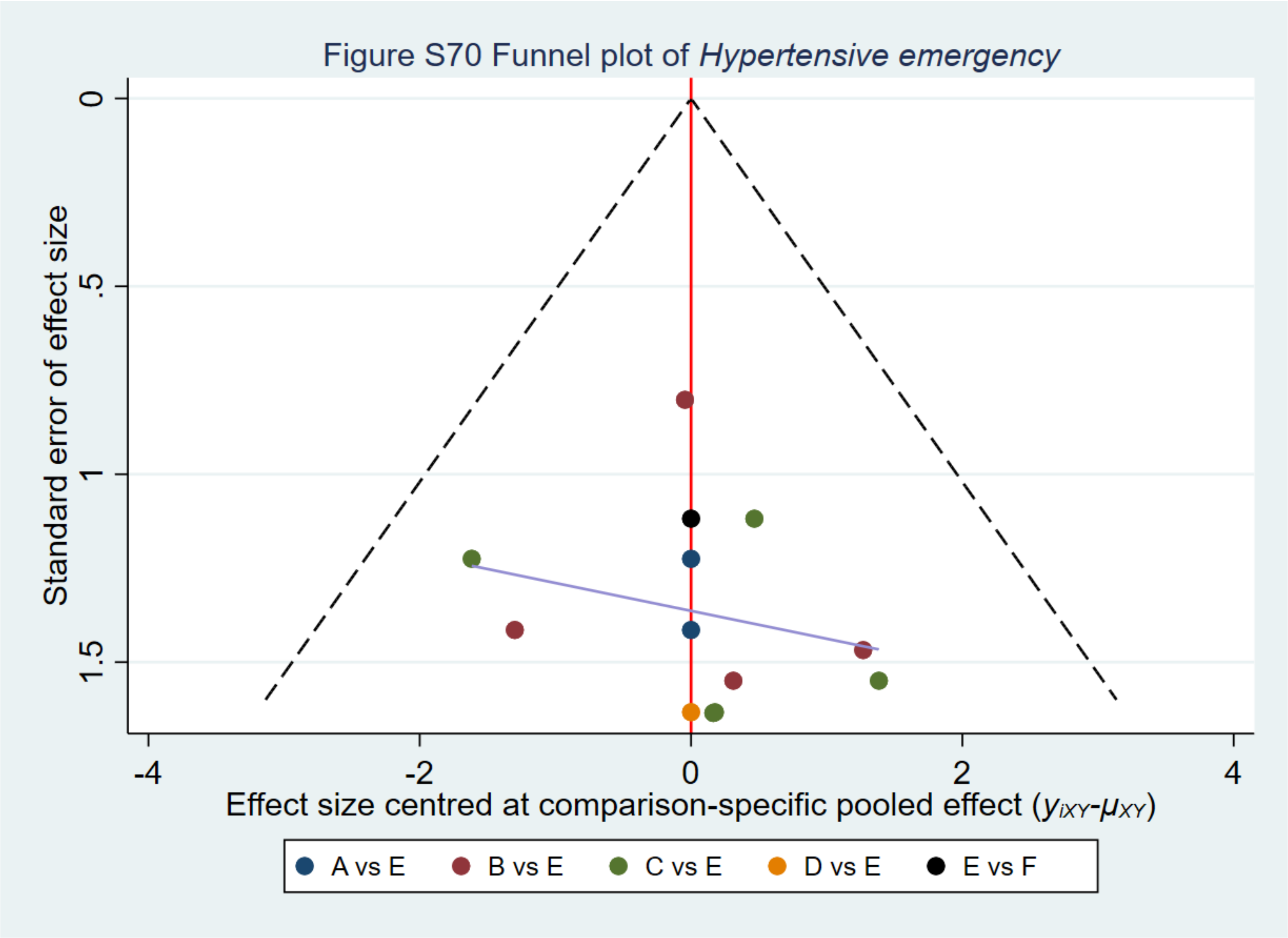
Figure S70 Funnel plot of Hypertensive emergency
Standard error of effect size
0
.5
1
1.5
-4
-2
0
2
4
Effect size centred at comparison-specific pooled effect (y_{iXY}-μ_{XY})
A vs E
B vs E
C vs E
D vs E
E vs F

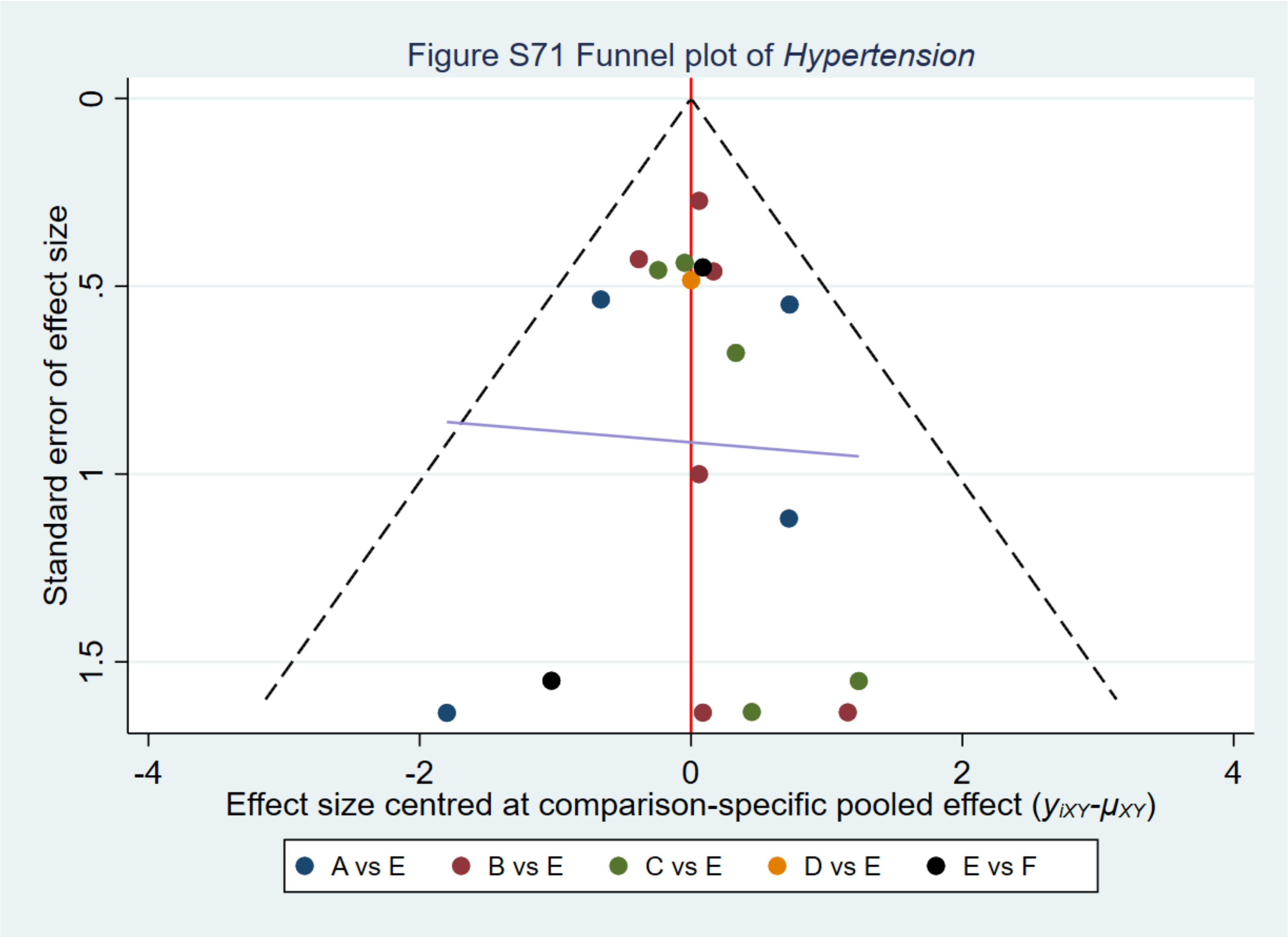
Figure S71 Funnel plot of Hypertension
Standard error of effect size
0
.5
1
1.5
-4
-2
0
2
4
Effect size centred at comparison-specific pooled effect (y_{iXY}-μ_{XY})
A vs E
B vs E
C vs E
D vs E
E vs F

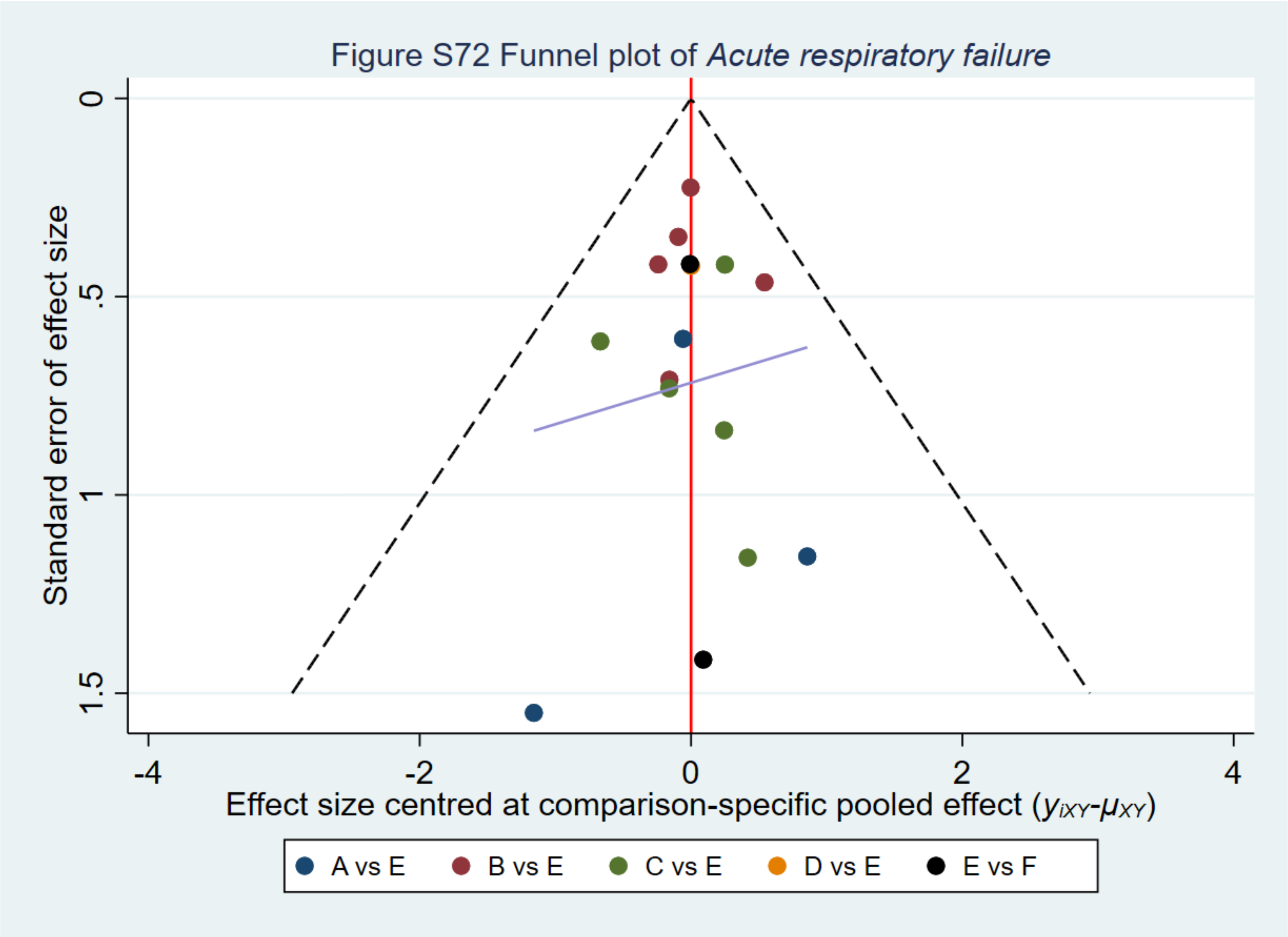

Figure S72 Funnel plot of *Acute respiratory failure*
Standard error of effect size
0
.5
1
1.5
-4
-2
0
2
4
Effect size centred at comparison-specific pooled effect ($y_{iXY}-\mu_{XY}$)
A vs E
B vs E
C vs E
D vs E
E vs F

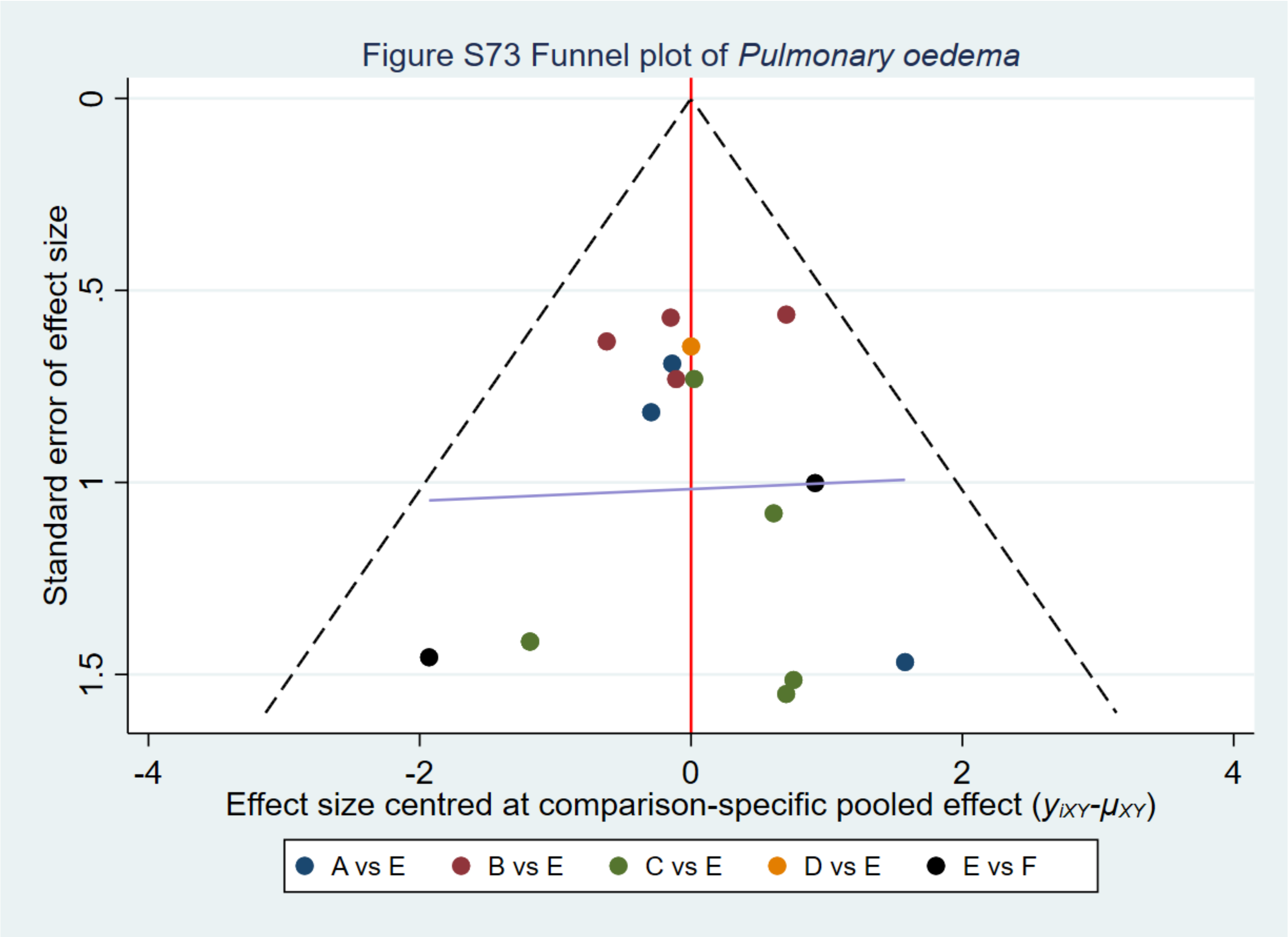
Figure S73 Funnel plot of *Pulmonary oedema*
Standard error of effect size
0
.5
1
1.5
-4
-2
0
2
4
Effect size centred at comparison-specific pooled effect (y_{iXY}-μ_{XY})
A vs E
B vs E
C vs E
D vs E
E vs F

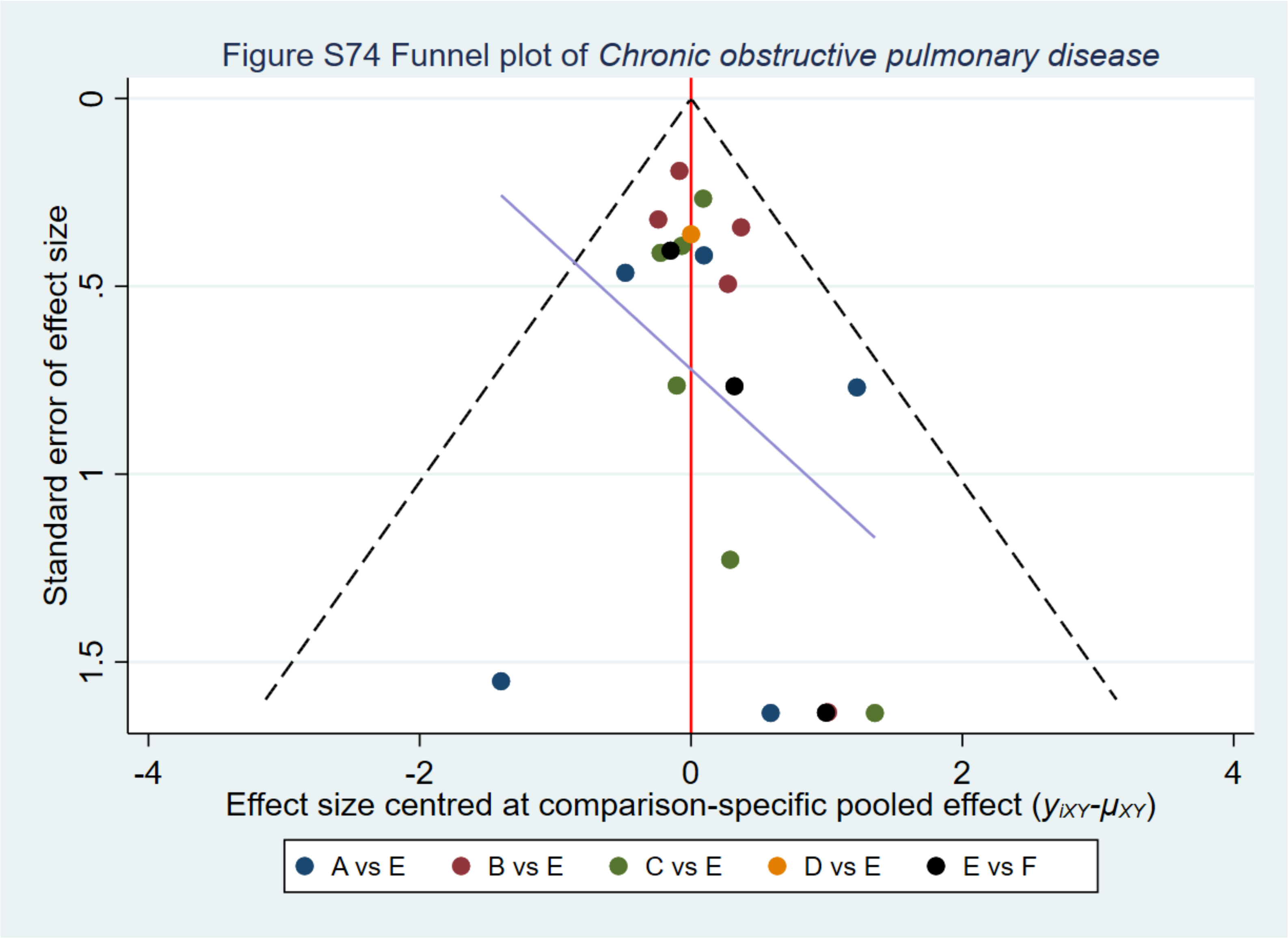
Figure S74 Funnel plot of *Chronic obstructive pulmonary disease*
Standard error of effect size
0
.5
1
1.5
-4
-2
0
2
4
Effect size centred at comparison-specific pooled effect (y_{iXY}-μ_{XY})
A vs E
B vs E
C vs E
D vs E
E vs F

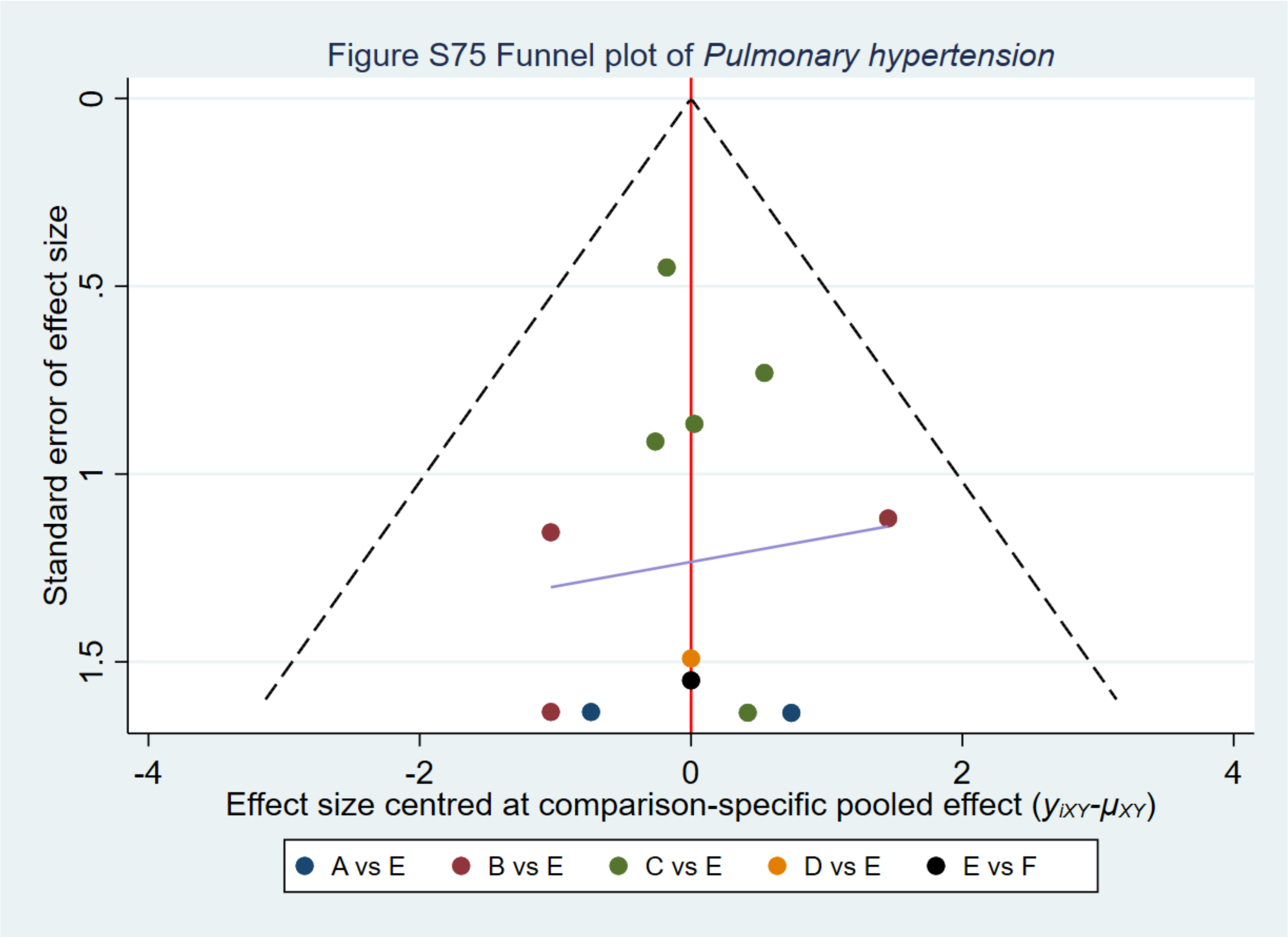
Figure S75 Funnel plot of *Pulmonary hypertension*
Standard error of effect size
0
.5
1
1.5
-4
-2
0
2
4
Effect size centred at comparison-specific pooled effect (y_{iXY}-μ_{XY})
A vs E
B vs E
C vs E
D vs E
E vs F

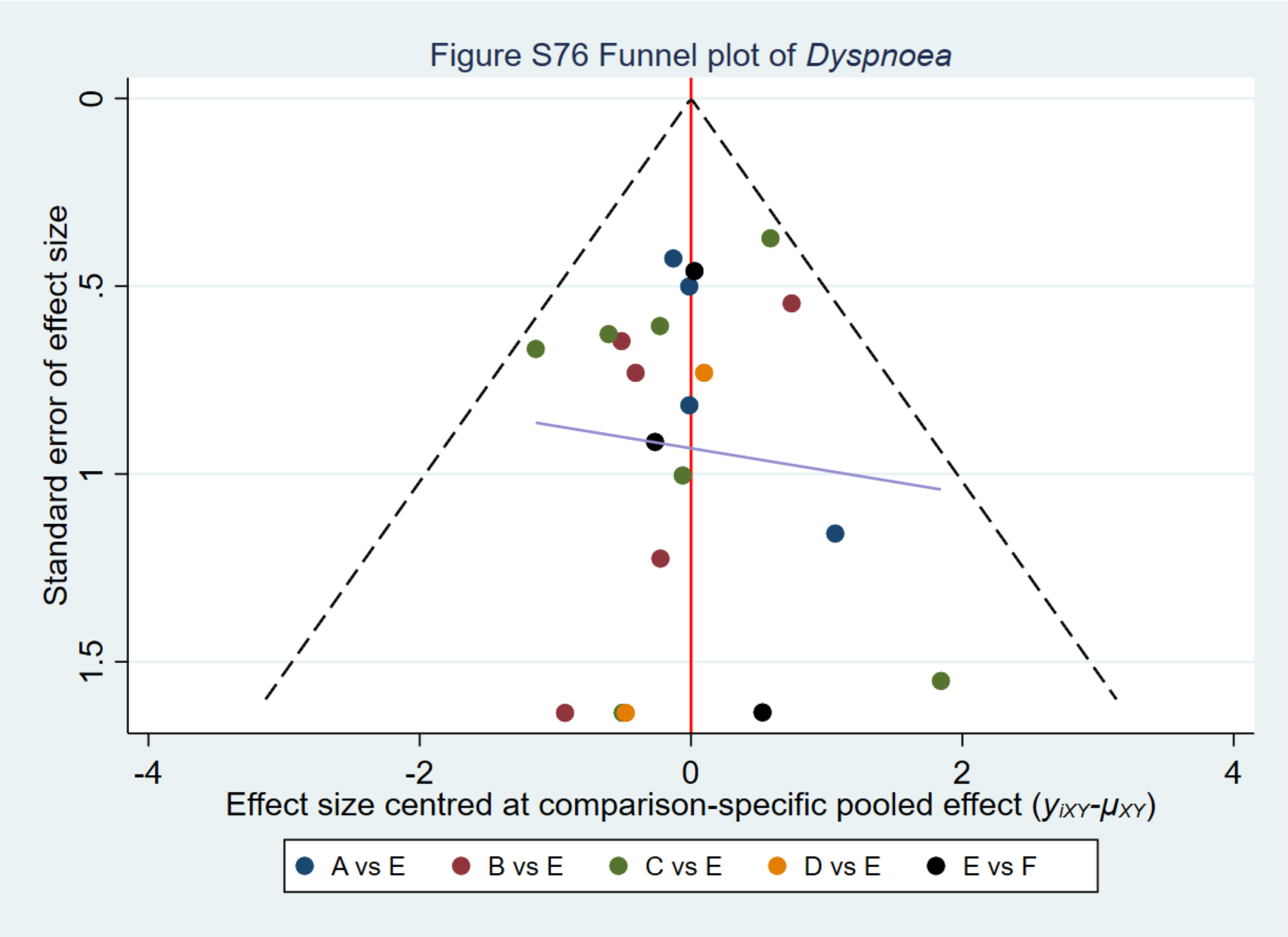

Figure S76 Funnel plot of *Dyspnoea*
Standard error of effect size
0
.5
1
1.5
-4
-2
0
2
4
Effect size centred at comparison-specific pooled effect (y_{iXY}-μ_{XY})
A vs E
B vs E
C vs E
D vs E
E vs F

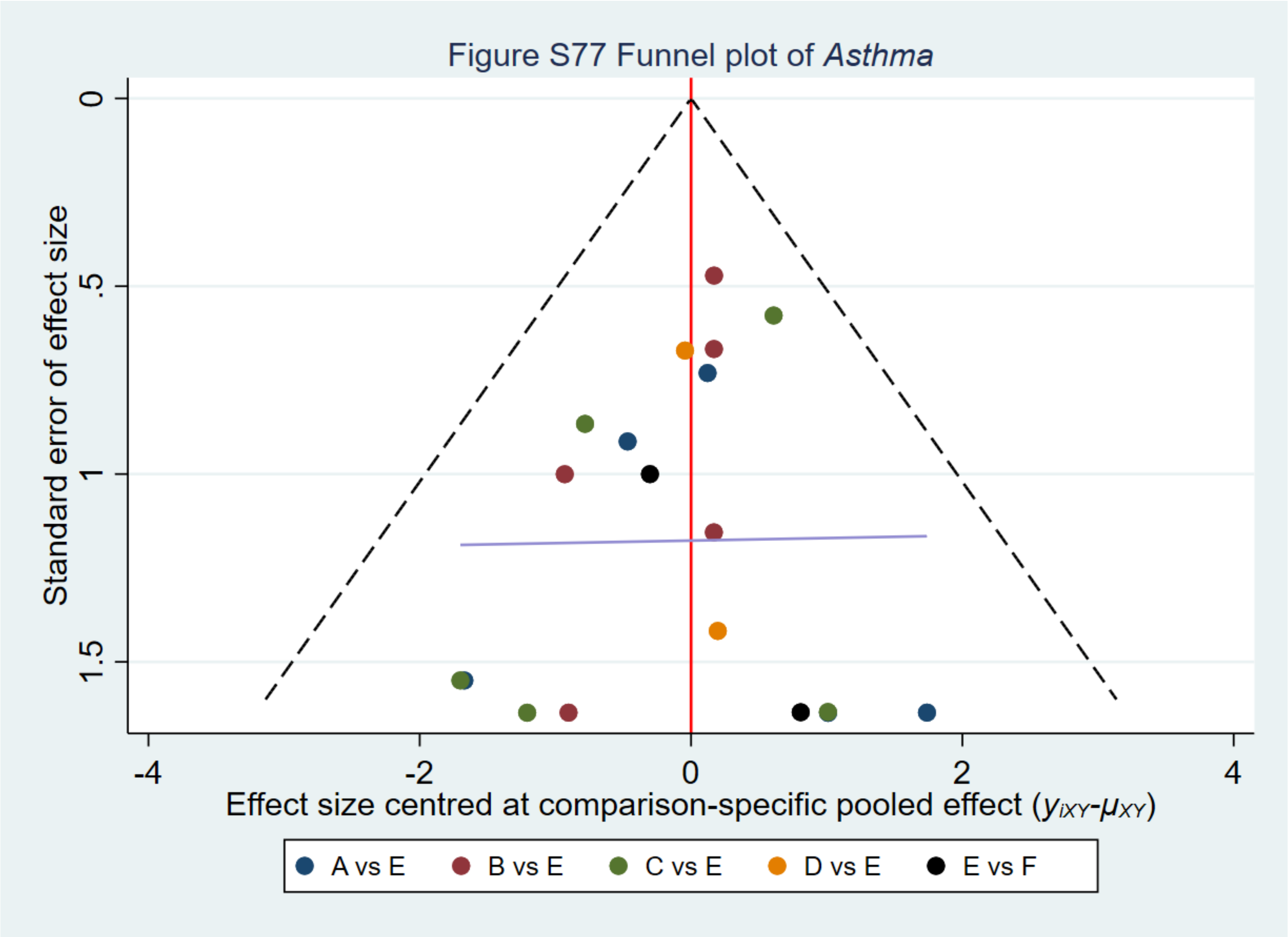

Figure S77 Funnel plot of *Asthma*
Standard error of effect size
0
.5
1
1.5
-4
-2
0
2
4
Effect size centred at comparison-specific pooled effect (y_{iXY}-μ_{XY})
A vs E
B vs E
C vs E
D vs E
E vs F

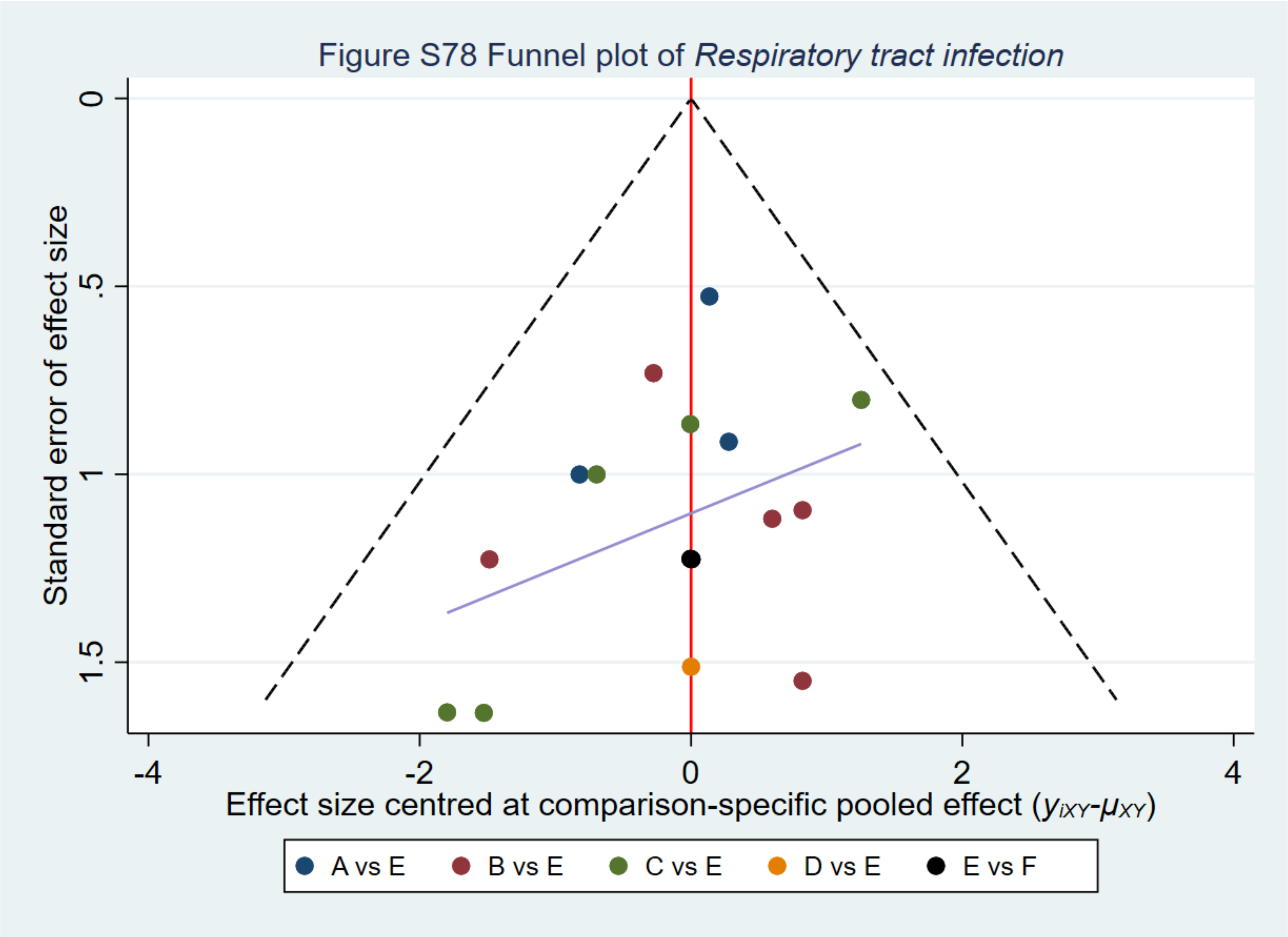
Figure S78 Funnel plot of Respiratory tract infection
Standard error of effect size
0
.5
1
1.5
-4
-2
0
2
4
Effect size centred at comparison-specific pooled effect (yiXY-μXY)
A vs E
B vs E
C vs E
D vs E
E vs F

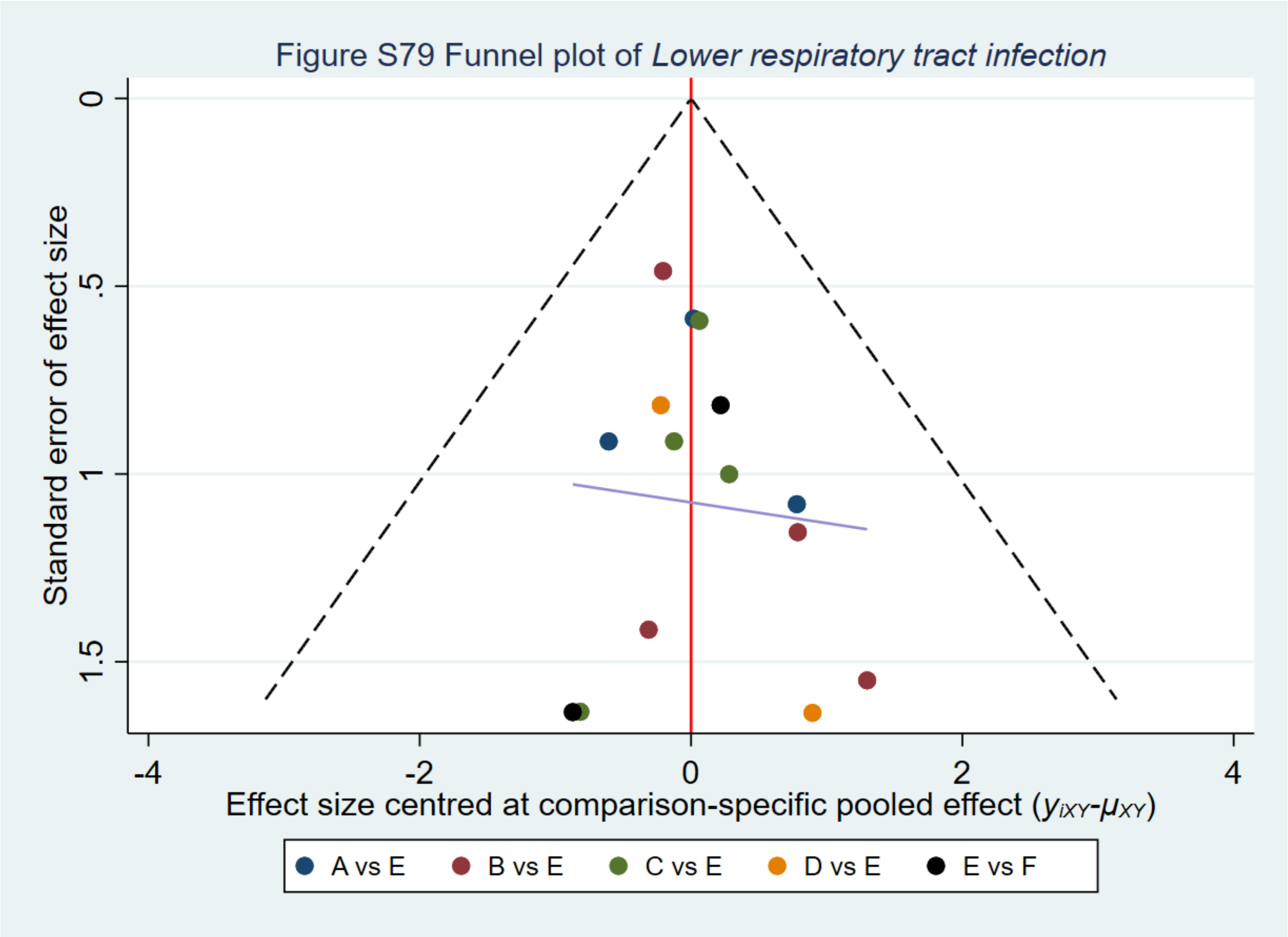

Figure S79 Funnel plot of *Lower respiratory tract infection*
Standard error of effect size
0
.5
1
1.5
-4
-2
0
2
4
Effect size centred at comparison-specific pooled effect (y_{iXY}-μ_{XY})
A vs E
B vs E
C vs E
D vs E
E vs F

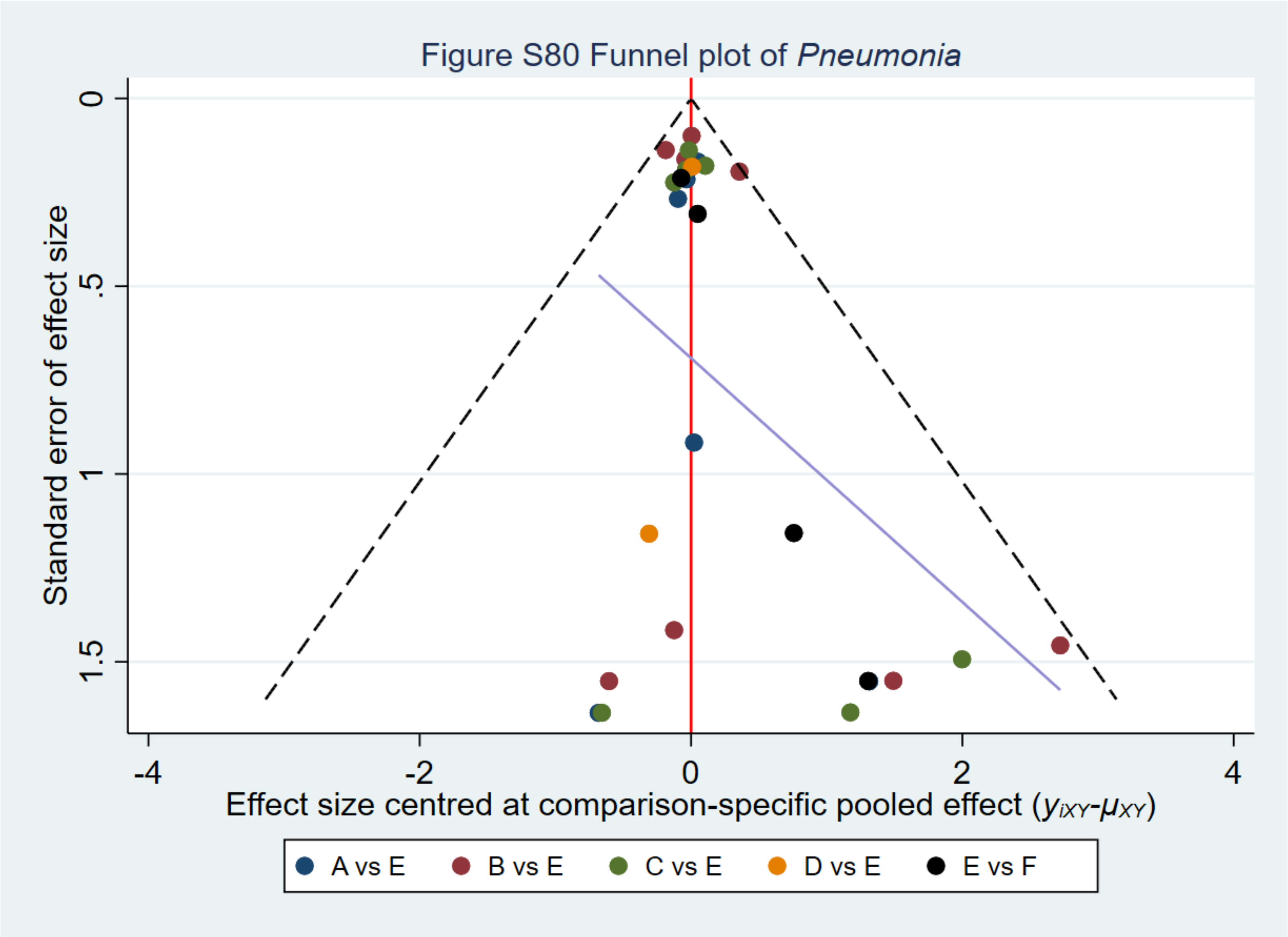

Figure S80 Funnel plot of *Pneumonia*
Standard error of effect size
0
.5
1
1.5
-4
-2
0
2
4
Effect size centred at comparison-specific pooled effect (y_{iXY}-μ_{XY})
A vs E
B vs E
C vs E
D vs E
E vs F

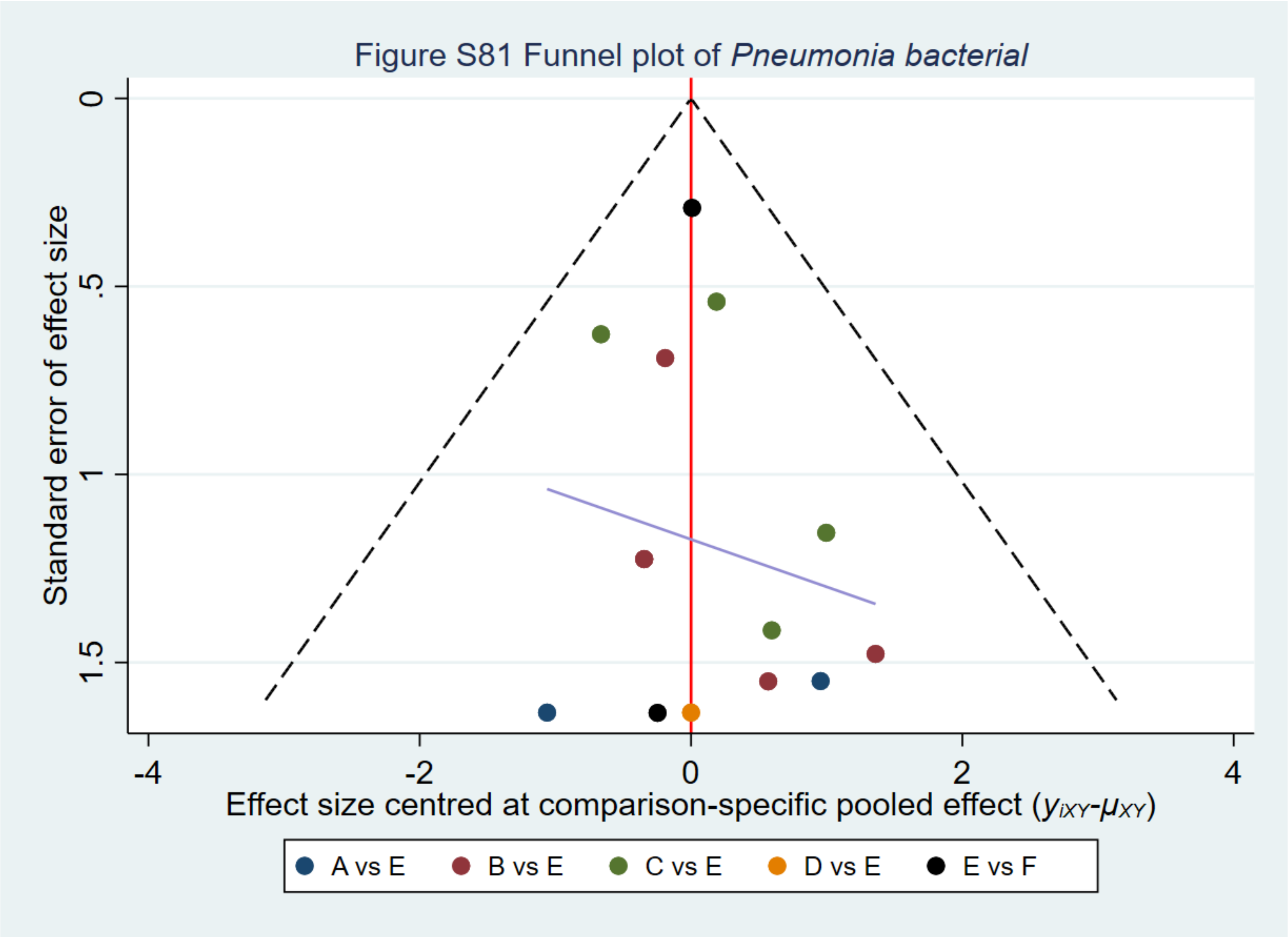

Figure S81 Funnel plot of *Pneumonia bacterial*
Standard error of effect size
0
.5
1
1.5
-4
-2
0
2
4
Effect size centred at comparison-specific pooled effect ($y_{iXY}-\mu_{XY}$)
A vs E
B vs E
C vs E
D vs E
E vs F
